# Vanillin production by *Corynebacterium glutamicum* using heterologous aromatic carboxylic acid reductases

**DOI:** 10.1186/s13068-024-02507-3

**Published:** 2024-05-01

**Authors:** Miku Matsuzawa, Junko Ito, Keiko Danjo, Keita Fukui

**Affiliations:** Research Institute for Bioscience Products & Fine Chemicals, Ajinomoto Co., Inc, Kawasaki, Kanagawa 210-8681 Japan

**Keywords:** Vanillin, Aromatic carboxylic acid reductase, *Corynebacterium glutamicum*, Biotransformation

## Abstract

**Background:**

Vanillin is a flavoring substance derived from vanilla. We are currently developing a biotransformation method for vanillin production using glucose. This report describes the last step in vanillin production: the conversion of vanillic acid to vanillin. First, we selected *Corynebacterium glutamicum* as the host owing to its high vanillin resistance. The aromatic aldehyde reductase gene (NCgl0324) and vanillic acid demethylase protein subunits A and B gene (*vanAB*, NCgl2300-NCgl2301) were deleted in *C. glutamicum* genome to avoid vanillin degradation. Next, we searched for an aromatic carboxylic acid reductase (ACAR), which converts vanillic acid to vanillin. Seventeen ACAR homologs from various organisms were introduced into *C. glutamicum.*

**Results:**

In vivo conversion experiments showed that eight ACARs were successfully expressed and produced vanillin. In terms of conversion activity and substrate specificity, the ACARs from *Gordonia effusa*, *Coccomyxa subellipsoidea*, and *Novosphingobium malaysiense* are promising candidates for commercial production.

**Conclusions:**

*Corynebacterium glutamicum* harboring *Gordonia effusa* ACAR produced 22 g/L vanillin, which is, to the best of our knowledge, the highest accumulation reported in the literature. At the same time, we discovered ACAR from *Novosphingobium malaysiense* and *Coccomyxa subellipsoidea* C-169 with high substrate specificity. These findings are useful for reducing the byproducts.

**Supplementary Information:**

The online version contains supplementary material available at 10.1186/s13068-024-02507-3.

## Background

Vanillin is a flavoring substance with a vanilla scent that is widely used in products such as food, beverages, cosmetics, and medicine. Vanillin is extracted from vanilla beans; however, due to the limited availability and high cost of vanilla, most vanillin in the market is available as a synthetic chemical. Furthermore, in response to the growing demand for natural products, the manufacturing method of vanillin by fermentation and enzymatic conversion is being developed as an alternative natural product to plant extracts [1].

The production of bio-vanillin by enzyme conversion using ferulic acid, eugenol, or isoeugenol as substrates is well known [2, 3]; however, the cost of raw materials is high. Therefore, it is preferable to establish a biotransformation method that uses glucose as the starting material. For vanillin production from glucose, an artificial synthetic route is generally used, wherein dehydroshikimic acid, an intermediate in the shikimate biosynthetic pathway, is dehydrated, methylated, and reduced to vanillin [1].

**Fig. 1:**
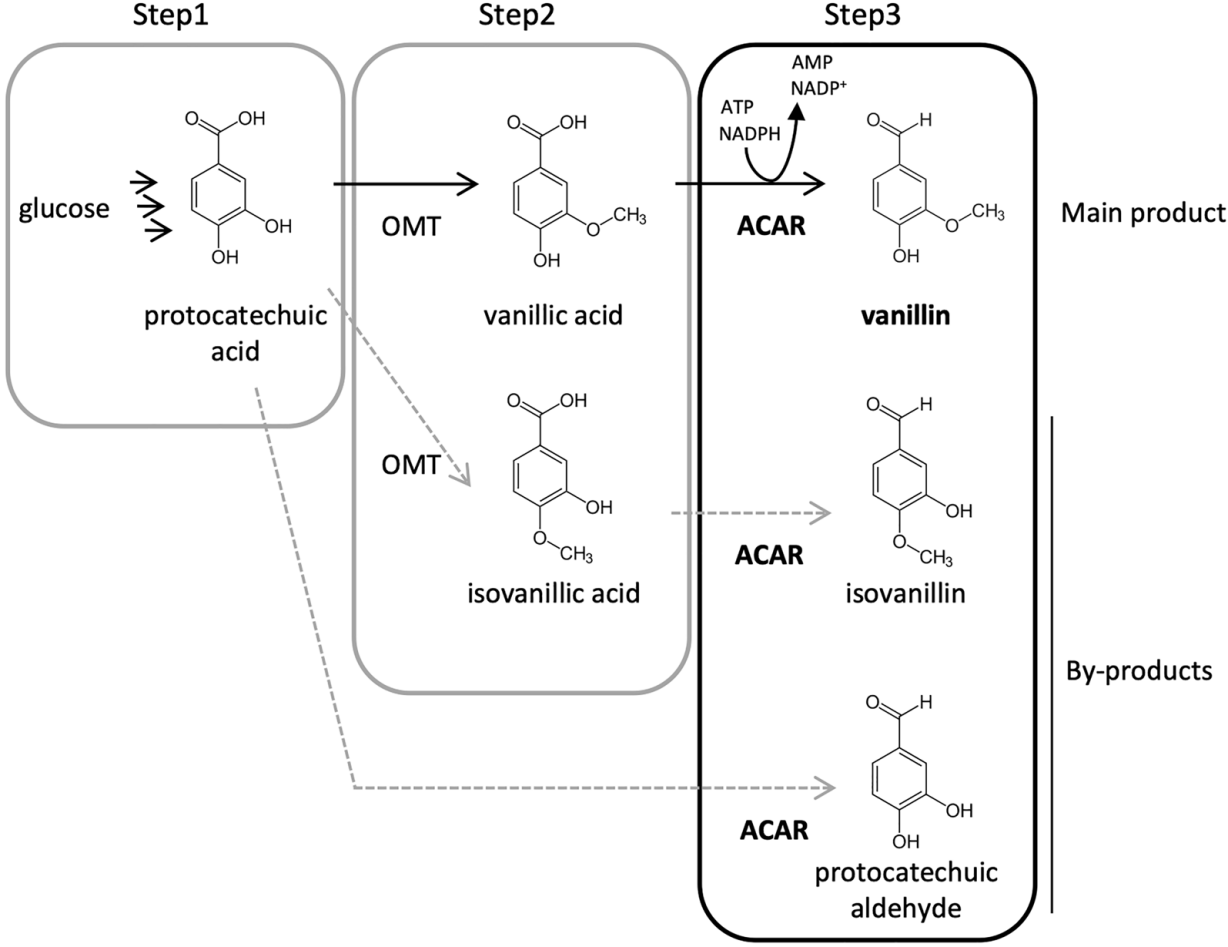
Production method of vanillin using the 3-step method. O-methyltransferase (OMT) produces vanillic acid and isovanillic acid from protocatechuic acid. Aromatic carboxylic acid reductase (ACAR) produces vanillin, isovanillin, and protocatechuic aldehyde from vanillic acid, isovanillic acid, and protocatechuic acid, respectively

We developed a vanillin production method from glucose using the “3-step method” (Fig. 1), wherein vanillin biosynthesis is achieved via three processes: (1) protocatechuic (PC) acid fermentation from glucose; (2) PC acid methylation to vanillic acid; and (3) reduction of the carboxyl group of vanillic acid to aldehyde to finally yield vanillin. Each step is performed by different bacterial strains. The methylation reaction in the second step requires the optimization of the entire metabolic flux to increase the supply of S‐adenosylmethionine, a methyl group donor, and high productivity is obtained by separating this step from the other reactions. The reduction reaction in the third step is separated because vanillin strongly inhibits bacterial growth and metabolism.

O-Methyltransferase (OMT) catalyzes the methylation of the hydroxyl group at the 3-position of PC acid [1]. As OMT activity was low and it was difficult to increase the supply of S-adenosylmethionine, PC acid remained in the medium at the end of the second step. At the same time, since the specificity of OMT is low, isovanillic acid was formed by the methylation of the hydroxyl group at the 4-position [2, 4, 5].

The reduction of vanillic acid to aldehydes is catalyzed by aromatic carboxylic acid reductase (ACAR) [2, 6]. However, these ACARs have low substrate specificity [7], and there is a high possibility that the remaining PC acid in the reaction solution and the isovanillic acid generated by the side reaction of the second step are also converted to aldehydes. As these compounds are difficult to remove in the later purification step and cause undesirable odors, it is necessary to use vanillic acid-specific ACAR.

Kim et al*.* reported a method for vanillin production from 4-hydroxybenzoate using *Corynebacterium glutamicum* [8]*.* They showed that the deletion of NCgl0324 in *C. glutamicum* decreased vanillyl alcohol production to 27% of that of the parent strain but did not reach 0%. In addition, more PC aldehydes were produced than vanillic acid due to the low substrate specificity of ACAR.

The third step is described in this report. *C. glutamicum* was found to be vanillin resistant, and the deletion of aromatic aldehyde reductase (AAR) prevented the conversion of vanillin to vanillyl alcohol. We also screened ACAR homologs from various organisms and identified ACARs with high conversion speed and substrate specificity.

## Methods

### Bacterial strains and plasmid construction

The strains used in the present study are listed in Table 1. The detailed methods for bacterial strain plasmid construction are described in the see Additional file 1.

**Table 1:** Strains used in this study

Strains	Source or references
*Escherichia coli* K-12 MG1655	[9]
*Corynebacterium glutamicum* 2256	[10]
*Saccharomyces cerevisiae* S288C	[11]
*Escherichia coli* JM109	Nippon Gene
*Escherichia coli* JM109 Δ*yqhD*	This work
*C. glutamicum* 2256 Δ*vanABK* (FKS0165)	This work
*C. glutamicum 2256* Δ*vanABK* ΔNCgl0313	This work
*C. glutamicum 2256* Δ*vanABK* ΔNCgl2709	This work
*C. glutamicum 2256* Δ*vanABK* ΔNCgl0324	This work
*C. glutamicum 2256* Δ*vanABK* ΔNCgl2709 ΔNCgl0324	This work
*C. glutamicum 2256* Δ*vanABK* ΔNCgl0313 ΔNCgl2709	This work
*C. glutamicum 2256* ΔvanABK ΔNCgl0313 ΔNCgl0324	This work
*C. glutamicum 2256* ΔvanABK ΔNCgl2709 ΔNCgl0324 ΔNCgl0313 (FKFC14)	This work
pVK9-Ptuf-Nb_ACAR-entD pVS7-vanK/FKFC14	This work
pVK9-Ptuf-An_ACAR-entD pVS7-vanK/FKFC14	This work
pVK9-Ptuf-Nc_ACAR-entD pVS7-vanK/FKFC14	This work
pVK9-Ptuf-Rw_ACAR-entD pVS7-vanK/FKFC14	This work
pVK9-Ptuf-Ss2_ACAR-entD pVS7-vanK/FKFC14	This work
pVK9-Ptuf-Aa_ACAR-entD pVS7-vanK/FKFC14	This work
pVK9-Ptuf-Fs_ACAR-entD pVS7-vanK/FKFC14	This work
pVK9-Ptuf-Ge_ACAR-entD pVS7-vanK/FKFC14	This work
pVK9-Ptuf-Ss1_ACAR-entD pVS7-vanK/FKFC14	This work
pVK9-Ptuf-Ka_ACAR-entD pVS7-vanK/FKFC14	This work
pVK9-Ptuf-Sc_ACAR-entD pVS7-vanK/FKFC14	This work
pVK9-Ptuf-Nm_ACAR-entD pVS7-vanK/FKFC14	This work
pVK9-Ptuf-Mm_ACAR-entD pVS7-vanK/FKFC14	This work
pVK9-Ptuf-Ms_ACAR-entD pVS7-vanK/FKFC14	This work
pVK9-Ptuf-Lm_ACAR-entD pVS7-vanK/FKFC14	This work
pVK9-Ptuf-Cs2_ACAR-entD pVS7-vanK/FKFC14	This work

### Comparison of vanillin resistance and vanillin degradation

LB medium was used for *Escherichia coli*, CM2B medium (10 g/L polypeptone, 10 g/L yeast extract, 5 g/L NaCl, 10 μg/L biotin, pH 7.0 adjusted with KOH) for *C. glutamicum*, and YPD medium (10 g/L yeast extract, 20 g/L Bacto peptone, 20 g/L glucose) for *Saccharomyces cerevisiae*. Culture temperature was set as 30 °C (*S. cerevisiae*), 31.5 °C (*C. glutamicum*), or 37 °C (*E. coli*). A 20 μL aliquot of each glycerol stock of the strains was applied to each agar medium and cultured for 20 h as pre-culture. The obtained cells were washed and suspended in sterile physiological saline. The optical density (OD) at 620 nm (OD_620nm_) of the cell suspension was measured, and the cell suspension was inoculated into an L-shaped test tube containing 4 mL medium (initial OD_620nm_: 0.02 for “Comparison of vanillin tolerance” and “Identification of the aromatic aldehyde reductase (AAR) that converts vanillin to vanillyl alcohol”, and 0.2 for “Evaluation of vanillin tolerance of FKFC14 strain”). Culturing was performed using a culture apparatus equipped with an automatic OD measurement function (TVS062CA ADVANTEC). OD_660nm_ was measured every 15 min. For “Comparison of vanillin tolerance”, 0, 1, or 2 g/L vanillin was added to the medium before inoculation. For “Identification of the aromatic aldehyde reductase (AAR) that converts vanillin to vanillyl alcohol”, 1 g/L vanillin was added to the medium before inoculation. For “Evaluation of vanillin tolerance of FKFC14 strain”, 0, 3, or 6 g/L of vanillin was added to the medium in the middle of the log phase. Specific growth rates (μ) were calculated according to the following equation: ln X_t_ = ln X_0_ + μ, where X_t_ and X_0_ are optical density measurements at time t and time 0, respectively.

### Comparison of vanillin, protocatechuic aldehyde, and isovanillin production by *C. glutamicum* vanillin-producing strains

A 20-μL aliquot of each glycerol stock of the constructed vanillin-producing strains was applied to the CM-Dex SGFC agar medium (2.5 g/L glucose, 2.5 g/L fructose, 10 g/L polypeptone, 10 g/L yeast extract, 1 g/L KH_2_PO_4_, 0.4 g/L MgSO_4_·7H_2_O, 0.01 g/L FeSO_4_·7H_2_O, 0.01 g/L MnSO_4_·7H_2_O, 2 g/L disodium succinate hexahydrate, 4 g/L sodium gluconate, 3 g/L urea, 1.2 g/L soybean hydrolysate, 10 µg/L biotin, and 15 g/L agar, adjusted to pH 7.5 with NaOH) containing 25 µg/mL of kanamycin and 50 µg/mL of spectinomycin and cultured at 31.5 °C for 20 h as pre-culture. The cells were suspended in sterile physiological saline. The OD of the cell suspension was measured, and the cell suspension was diluted with physiological saline to an OD_620nm_ of 83. A 1.5-mL aliquot of the diluted cell suspension was inoculated into 3.5 mL of a production medium [42.9 g/L of vanillic acid, PC acid, or isovanillic acid, 85.7 g/L glucose, 10 g/L polypeptone, 10 g/L yeast extract, 1 g/L KH_2_PO_4_, 0.4 g/L MgSO_4_·7H_2_O, 0.01 g/L FeSO_4_·7H_2_O, 0.01 g/L MnSO_4_·7H_2_O, 3 g/L urea, 1.2 g/L soybean hydrolysate, and 10 µg/L biotin, adjusted to pH 7.4 with KOH, and then mixed with 8.6 g/L CaCO_3_ (sterilized with hot air at 180 °C for 3 h)] containing 25 µg/mL of kanamycin and 50 µg/mL of spectinomycin, contained in a test tube, and cultured at 30 °C with shaking for 21 h.

### Vanillin production in jar fermentor

A 20-μL aliquot of each glycerol stock of the constructed vanillin-producing strains was applied to the CM-Dex SGFC agar medium and cultured at 31.5 °C for 20 h as pre-culture. The cells were suspended in sterile physiological saline. The OD of the cell suspension was measured, and the cell suspension was diluted with physiological saline to an OD_620nm_ of 100. A 100 μL aliquot of the diluted cell suspension was inoculated into 200 mL of CM-Dex SGFC liquid medium containing 25 µg/mL of kanamycin and 50 µg/mL of spectinomycin in a Sakaguchi-flask and cultured at 31.5 °C with shaking for 20 h as seed culture. The resulting culture medium was centrifuged, and the supernatant was removed. The cells were resuspended in sterile physiological saline. The OD of the cell suspension was measured, and the cell suspension was diluted with physiological saline to an OD_620nm_ of 84. A 90-mL aliquot of the diluted cell suspension was inoculated into 210 mL of vanillin production medium (without CaCO_3_) containing 25 µg/mL of kanamycin and 50 µg/mL of spectinomycin. The conversion reaction was aerobically conducted with 300 mL/min aeration; the culture temperature was 34 °C. The culture pH was maintained at 7.2 with ammonia gas. BSS-01NP fermentor (ABLE Co., Tokyo, Japan) was used to control and monitor pH, temperature, and dissolved oxygen concentration. The oxygen and CO_2_ concentrations in the exhausted-gas were measured every hour with an exhaust oxygen CO_2_ meter Model EX-1562–1 (Able & Biott Co., Tokyo, Japan).

### Analysis

After culture completion, the residual glucose concentration in the medium was analyzed using a Biotech Analyzer AS-310 (Sakura SI). The cell density (measured as OD) was assessed using a spectrophotometer U-2900 (HITACHI). The amounts of substrates and products (vanillic acid, PC acid, isovanillic acid, vanillin, PC aldehyde, isovanillin, and vanillyl alcohol) were analyzed using the ultra-performance liquid chromatography (UPLC) NEXERA X2 System (SHIMADZU), equipped with a KINETEX column (2.6 μm XB-C18, 150 × 30 mm, Phenomenex). Buffer A (0.1% [v/v] trifluoroacetic acid solution) and buffer B (0.1% [v/v] trifluoroacetic acid, 80% [v/v] acetonitrile) were used as the mobile phase, and compounds were eluted at 40 °C and a flow rate of 1.5 mL/min, with increasing concentrations of buffer B as follows: 10–20%, 0–3 min. The eluted compounds were detected by measuring their absorbances at 290, 315, and 350 nm. SDS-PAGE was performed using Bolt^®^ 4–12% Bis–Tris Plus 15 well (Thermo Fisher Scientific) with NuPAGE® SDS 2-(N-morpholino) ethane sulfonic acid (MES) running buffer and NuPAGE® Antioxidant (Thermo Fisher Scientific), according to the manufacturer’s instructions. The protein bands were stained using SimplyBlue SafeStain (Thermo Fisher Scientific), and the gel images were processed using Calibrated Densitometer GS-800 (Bio-Rad).

## Results

### Comparison of vanillin tolerance

The relative specific growth rates in the presence of vanillin were compared to determine which parent strain was suitable for vanillin production. *E. coli* MG1655, *C. glutamicum* 2256, and *S. cerevisiae* S288C were inoculated into media containing vanillin (0, 1, 2, and 3 g/L) and cultured in L-shaped test tubes. OD was measured, and the maximum specific growth rates (μ_max_) in the logarithmic growth phase were calculated. The relative μ_max_, when the growth rate of each strain in the absence of vanillin was set to 100, are shown in Table 2. In the presence of 1 g/L of vanillin, the relative μ_max_ of *E. coli*, *C. glutamicum*, and *S. cerevisiae* were 46, 59, and 26, respectively. In the presence of 2 g/L of vanillin, the relative μ_max_ of *E. coli* and *C. glutamicum* were 13 and 30, respectively. In the presence of 3 g/L vanillin, the relative μ_max_ of *E. coli* and *C. glutamicum* were 3 and 25, respectively. *S. cerevisiae* did not grow in the presence of 2 or 3 g/L vanillin. Even in the presence of 3 g/L vanillin, *C. glutamicum* maintained μ_max_ of 25% of that in the absence of vanillin, and this growth rate was higher than that of the other two species. Therefore, we selected *C. glutamicum,* which showed the strongest vanillin resistance among tested strain and specific rich medium.

**Table 2:** Relative maximum specific growth rates (μ_max_) in the presence of vanillin

	Vanillin concentration			
	0 g/L	1 g/L	2 g/L	3 g/L
*E. coli* MG1655	100	46	13	3
*C. glutamicum* 2256	100	59	30	25
*S. cerevisiae* S288C	100	26	0	0

Relative μ_max_ in the presence of 0, 1, 2, and 3 g/L vanillin. Culture media and temperatures are as follows: LB, 30 °C (*Escherichia coli* MG1655); CM2B, 31.5 °C (*Corynebacterium glutamicum* 2256); YPD, 30 °C (*Saccharomyces cerevisiae* S288C). Values represent the relative μ_max_ when the μ_max_ of each strain in the absence of vanillin was set to 100

### Identification of the aromatic aldehyde reductase (AAR) that converts vanillin to vanillyl alcohol

**Fig. 2:**
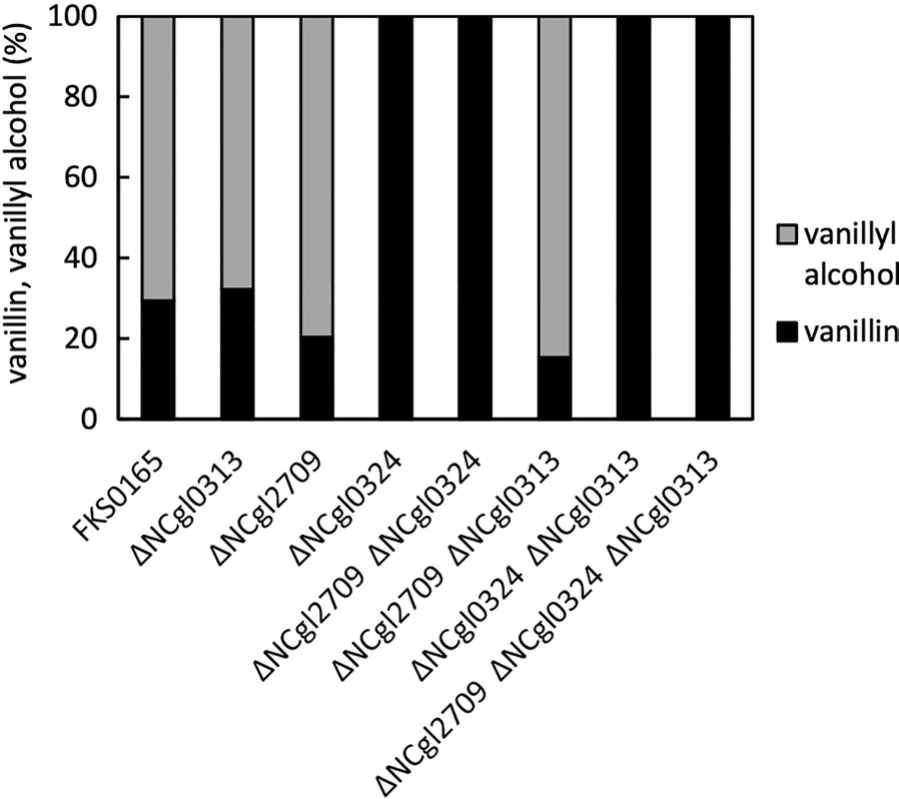
Vanillin conversion activity in aromatic aldehyde reductase (AAR) homolog-deficient *Corynebacterium glutamicum*. Single, double, and triple AAR homolog-deficient strains were incubated in CM2B medium containing 1 g/L vanillin for 24 h at 31.5 °C with shaking. The produced vanillyl alcohol and residual vanillin were quantified using UPLC. Black bars represent the percentage of residual vanillin {vanillin (mM) / [vanillin (mM) + vanillyl alcohol (mM)]}, and gray bars represent vanillyl alcohol {vanillyl alcohol (mM)/[vanillin (mM) + vanillyl alcohol (mM)]}

*Corynebacterium glutamicum* FKS0165, which is deficient in the *vanABK* gene, was used as the parent strain for subsequent experiments. It has been reported that vanillic acid is metabolized to PC acid and then assimilated in *C. glutamicum* [12]. *vanA* and *vanB* encode vanillic acid demethylase protein subunits A and B, respectively, and catalyze the reaction from vanillic acid to PC acid. *vanK* is a vanillic acid importer gene. Therefore, a *vanABK*-lacking strain was used to prevent substrate degradation. Since *vanABK* had an operon structure [13], it was difficult to delete *vanAB* and leave only *vanK* with the original transcription level. Therefore, we decided to delete the entire operon and introduce *vanK* plasmid later. Subsequently, FKS0165 was cultured in CM-Dex medium containing 1 g/L vanillin, and the supernatant was analyzed using UPLC. As a result, it was found that most of the vanillin was converted to vanillyl alcohol. Kim et al*.* reported that the deletion of aromatic aldehyde reductase (AAR, NCgl0324) in strain GAS355 (the chassis strain for vanillin production derived from *C. glutamicum* ATCC13032) decreased vanillyl alcohol production to 27% of that of the parent strain but did not reach 0% [8]. Our parent strain *C. glutamicum* 2256 had three AAR homologs (NCgl0313, NCgl0324, and NCgl2709). Therefore, to obtain a strain that has completely lost AAR activity, strains lacking one, two, or all three genes were constructed and grown in the presence of 1 g/L vanillin, and vanillyl alcohol and vanillin were measured. The total molar concentrations of vanillyl alcohol and vanillin were set to 100, and the abundance ratio (%) of each was calculated. The results are shown in Fig. 2. To be partly different from expectation, strains containing the NCgl0324 deletion completely lost their ability to produce vanillyl alcohol. Deletion of NCgl0313 and NCgl2709 did not cause a significant change in vanillyl alcohol production. These results demonstrated that NCgl0324 is the only AAR responsible for vanillyl alcohol production in *C. glutamicum* 2256. As the growth rate of the triple-deficient strain (FKFC14) did not decrease compared with that of the parent strain (Additional file 2: Fig. S1), FKFC14 was used for subsequent experiments.

### Evaluation of vanillin tolerance of FKFC14 strain

**Fig. 3:**
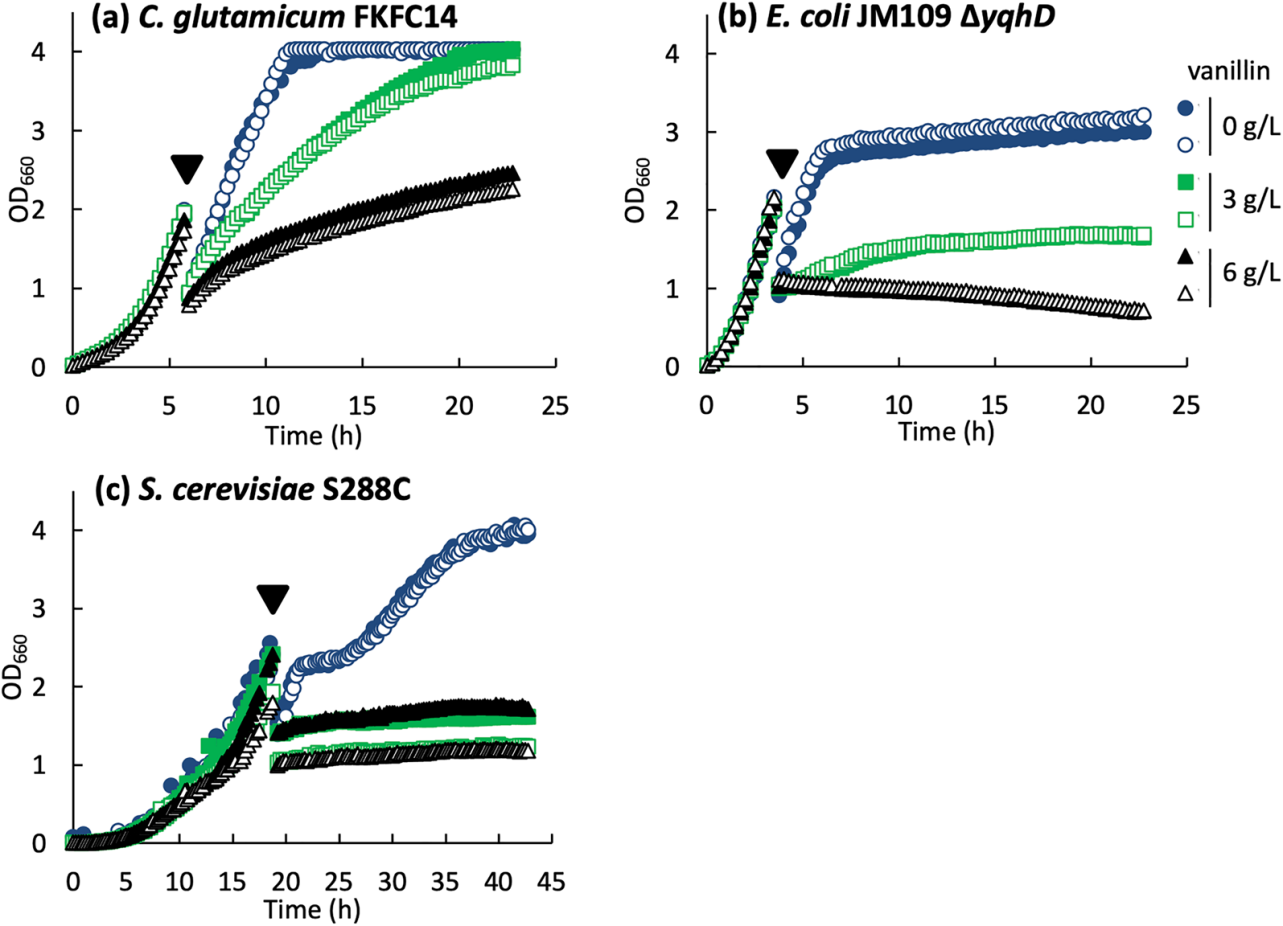
Influence of vanillin on growth. **a** *Corynebacterium glutamicum* FKFC14, **b** *Escherichia coli* JM109 Δ*yqhD*, and **c** *Saccharomyces cerevisiae* S288C were cultured in L-shape test tubes. Vanillin solution or distilled water was added during the log phase (▼) to achieve a final concentration of vanillin of 0 (open and closed blue circle), 3 (open and closed green square), and 6 g/L (open and closed black triangle). Experiments were performed in duplicate

Vanillin tolerance of FKFC14 was compared with that of *E. coli* JM109Δ*yqhD* and *S. cerevisiae* S288C, considering the possibility that vanillin resistance disappeared due to the loss of AAR because vanillin shows higher toxicity than vanillyl alcohols [14]. Kunjapur et al*.* reported that YqhD converts vanillin to vanillyl alcohol in *E. coli* MG1655 [15]. Our previous study showed that the deletion of *yqhD* in *E. coli* JM109 reduced vanillyl alcohol production to 20% of that of the parent strain JM109 (data not shown). JM109Δ*yqhD* was used here for comparison between AAR-deficient strains. To evaluate vanillin resistance under conditions close to those of vanillin production, the culture was initiated in a vanillin-free medium, and vanillin (final concentrations of 0, 3, and 6 g/L) was added during the logarithmic growth phase. As a result, in the presence of 3 g/L vanillin, their relative μ_max_ in JM109Δ*yqhD*, FKFC14, and *S. cerevisiae* S288C were 35, 62, and 13, respectively, when the rate of each strain in the absence of vanillin was set to 100 (Fig. 3). In the presence of 6 g/L vanillin, the relative μ_max_ were 0, 50, and 8, respectively. FKFC14 grew even in the presence of 6 g/L vanillin, whereas the growth of the other two species almost stopped. These results demonstrated that *C. glutamicum* FKFC14 still had a high resistance to vanillin, though it lost the ability to convert vanillin into vanillyl alcohol.

### Selection of ACAR homologs and construction of expression strains

ACAR homologs from various organisms have been screened to identify ACARs with high conversion activity and substrate specificity. ACAR homologs were extracted using BLAST. A TBLASTN search was performed using the amino acid sequence of *Nocardia iowensis* ACAR (Ni_ACAR) [6] as a query, and 16 ACAR homologs were selected to have a large variation in amino acid sequence identity to Ni_ACAR from a population with identity > 35% (Table 3). With the addition of the well-known Nc_ACAR (NcCAR) [16], in total, 17 ACAR homologous genes were selected. Codon usage of ACAR genes was optimized for *E. coli* because codon usage of *C. glutamicum* is similar to that of *E. coli*, and there have been many successful cases of *E. coli* gene expression in *C. glutamicum* [17, 18]. ACAR is known to become activated by transferring a phosphopantetheinyl group from coenzyme A to the catalytic center of ACAR via phosphopantetheinyl transferase (PPTase) [19]. *E. coli entD* encodes a PPTase, which converts ACAR into its active form [20]. As mentioned above, *vanK* (vanillic acid importer gene) was deleted along *vanAB*. The *vanK* plasmid was introduced to complement deletion so that the uptake rate was not a rate-limiting step. pVK9-Ptuf-ACAR-entD plasmids carrying the ACAR and *entD* genes from *E. coli* were constructed and introduced into the pVS7-Plac-vanK/FKFC14 strain, wherein the *vanK* gene was amplified.

**Table 3:** List of ACAR homologs

ACAR homolog name	Accession No.	Identity to *N. iowensis* ACAR (%)	Similarity to *N. iowensis* ACAR (%)	Species	Classification
Nb_ACAR	WP_051066691.1	75	84	*Nocardia brasiliensis*	Bacteria Gram-positive
Ka_ACAR	AHH98121.1	67	80	*Kutzneria albida* DSM 43870	Bacteria Gram-positive
Ms_ACAR	WP_015304658.1	64	78	*Mycobacterium* sp. JS623	Bacteria Gram-positive
Rw_ACAR	WP_037240929.1	62	76	*Rhodococcus wratislaviensis*	Bacteria Gram-positive
Ss1_ACAR	WP_037804860.1	63	75	*Streptomyces* sp. NRRL F-5135	Bacteria Gram-positive
Mm_ACAR	WP_012393886.1	62	75	*Mycobacterium marinum*	Bacteria Gram-positive
Fs_ACAR	ADP80669.1	56	70	*Frankia* sp. EuI1c	Bacteria Gram-positive
Aa_ACAR	WP_030430720.1	55	69	*Allokutzneria albata*	Bacteria Gram-positive
Ks_ACAR	WP_043477830.1	53	67	*Kitasatospora* sp. MBT66	Bacteria Gram-positive
Ss2_ACAR	WP_046193084.1	50	66	*Sphingomonas* sp. SRS2	Bacteria Gram-negative
Ge_ACAR	WP_007316327.1	45	64	*Gordonia effusa*	Bacteria Gram-positive
Nm_ACAR	WP_039289949.1	45	61	*Novosphingobium malaysiense*	Bacteria Gram-negative
An_ACAR	CBA74242.1	41	60	*Arsenophonus nasoniae*	Bacteria Gram-negative
Sc_ACAR	AGP42038.1	41	56	*Sorangium cellulosum* So0157-2	Bacteria Gram-negative
Cs2_ACAR	XP_005651684.1	38	56	*Coccomyxa subellipsoidea* C-169	Microalgae
Lm_ACAR	WP_028384907.1	36	56	*Legionella moravica*	Bacteria Gram-negative
Nc_ACAR	XP_955820.1	17	32	*Neurospora crassa* OR74A	Fungi

### Evaluation of vanillin conversion activity of ACAR homolog-introduced strains

**Fig. 4:**
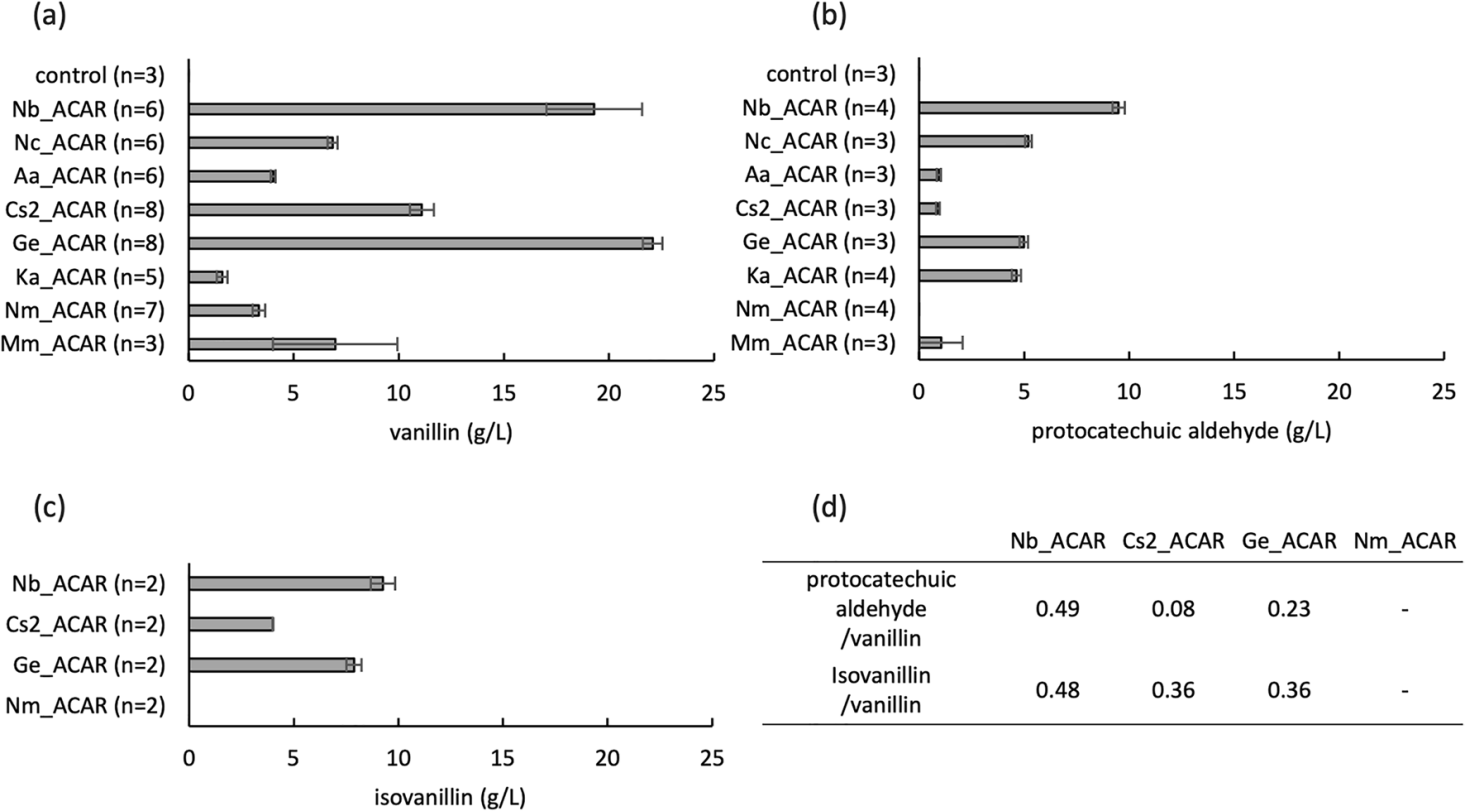
Aldehyde production by *Corynebacterium glutamicum* FKFC14 harboring aromatic carboxylic acid reductase (ACAR) genes. Bars represent the amount of vanillin produced from vanillic acid (**a**), protocatechuic aldehyde from protocatechuic acid (**b**), and isovanillin from isovanillic acid (**c**) after 21 h of incubation at 30 °C with shaking. Data are shown as mean ± standard error (SE). Control; pVK9-Ptuf-entD pVS7-vanK/FKFC14. The ratio of the amount produced was calculated by dividing the concentrations of protocatechuic aldehyde and isovanillin by that of vanillin (**d**)

**Fig. 5:**
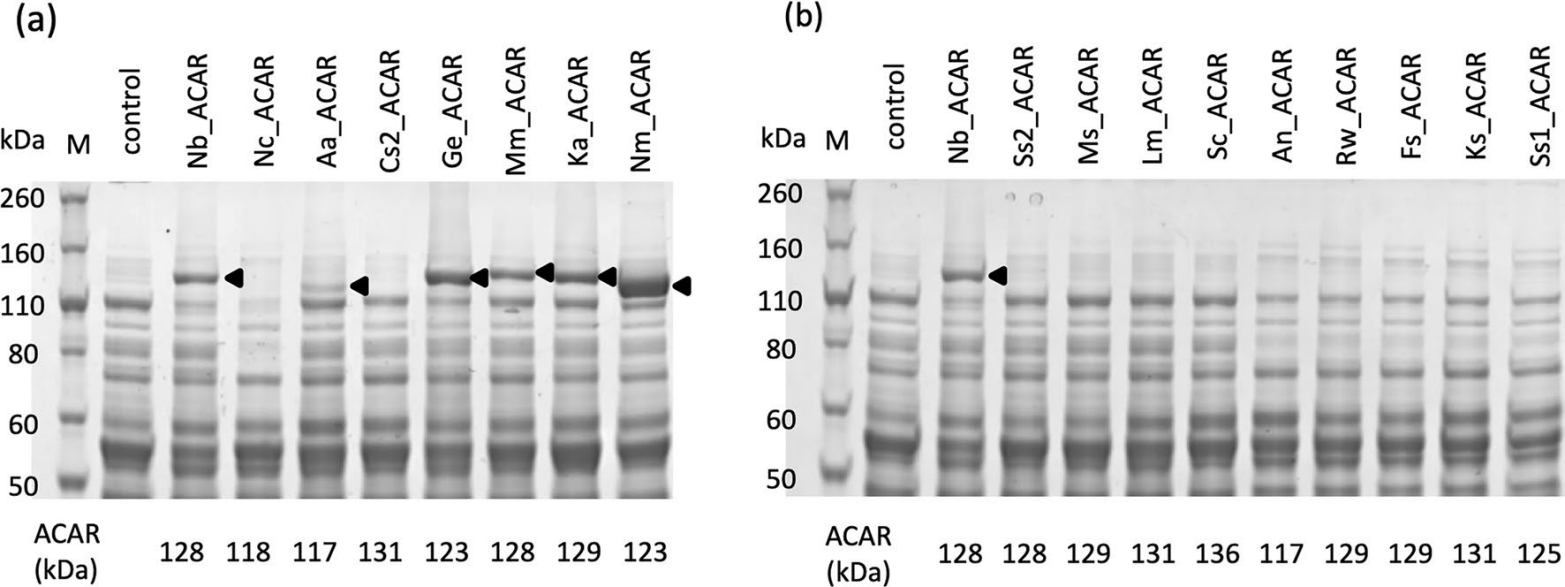
SDS-PAGE analysis of aromatic carboxylic acid reductase (ACAR) expression in* Corynebacterium glutamicum* FKFC14. Each lane contained 5 μg of crude protein extract. After electrophoresis, the gels were stained with Coomassie Brilliant Blue G-250. **a** Strains with vanillin-forming activity. **b** Strains lacking vanillin-forming activity. For comparison, pVK9-Ptuf-entD pVS7-vanK/FKFC14 (control) and pVK9-Ptuf-Nb_ACAR-entD pVS7-vanK/FKFC14 (Nb_ACAR) were used in both gels. M: protein molecular weight marker. The theoretical molecular weight of each ACAR is indicated under each lane

A total of 17 ACAR-expressing strains were cultured in CM-Dex SGFG medium containing 30 g/L vanillic acid for 21 h in a test tube. The supernatant was analyzed using UPLC. Vanillin production was observed in eight strains (Fig. 4a). FKFC14 harboring ACAR from *Nocardia brasiliensis* (Nb_ACAR), *Neurospora crassa* (Nc_ACAR), *Allokutzneria albata* (Aa_ACAR), *Coccomyxa subellipsoidea* C-169 (Cs2_ACAR), *Gordonia effuse* (Ge_ACAR), *Kutzneria albida* (Ka_ACAR), *Novosphingobium malaysiense* (Nm_ACAR), and *Mycobacterium marinum* (Mm_ACAR) produced 19 ± 2.3, 6.9 ± 0.24, 4.0 ± 0.11, 11 ± 0.57, 22 ± 0.46, 1.6 ± 0.26, 3.3 ± 0.30, and 7.0 ± 3.0 g/L vanillin, respectively. Vanillyl alcohol was not produced by any of the strains (data not shown). Cells obtained from the test tube culture were disrupted by ultrasonic treatment, and cell lysates were subjected to SDS-PAGE. For strains with vanillin-forming activity, ACAR bands were observed, except for Nc_ACAR and Cs2_ACAR (Fig. 5a). In contrast, ACAR bands were not observed in any strains without vanillin-forming activity (Fig. 5b).

### Substrate specificity of ACAR

To obtain highly purified vanillin in the purification process, it is important to reduce the side products. Particularly, PC aldehyde and isovanillin are difficult to remove during purification because their chemical properties are similar to those of vanillin.

Therefore, substrate specificity was examined for the strains in which vanillic acid was converted to vanillin. When the conversion reaction was performed using PC acid as a substrate, FKFC14 harboring Nb_ACAR, Nc_ACAR, Aa_ACAR, Cs2_ACAR, Ge_ACAR, Ka_ACAR, and Mm_ACAR produced 9.5 ± 0.28, 5.2 ± 0.16, 0.94 ± 0.10, 0.89 ± 0.083, 5.0 ± 0.20, 4.6 ± 0.23, and 1.0 ± 1.0 g/L PC aldehyde, respectively. Only Nm_ACAR did not produce PC aldehydes (Fig. 4b). Based on vanillin formation activity and substrate specificity, Nb_ACAR, Ge_ACAR, Cs2_ACAR, and Nm_ACAR were selected for further analysis. The conversion reaction was then performed using isovanillic acid as the substrate (Fig. 4c). FKFC14 harboring Nb_ACAR, Cs2_ACAR, and Ge_ACAR produced 9.3 ± 0.58, 4.0 ± 0.011, and 7.9 ± 0.36 g/L isovanillin, respectively. Vanillyl alcohol was not produced by any of the strains (data not shown). Again, only Nm_ACAR did not produce any isovanillin. The ratios of PC aldehyde and isovanillin production to vanillin production were compared (Fig. 2d). Cs2_ACAR had a lower PC aldehyde/vanillin ratio (0.08) than that of Ge_ACAR (0.23) and Nb_ACAR (0.49). Cs2_ACAR and Ge_ACAR exhibited lower isovanillin/vanillin ratios (0.36) than that of Nb_ACAR (0.48).

### Vanillin production in jar fermentor

**Fig. 6:**
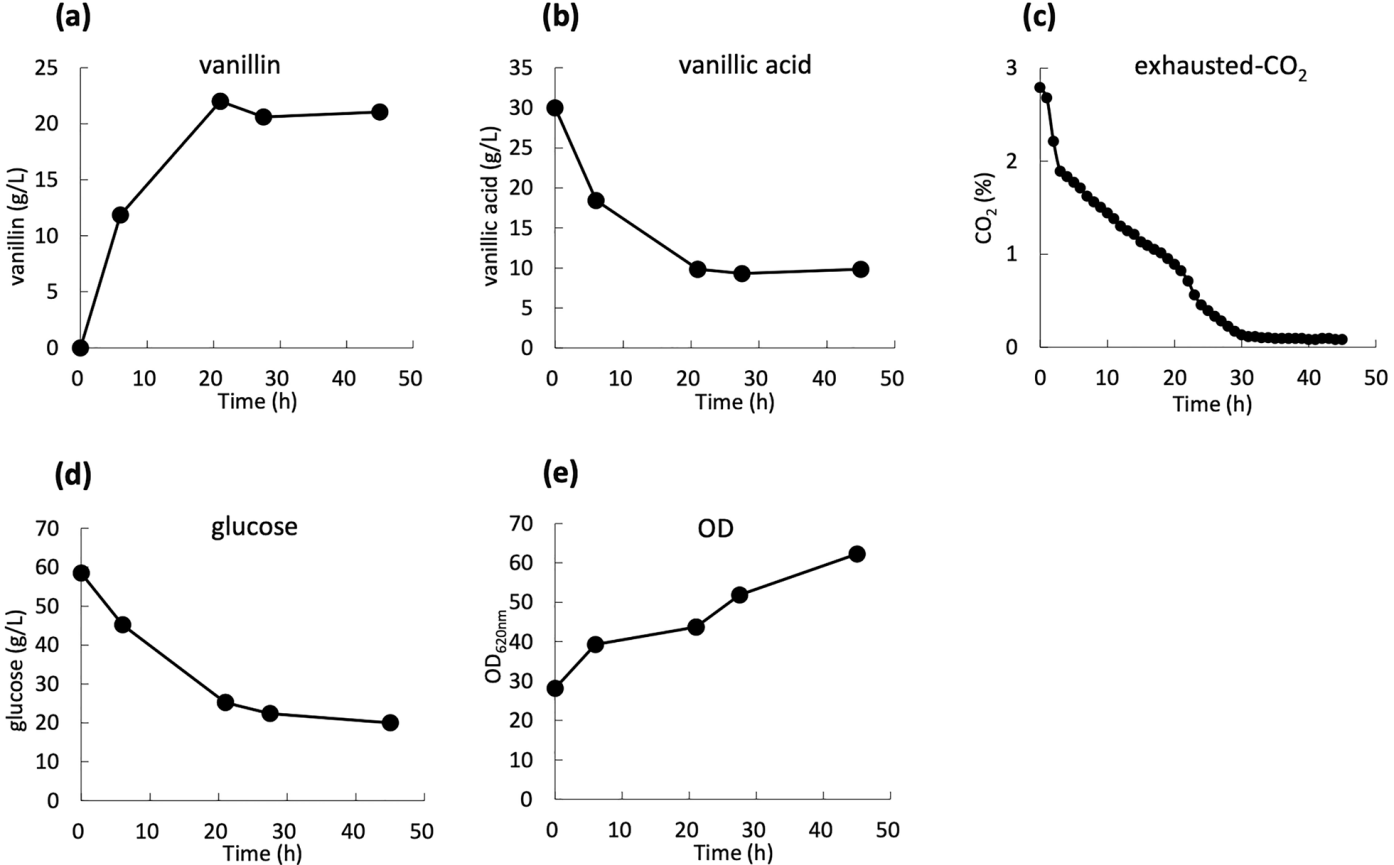
Vanillin production by pVK9-Ptuf-Ge_ACAR-entD pVS7-vanK/FKFC14 in the jar fermentor. Time course of vanillin production (**a**), vanillic acid consumption (**b**), exhausted-CO2 (**c**), glucose consumption (**d**), and optical density (**e**) are shown

A conversion reaction in a jar fermentor was performed using strain pVK9-Ptuf-Ge_ACAR-entD pVS7-vanK/FKFC14 for time course analysis. The reaction medium and initial OD_620nm_ were set to the same as that of the reaction in the test tube. As a result, the vanillin accumulation reached 21 g/L at the reaction time of 21 h (Fig. 6a). The conversion reaction proceeded to a vanillin concentration of 12 g/L in the first 6 h, and then slowed down and almost completely stopped at 21 h with 10 g/L of vanillic acid remaining (Fig. 6b). The rate of sugar consumption also decreased with the incubation time (Fig. 6d). The exhaust CO_2_ concentration was also measured every hour to investigate the respiratory activity of the bacteria. It dropped sharply to 65% of the value at the beginning of the reaction by 4 h, and then dropped proportionally to time to 5% at 30 h (Fig. 6c).

## Discussion

Host candidates (*E. coli* K-12, *C. glutamicum*, and *S. cerevisiae*) were selected because some of their fermentative products are Generally Recognized As Safe (GRAS), they grow fast, and their genetic manipulation is straightforward. When the growth in the presence of vanillin was examined for the purpose of comparing vanillin resistance, *C. glutamicum* maintained a μ_max_ of 25% of that in the absence of vanillin, even in the presence of 3 g/L vanillin.

*Corynebacterium glutamicum* converted most of the vanillin in the medium to vanillyl alcohol. To prevent the decomposition of vanillin, AAR homolog-deficient strains (NCgl0313, NCgl0324, and NCgl2709) were prepared. As a result, the NCgl0324-deficient strain did not produce vanillyl alcohol. Kim et al*.* reported that the deletion of NCgl0324 in *C. glutamicum* GAS355 derived from ATCC13032 decreased vanillyl alcohol production to 27% of the parent strain but did not reach 0% [8]. It is thought that there are one or more vanillin-reducing enzymes in strain ATCC13032 but not in strain 2256.

Vanillin resistance in *C. glutamicum* is thought to be due to the conversion of vanillin to the less toxic vanillyl alcohol or vanillic acid [21]. However, even when the conversion enzyme is lost, it showed higher vanillin resistance relative to *E. coli* JM109Δ*yqhD* and *S. cerevisiae. C. glutamicum* is known to induce the expression of various stress-tolerant factors, such as oxidative stress tolerance factors, in the presence of vanillin [22], which are thought to contribute to the maintenance of growth ability in the presence of vanillin.

Screening was performed to identify ACARs that have high vanillin formation activity, are sufficiently expressed in the host, and do not produce isovanillin or PC aldehyde, which are difficult to remove during the purification process. The rate of substance production was determined based on the specific activity of the enzyme and its expression levels. Not only the nature of the enzyme, but also the expression level in the selected host is a crucial factor. For evaluation under conditions similar to practical use, the properties of the enzyme were evaluated via a conversion reaction using a cell body.

By evaluating the activity and specificity of ACARs in various species from the population obtained by TBLASTN, using Ni_ACAR as a query, we identified ACARs with various characteristics. Of the 17 ACARs evaluated, eight showed vanillin formation activity, and nine did not. Since no ACAR bands were observed in the SDS-PAGE of the cell lysates of the strains that showed no activity, they might not be expressed in *C. glutamicum*. There was also the possibility that some of the nine ACARs were slightly expressed but did not exhibit vanillin formation activity because Cs2_ACAR and Nc_ACAR bands were not observed by SDS-PAGE, although vanillin was produced.

Among the eight ACARs whose activity was confirmed, Nc_ACAR (NcCAR) and Mm_ACAR has already been reported to form vanillin from vanillic acid [16, 23], but its activity against isovanillic acid and PC acid have not been reported.

Three types of ACARs were studied: Ge_ACAR, which had a high expression level and high conversion rate; Cs2_ACAR, which had high conversion ability despite its low expression level and low activity in producing PC aldehyde; and Nm_ACAR, which had high substrate specificity and high expression level, but low conversion rate. In general, ACAR has low substrate specificity and reduces carboxyl groups on various substrates [7]. Nm_ACAR had specific features that strictly identified the location and presence of methyl groups.

To measure specific activity, we attempted to overexpress His-tagged Ge_ACAR, Cs2_ACAR, and Nm_ACAR in *E. coli* but Cs2_ACAR did not express (data not shown). In the future, it would be desirable to perform enzymatic analyses via overexpression and purification.

Vanillin accumulation in pVK9-Ge_ACAR-entD pVS7-Plac-vanK/FKFC14 cells was 22 g/L. This amount is sufficient for commercial production. The highest vanillin accumulation ever reported is 2.86 g/L in *E. coli* expressing *Mycobacterium abscessus* CAR [23]. In the present study, we achieved a vanillin accumulation that was seven times the previously reported value. This was achieved using *C. glutamicum*, which has high vanillin resistance, and Ge_ACAR, which has high activity and expression levels.

No “perfect” ACAR—ACAR that has high vanillin formation activity, is sufficiently expressed in the host, and does not produce isovanillin or PC aldehyde—was found in this study. This issue can potentially be addressed by integrating the findings of this study to modify ACAR-encoding genes. Specifically, the following three methods were considered: (i) replacement of amino acid residues near the catalytic center of Ge_ACAR or Cs2_ACAR with reference to the sequence of Nm_ACAR to increase substrate specificity; (ii) optimization of the promoter, Shine–Dalgarno (SD) sequence, or codon usage to increase Cs2_ACAR expression; and (iii) replacement of amino acid residues near the catalytic center of Nm_ACAR with reference to the sequence of Cs2_ACAR or Ge_ACAR to increase specific activity.

For time course analysis and scale-up test, a conversion reaction in a jar fermentor was performed using strain pVK9-Ptuf-Ge_ACAR-entD pVS7-vanK/FKFC14. The vanillin accumulation reached 21 g/L at the reaction time of 21 h, almost the same as the result in test tube. The results obtained with the jar fermentor are often highly correlated with the commercial scale production in our experience. From this result, it was suggested that this conversion reaction can be further scaled up. The conversion reaction rate, respiratory activity, and sugar consumption rate were all the fastest immediately after the start of the reaction, and the rate decreased with time until almost stopped leaving the substrate at 21 h. There was a high possibility that the decrease in activity was due to the toxicity of aldehydes. The toxicity of aldehydes is thought to be mainly due to damage to proteins [24]. It was considered that aldehydes damaged proteins responsible for glucose metabolism, and the supply of ATP and NADPH, which are necessary for the ACAR reaction, was interrupted. One of the solutions will be to enhance vanillin secretion. To the best of our knowledge, no vanillin transporter has been identified; therefore, its identification will be the key to the improvement. Furthermore, due to the low substrate specificity, Ge_ACAR could convert various carboxylic acids in cells to aldehydes and exhibit toxicity. It was reported that *N. iowensis*-derived ACAR converts carboxylic acid abundant in cell (e.g., fatty acid, citric acid, and 2-ketoglutaric acid) [7], and Ge_ACAR could have similar properties. The growth of the Ge_ACAR-expressed strain (pVK9-Ptuf-Ge_ACAR-entD pVS7-vanK/FKFC14) was slower than that of the control strain (pVK9-Ptuf-entD pVS7-vanK/FKFC14 strain) even on a CM-Dex SGFC plate medium that did not contain vanillic acid (data not shown), which supported this hypothesis. If ACAR with high activity, high expression level, and high vanillic acid specificity is designed, it will contribute not only to avoiding the formation of undesirable byproducts, but also to high accumulation by reducing toxicity.

## Conclusion

In this study, we developed a method to efficiently convert vanillic acid into vanillin. Using *C. glutamicum* with high vanillin tolerance and deleting AAR (NCgl0324) and *vanABK* genes (NCgl2300-NCgl2302), we constructed a platform strain, FKFC14, capable of high accumulation of highly biotoxic vanillin. Vanillin (22 g/L in test tube, 21 g/L in jar fermentor) was produced from vanillic acid by strain FKFC14 harboring ACAR from *Gordonia effusa*, which showed high activity and was easily overexpressed in *C. glutamicum*. This technology enables the production of vanillin from glucose by combining with the production method of vanillic acid. At the same time, we discovered ACAR from *Novosphingobium malaysiense* and *Coccomyxa subellipsoidea* C-169 with high substrate specificity. These findings are helpful in reducing the byproducts.

### Supplementary Information

**Additional file 1.** Supplementary materials.

**Additional file 2: Figure S1.** Influence of aromatic aldehyde reductase (AAR) deletion on cell growth. FKS0165 (open and closed blue circle) and FKFC14 (open and closed green square) were grown in CM-Dex medium containing **a** 0 g/L vanillin and **b** 1 g/L vanillin. Experiments were performed in duplicate.

**Additional file 3: Figure S2.** Construction of ACAR-entD expression plasmids. pVK9-Nb_ACAR-entD was treated with Bgl II and Bps1407 I and was connected to two synthetic DNA fragments with partial sequences of P_*tuf*_ at the 3′ end, ACAR genes (codon-optimized to *Escherichia coli*), and partial sequence of *entD* at the 5′ end using the In-Fusion HD Cloning Kit.

## Data Availability

All data generated or analyzed during this study are included in this published article and its Additional files.

## Abbreviations

AAR: Aromatic aldehyde reductase
ACAR: Aromatic carboxylic acid reductase
μmax: Maximum specific growth rate
OMT: O-Methyltransferase
PC acid: Protocatechuic acid
PC aldehyde: Protocatechuic aldehyde
PPTase: Phosphopantetheinyl transferase
SD: Shine–Dalgarno
SDS-PAGE: Sodium dodecyl sulfate-polyacrylamide gel electrophoresis
UPLC: Ultra performance liquid chromatography
