# Supplementary material for: Vanillin production by Corynebacterium glutamicum using heterologous aromatic carboxylic acid reductases
Source: Biotechnol Biofuels Bioprod. 2024 May 1;17:58. doi: 10.1186/s13068-024-02507-3 (PMC11064420; doi:10.1186/s13068-024-02507-3)
Supplement: Supplementary file 1 — Additional file 1. Supplementary materials. [file 13068_2024_2507_MOESM1_ESM.docx]

**Additional File 1**

**Construction of the *Escherichia coli* JM109Δ*yqhD* strain**

The synthetic DNA primers used for the PCR are listed in Supp. Table 1. By using the DNA fragment of pMW118-attL-Cm-attR [1] as the PCR template and the synthetic DNAs (DyqhD_F and DyqhD_R) as the primers, a fragment for deleting the *yqhD* gene, consisting of an upstream open reading frame (ORF) region of the *yqhD* gene, attRλ sequence, chloramphenicol resistance gene, attLλ sequence, and downstream ORF region of the *yqhD* gene was obtained. Then, competent cells of *E. coli* JM109 (Takara Bio) were transformed with the pKD46 plasmid [2], applied to the LB medium containing 100 µg/mL of ampicillin, cultured overnight at 30 °C to obtain single colonies, and the *E. coli* JM109/pKD46 strain was obtained as a transformant. The *E. coli* JM109/pKD46 strain was cultured in LB medium containing 100 µg/mL of ampicillin and 50 mM arabinose, and the fragment for deleting the *yqhD* gene was introduced using the electric pulse method. Cells were applied to the LB agar medium containing 100 µg/mL of ampicillin and 25 µg/mL of chloramphenicol and cultured at 30 °C. Further, the obtained *E. coli* JM109Δ*yqhD*::CmR/pKD46 strain was cultured at 42 °C, and the *E. coli* JM109Δ*yqhD*::CmR strain, in which pKD46 was eliminated, was obtained based on the loss of ampicillin resistance. The *E. coli* JM109Δ*yqhD*::CmR strain was cultured in the LB medium containing 25 µg/mL of chloramphenicol, and the pMW-int-xis plasmid [1] was introduced using the electric pulse method. Cells were grown on LB agar medium containing 100 µg/mL of ampicillin and cultured at 30 °C to obtain colonies. Obtained colonies were cultured again at 30 °C on the agar medium, and colonies that proliferated on the LB agar medium containing 100 µg/mL of ampicillin but did not proliferate on the LB agar medium containing 100 µg/mL of ampicillin and 25 µg/mL of chloramphenicol were identified. The colonies were also cultured at 37 °C in parallel, and colonies that did not proliferate on the LB agar medium containing 100 µg/mL of ampicillin or 25 µg/mL of chloramphenicol but proliferated on the LB agar medium were selected to obtain a strain in which the chloramphenicol resistance gene and pMX-int-xis were eliminated.

**Construction of plasmids for the deletion of genes in the *Corynebacterium glutamicum* genome**

The upstream and N-terminal coding regions of the target gene for deletion were amplified by PCR using the genomic DNA of the *C. glutamicum* 2256 strain as the template. Separately, the downstream and C-terminal coding regions of the target gene were also amplified. These PCR products were mixed in approximately equimolar amounts and inserted into the pBS4S vector [3] treated with BamH I and Pst I using the In-Fusion HD Cloning Kit (Clontech). With this DNA, competent cells of *E. coli* JM109 were transformed, and the cells were applied to the LB medium containing 100 µM IPTG, 40 µg/mL of X-Gal, and 40 µg/mL of kanamycin and cultured overnight. White colonies were picked and separated into single colonies to obtain the transformants. Plasmids were extracted from the transformants.

**Deletion of genes in *C. glutamicum* genome**

Plasmids for deletion of the genes obtained above were introduced into the *C. glutamicum* 2256 strain using the electric pulse method. The cells were applied to the CM-Dex agar medium (5 g/L of glucose, 10 g/L of polypeptone, 10 g/L of yeast extract, 1 g/L of KH_2_PO_4_, 0.4 g/L of MgSO_4_·7H_2_O, 0.01 g/L of FeSO_4_·7H_2_O, 0.01 g/L of MnSO_4_·7H_2_O, 3 g/L of urea, 1.2 g/L of soybean hydrolysate, 10 µg/L of biotin, and 15 g/L of agar, adjusted to pH 7.5 with NaOH) containing 25 µg/mL of kanamycin, and cultured at 31.5 °C. It was confirmed that the strain was a once-recombinant strain by PCR, in which the plasmid was incorporated into the genome via homologous recombination. The once-recombinant strain was cultured overnight in the CM-Dex liquid medium, and the culture broth was applied to the S10 agar medium (100 g/L of sucrose, 10 g/L of polypeptone, 10 g/L of yeast extract, 1 g/L of KH_2_PO_4_, 0.4 g/L of MgSO_4_·7H_2_O, 0.01 g/L of FeSO_4_·7H_2_O, 0.01 g/L of MnSO_4_·4-5H_2_O, 3 g/L of urea, 1.2 g/L of soybean protein hydrolysate solution, and 20 g/L of agar, adjusted to pH 7.5 with NaOH, and autoclaved at 120 °C for 20 min), and cultured at 31.5 °C. Among the colonies that appeared, one strain showing kanamycin susceptibility was purified on CM-Dex agar to obtain a twice-recombinant strain. Target gene deletion was confirmed by PCR.

**Construction of plasmid pVK9-ACAR-entD**

First, the pVK9-Nb_ACAR-entD plasmid was constructed. PCR was performed using the genomic DNA of the *C. glutamicum* 2256 strain as the template and synthetic DNAs (Ptuf-F and Ptuf-R) as primers to obtain a PCR product containing the promoter region and Shine–Dalgarno (SD) sequence of the elongation factor Tu gene. PCR was performed using the synthetic ACAR gene of *Nocardia brasiliensis* provided by ATG Service Gen (Russian Federation, Saint-Petersburg) as the template and synthetic DNAs (Nb_ACAR-F and Nb_ACAR-R) as the primers to obtain a PCR product containing the ORF of the car gene. PCR was performed using the genomic DNA of the *E. coli* MG1655 strain as the template and synthetic DNAs (entD-F and entD-R) as primers to obtain a PCR product containing the ORF, SD, and downstream sequence of the entD gene. These fragments were then inserted into the pVK9 vector [4] and treated with BamH I and Pst I using an In-Fusion HD Cloning Kit. pVK9 is a shuttle vector for coryneform bacteria and *E. coli*. By using this DNA, competent cells of *E. coli* JM109 were transformed, and the cells were applied to the LB medium containing 100 µM IPTG, 40 µg/mL of X-Gal, and 25 µg/mL of kanamycin, and cultured overnight. White colonies were picked and separated into single colonies to obtain transformants. Plasmids were extracted from the obtained transformants, and the plasmid in which the target PCR product was inserted was designated pVK9-Nb_ACAR-entD. In pVK9-Nb_ACAR-entD, car and entD genes constitute an operon structure expressed by the tuf promoter.

The method used to construct ACAR-entD plasmids, other than pVK9-Nb_ACAR-entD, was shown in Additional file 3: Fig. S2. Synthetic DNA fragments of partial sequence on the 3' side of Ptuf, ACAR genes (codon-optimized to *E. coli*), and partial sequence on the 5' side of *entD* were provided by Thermo Fisher Scientific. These two fragments were inserted into pVK9-Nb_ACAR-entD treated with Bgl II and Bps1407 I using the In-Fusion HD Cloning Kit. By using this DNA, competent cells of *E. coli* JM109 were transformed, and the cells were applied to the LB medium containing 25 µg/mL of kanamycin and cultured overnight. The colonies were picked and separated into single colonies to obtain the transformants. Plasmids were extracted from the obtained transformants, and those in which the target PCR product was inserted were selected. The codon-optimized ACAR sequences are listed below. GeneOptimizer Algorithm provided by Thermo Fisher Scientific was used for gene optimization.

Nb_ACAR

ATGGCAACTGACAGCAGGAGCGACCGTCTACGTAGGCGGATAGCTCAGCTATTTGCAGAAGATGAACAGGTTAAGGCAGCAGTTCCGGATCAGGAAGTTGTTGAAGCAATTCGTGCACCTGGTCTGCGTCTGGCTCAGATTATGGCAACCGTGATGGAACGTTACGCAGATCGTCCTGCAGTTGGGCAGCGTGCAAGCGAACCGGTTACCGAAAGCGGGCGTACCACCTTTCGTCTGTTACCGGAGTTTGAAACCCTGACCTATCGTGAACTGTGGGCACGTGTGCGTGCAGTTGCAGCAGCATGGCATGGGGACGCAGAGAGGCCCTTACGTGCAGGAGACTTTGTCGCTCTGCTGGGGTTTGCAGGTATTGACTATGGTACCTTAGACTTAGCAAATATTCACCTGGGTTTAGTTACCGTTCCGCTGCAAAGCGGTGCAACTGCACCGCAACTGGCAGCAATTCTGGCAGAAACCACCCCTCGTGTTCTGGCAGCAACTCCTGATCACCTGGACATTGCAGTAGAGCTACTGACCGGAGGGGCAAGCCCGGAGCGTCTGGTTGTCTTTGACTATCGTCCTGCAGATGATGACCATCGTGCAGCATTAGAAAGCGCACGTAGGCGTCTAAGCGATGCAGGTTCGGCAGTTGTTGTTGAAACCTTAGATGCAGTCCGTGCACGCGGTAGCGAGCTACCGGCAGCACCGCTGTTTGTCCCTGCAGCAGATGAAGATCCGCTGGCTCTGTTAATTTACACCAGCGGTAGCACCGGGACCCCCAAAGGTGCAATGTATACCGAGAGGCTGAATCGTACTACGTGGCTGAGCGGTGCAAAGGGTGTTGGTTTAACCTTAGGTTATATGCCGATGTCGCACATAGCAGGACGTGCATCATTTGCAGGGGTTCTGGCACGCGGTGGTACCGTTTATTTTACCGCACGCAGCGACATGAGCACCCTGTTTGAGGACCTGGCACTGGTTCGTCCGACCGAAATGTTTTTTGTTCCGCGTGTTTGTGACATGATTTTTCAACGTTACCAGGCAGAGCTGAGCCGGCGTGCACCTGCAGCAGCAGCAAGCCCGGAGTTAGAACAGGAGCTGAAAACCGAGCTGCGTCTAAGCGCAGTCGGAGATCGTCTATTAGGGGCAATTGCAGGTAGCGCACCGCTGAGCGCAGAAATGCGTGAATTTATGGAAAGCCTGCTGGACCTGGAGCTGCATGACGGTTATGGTAGCACCGAAGCAGGGATTGGTGTCCTGCAAGATAACATTGTTCAGAGGCCGCCGGTTATTGACTATAAATTAGTTGATGTTCCGGAGCTAGGTTATTTTCGTACGGATCAGCCGCACCCTCGTGGGGAACTACTGCTAAAGACCGAGGGAATGATACCGGGTTATTTTCGTCGTCCTGAAGTTACCGCAGAAATTTTTGACGAAGATGGGTTTTACAGGACCGGGGATATTGTTGCAGAGTTAGAGCCGGACCGTCTGATTTACCTTGATCGTCGTAATAACGTTCTGAAGCTGGCACAGGGTGAATTTGTTACCGTTGCACACCTGGAGGCAGTTTTTGCAACCTCGCCGCTGATTCGTCAGATTTACATTTACGGTAATAGCGAACGTAGCTTTCTGCTGGCAGTTATTGTTCCTACCGCAGATGCACTGGCAGATGGGGTTACCGATGCACTGAATACCGCACTGACCGAGAGCCTAAGGCAGTTAGCAAAGGAGGCAGGACTGCAAAGCTACGAACTGCCGCGTGAATTTCTGGTTGAGACCGAGCCGTTTACCGTAGAAAATGGGCTGTTAAGCGGGATTGCAAAGCTGCTACGTCCTAAATTAAAAGAACATTACGGTGAAAGGTTAGAACAGCTGTATCGTGACATTGAGGCAAATCGTAATGATGAACTGATTGAGCTGCGTCGTACCGCAGCAGAACTGCCGGTTTTAGAGACCGTAACCCGTGCTGCTAGGAGCATGTTAGGGCTGGCAGCAAGCGAACTACGTCCGGATGCACACTTTACCGACTTAGGTGGGGACTCGCTGAGCGCACTGAGCTTCAGCACCCTGCTGCAGGATATGTTAGAAGTTGAAGTTCCGGTTGGGGTTATTGTTAGCCCTGCAAATAGCTTAGCAGACCTGGCAAAGTATATTGAGGCAGAGCGTCACAGCGGAGTTCGTCGTCCGAGCCTGATTAGCGTTCATGGGCCTGGTACCGAAATTAGGGCAGCAGACTTAACCCTGGATAAATTTATTGACGAACGTACCTTAGCTGCAGCAAAGGCAGTCCCGGCAGCACCGGCACAGGCACAGACCGTACTGTTAACCGGAGCAAATGGTTACCTGGGTCGTTTTCTGTGTCTGGAGTGGCTGCAGAGGCTGGATCAGACCGGGGGGACCCTGGTTTGTATTGTTCGTGGGACCGATGCAGCAGCAGCACGCAAACGTCTGGACGCAGTTTTTGACAGCGGGGACCCGGAGCTGTTAGATCATTACCGTAAACTGGCAGCAGAACATTTAGAAGTTTTAGCAGGTGACATTGGTGATCCGAACTTAGGTCTGGATGAGGCAACTTGGCAGCGTTTAGCAGCAACCGTCGATCTGATTGTTCATCCTGCAGCATTAGTTAATCACGTTCTGCCGTATAGCCAGCTGTTTGGACCGAACGTTGTTGGTACCGCAGAAATTATTCGTCTGGCAATTACCGAACGTAGGAAACCTGTTACCTATCTGAGCACCGTTGCAGTTGCAGCTCAGGTTGACCCGGCAGGTTTTGACGAAGAACGTGACATTCGTGAAATGAGCGCAGTTCGTAGCATTGACGCAGGATATGCAAATGGGTATGGTAATAGCAAATGGGCAGGTGAAGTTCTGCTGCGTGAAGCACACGATCTGTGTGGACTGCCGGTTGCAGTTTTTCGTAGCGATATGATTCTGGCACATAGCAAGTATGTTGGGCAGTTAAATGTTCCTGACGTTTTTACCCGTTTAATTCTGAGCCTGGCATTAACCGGGATTGCTCCGTACAGCTTTTACGGAACCGATAGCGCAGGACAGCGTAGGCGTGCACATTACGACGGGCTGCCTGCAGACTTTGTTGCAGAAGCAATTACCACCTTAGGTGCAAGGGCAGAAAGCGGATTTCACACCTATGATGTTTGGAATCCGTATGATGATGGTATTAGCCTGGATGAGTTTGTTGATTGGTTAGGTGACTTTGGTGTTCCGATTCAGCGTATTGACGACTATGATGAGTGGTTTCGTAGGTTTGAAACCGCAATTCGTGCACTGCCTGAGAAACAGCGTGACGCTAGCCTGCTACCGCTGCTGGATGCTCATCGTCGTCCCCTGCGTGCAGTTCGTGGGAGCCTGCTACCTGCAAAAAATTTTCAAGCAGCAGTTCAGAGCGCACGCATTGGTCCTGACCAGGATATTCCGCACCTAAGCCCGCAGCTAATTGACAAATATGTTACCGATCTGCGTCATTTAGGTCTGTTATAA

Ka_ACAR

ATGACCACCGGTAGCTGGTCAGAAACCAGCGAAGTTCATGGTGTTAGCGGTCCGCGTGAAGAACGTCGTGCAGCACAGCTGCGTGCACAGGATGAACAGGTTCGTGCAGCCGCACCGCTGGATGCAGTTAATGAAGCAACCAGCAGTCCGGGTCAGCGTCTGACACAGGTTGTTGCAGCAATTATGGCAGGTTATGCAGATCGTCCGGCACTGGGTGAACGTGCCCGTGAACTGGTTACCGATCCGGGTACAGGTCGTACCAGCATTCGTCTGCTGCCGTGGTTTGATACCATTAGCTATCGTGAACTGTGGACCCGTGTTGGTGCAATTGCCAGCGATTGGCATCATCATCCGGATCATCCGCTGGCAGCCGGTGAATTTGTTGGTATTCTGGGTTTTACCAGCTGTGATTATACCACCCTGGATCTGGTTTGTCTGCATCTGGGTGCAGTTTGTGTTCCGCTGCAGAGCAGCTCACCGGCAAGCCAGCTGCGTCCGATTATTGCAGAAACCGGTCCGAGCATTCTGGCAACCAGCGCAGAACGTCTGGATACCGCAGTTGAACTGGCCCTGGGTAGCCCGACCGTTCGTCGTCTGGTTGTTTTTGATAGCCATCCGGAAGTTGATGAACAGCGTGAAGCACTGGAAAGCGCACGTCAGCGCCTGACCGAAGCAGGTCATCCGGCAGTTGTTGATAGCCTGGCAGCAGTTCTGGAACGTGGTCGTGCACTGCCTCCGGCACCGCTGTTTACACCGGGTCCGGATGAAGATCCGCTGACCATGCTGATTTATACCAGCGGTAGCACCGGCACCCCGAAAGGTGCAATGTATCCGGAACGCCTGGTTCATAGCCTGTGGGATGGTCTGTGGCGTGATAAAAATGCACTGCCGGTTATTGGCATTAACTATATGCCGATGAGCCATCTGGCAGGTCGTATTAGCCTGCTGCGTGCCCTGAGCAGCGGTGGCACCAGCTATTTTGCAGCAAAAAGCGATCTGAGCACCCTGTTTGAAGATATTGCACTGATTCGTCCGACCGAACTGAATCTGGTTCCGCGTGTTTGTGATATGCTGTTTCAGCGTTATCAGAGCGAACTGGATCGTCGTGCACCGGGTACAAGTGATCTGGATGCCGTTGATGCACAGGTTAAACAAGAACTGCGTGAAGGTTTTCTGGGTGGTCGTGTTGTTCGTGCAATGTGCAGTACAGCACCGCTGAGCGCAGAAATGGCAGCATTTGTTGAAAGCTGTCTGGACCTGGAACTGCATGATGGTTATGGTAGTACCGAAGCCGGTGGTGTTGTTATTGATAAACATGTTCTGCGTCCGCCTGTGCTGGATTATAAACTGGTTGATGTGCCGGAACTGGGTTATTTTCGTACCGATACACCGCATCCGCGTGGTGAACTGCTGATTAAAACCCGTACCATTATTCCGGGTTATTTCAAACGTCCGGATGCAACCGCAGAAATTTTTGATGCCGATGGTTATTATCAGACCGGTGATATTATGGCCGAAATTGGTCCGGATCAGCTGGTTTATGTTGATCGTCGTAAAAATGTTCTGAAACTGAGCCAGGGCGAATTTGTGGCAGTTAGCCGTCTGGAAGCAGTTTTTGCAACCAGTCCGCTGGTTCGTCAGGTTTTTGTTTATGGTAGCAGCGCACGTGCATATCTGCTGGCCGTTGTTGTGCCGACCGAAGAAGCACTGCGTCGTACCGTTACCGATAATGCAGCACTGAAAAGCAGCATTAGCGAAAGCCTGCAGCGTATTGCACGTGAAGCAGAACTGAATAGCTATGAAATTCCGCGTGATCTGCTGATCGAAACCGATCCGTTTAGCACCGAAAATGGTCTGCTGAGTGATGCACGTAAACTGCTGCGTCCTCGTCTGAAAGAACATTATGGTGAGCGTCTGGAACAGCTGTATGCAGAACTGGCAAAAGGTCAGGTGGATGAACTGCACGCACTGCGTGTTACCGGTCGTGATCGTCCTGTTCTGGAAACCGTTACCCGTGCAGCCCAGGCACTGCTGGGTTGTGCAAGCACCGATCTGTCACCGGATGCACATTTTACCGAACTGGGTGGTGATAGTCTGAGCGCACTGAGCCTGAGCAATCTGCTGCAAGAAATCTTTACCGTTGAAGTTCCGGTTGGTGTGGTGATTAGTCCGGCAAATGATCTGCGTCAGCTGGCAAATTATGTTGAAACGGAACTGAGCAGTGGTGCAAAACGTCCGACCTTTGCAACCGTTCATGGTCAGGGTAGCCTGGAAGTTCGTGCCGCAGATCTGACCCTGGATAAATTCATTGATAGCGCAACCCTGGCAGGCGCAAAAGATCTGCCTGGTCCGAGCGGCACCGCACGTACCGTTCTGCTGACCGGTGCAAATGGTTATCTGGGTCGTTTTCTGTGTCTGGAATGGCTGCGTCGTCTGAGTCAGGATGGTGGTAAACTGGTGTGTATTGTTCGTGGTAGCAGTGCAGAAGCAGCACGTCGTCGCCTGGAACAGGCATTTGATAGCGGTGATGCAGAGCTGCTGCGCCTGTTTCGCGAACTGGCTGCCGAACATCTGGAAGTTCTGGCAGGCGATATTGGTGAACCGGATCTGGGTCTGGATGAGCAGACCTGGCATCGTCTGGCAGATAGCGTTGATCTGATTGTTCATCCAGCAGCACTGGTTAATCATGTACTGCCGTATCAGCAGCTGTTTGGTCCGAATGTTGTTGGCACCGCAGGTCTGATTCGTATGGCAATTACCAAACGTCTGAAACCGTTTGTTTATCTGAGTACCGTTGGTGTTCTGAGTGCACAGATTGCACCGAGTGCACTGCGCGAAGATCTGGATATTCGTGATACCAGTCCGGTTCGTCGGCTGGATCAGAGCTATGCAAGCGGTTATGGCACCAGTAAATGGGCAGGCGAAGTTCTGCTGCGGGAAGCACATGAAGCATTTGGTCTGCCTGCAGTTGTTTTTCGTAGTGATATGATTCTGGCACATAGCCGTTATACAGGTCAGCTGAATGTTCCTGATATGTTTACACGCCTGCTGCTGAGCCTGGTTCTGACCGGTATTGCTCCGAAAAGTTTTTATCGTACCGGTAGTGATGGTGGTCGTCAGCGTGCACATTATGATGGTCTGCCAGCCGAATTTACCGCAGAAGCAATCACCGAGCTGGGTGCGCGTGCAGCAGCCGGTTATCGCACCTTTAATGTGCTGAACCCGCATGATGATGGTATTAGTCTGGATGTGCTGGTTGATTGGCTGGCGGAAACGGGTCATCCGATTCAGCGCATTGAAGATTATCAAGAATGGTTTGCACGTTTCGATACAGCCCTGCGTGCTCTGCCGGAAAAACAGCGTCAGCACTGCCTGCTGCCGCTGATGCATGCCTTTGAACAGCCTGGTGTTCCGGTGGCAGGTAGCGTGATTCCTGCAGATGAATTTCGTGCGGCAGTTCGTACCGCAAAAATTGGCCCTGATAAAGATATTCCGCATCTGAGCGCCAGCCTGATTACCAAATATGTTCGCGATCTGGAACAACTGGGCCTGGTTTAA

Ms_ACAR

ATGTTTACCGATAGCCGTGAAGATCGTCTGGCACGTCGTGTTGCAGATCTGTATAGCACCGATATTCAGTTTGTTGATGCACGTCCGATTGAAGCACTGAGCGCAGCAATTGAACAGCCTGGTCTGCGTCTGCCTCGTATTATGAGCACCGTTATGGGTGCATATGCCGAACGTCCGGCAGTTGGTGAACGTGGTGTTAATCTGACCACCGATCCGGCAACCGGTCGTACCAGCCTGGAACTGCTGCCGTGTTTTCAGACCATTACCTATCGTGAACTGTGGGATCGTGTTGGTGCAGTTGCAAGCGGTCTGACCCAGGGTCCGGGTCCGGTTCAGCCTGGTGATCGTGCATGTGTTCTGGGTTTTGCAAGCGCAGATTATGCAACCATTGATATGGCACTGGTTCTGCTGGGTGCAGTTAGCGTTCCGCTGCAGACCCGTGCACCGGTTAGCCAGCTGCGTCCGATCGTTACCGAAACCGAACCGCGTGTTTTTGGTAGCAGCATTGGTGATCTGGCAAATGCAGTTGAACTGGTGCTGACCGGTTATACACCGGCACGTCTGGTTGTTTTTGATTATCATCCTGATGTTGATGATCAGCGCGAAGCATTTGATGCAGCAAAAACCCGTCTGGCAGAAGCAGGTAGTCCGGTTCTGGTTGAAACCCTGGCAGCACTGCTGGATCGTGGTGCAAGCCTGCCTGCAGCACCGGAATTTGTTCCGGATGATGATGAAGATCCGCTGACCCTGCTGATTTATACCAGCGGTAGCACAGGTGCACCGAAAGGTGCAATGTATCCGGAACGCCTGGTTGCAAATTTTTGGCGTCGTAGCCGTTGGAATTGGGGTAGCAGCGTTGAACCGCTGATTACCCTGAGCTTTATGCCGATGAGCCATGTTATGGGTCGTGGTATTCTGTATGGTACACTGGGTCAGGGTGGCACCGCATATTTTACCGCACGTAGCGATCTGAGCACCCTGTTTGAAGATCTGGCCCTGGTTCGTCCGACCGAACTGAATTTTGTTCCGCGTATTTGGGATATGCTGTTCACCGAATTTCAGAGCGAAGTTGATCGTCGTAGCGTGGATGGTGTTGATCGTGCCGTTCTGGAAGCAGATGTTATGGCAGAACAGGCACAGAATCTGCTGGGAGGTCGTTTTGTTAGCGCAATGACCGGTAGCGCACCGATTAGCGCAGAAAATAAAGAATTTGTTGAAGCCCTGCTGGACCTGCATCTGGTGGAAGGTTATGGTAGCACCGAAGCCGGTATTATCTATATTGATGGTCAGGTTCGTCGTCCTGCCGTGATTGATTATAAACTGGTTGATGTGCCGGATCTGGGTTATTTTCATACAGATCAGCCGTTTCCGCGTGGTGAACTGCTGGTTAAAACCCAGGACCTGTTTCCGGGTTATTACAAACGTCCGGAAGTGACCGCAGATGTGTTTGATCCGGATGGTTATTATCGTACCGGTGATGTTGTTGCAGAAGTGGATCCTGATCAGCTGGTTTATCTGGATCGTCGCAATAATGTTCTGAAACTGAGCCAGGGTGAATTTGTTACCGTTAGCAAACTGGAAGCCGTTTTTGGTGATAGTCCGCTGGTGCGTCAGATTTATGTTTATGGTAATAGCGCACGCAGCTATGTTCTGGCAGTTGTTGTTCCGACCGATGATACCCTGAATCGCACCGGTGGTGATGTGGAAAGCCTGAAAAGCGCAATTAGCGAAAGCCTGCAGAATATTGCAAAAGATGTTGGTCTGCAGAGCTATGAAATTCCGCGTGATTTTATCATTGAAACCACCCCGTTTACCCTGGAAAATGGTCTGCTGACAGGTATTCGTAAACTGGCTCGTCCGAAACTGAAAGTTCATTATGGTGATCGGCTGGAACAGCTGTATACCGAACTGGCAGATAGCCAGGCAAATGAACTGCGTGCACTGCGTCAGAATGGTGCAGATCGTCCTGTGCTGGAAACCGTTATTCGTGCAGCCGGTGCAGTTCTGGGTGCCGCAGCAAGCGATCTGGAACCGGATGCACATTTTAGTGATCTGGGTGGTGATAGCCTGAGCGCACTGACCTTTGGTAATCTGCTGCGTGAAATTTTTGATATCGATGTTCCGGTTGGTGTTATTGTTAGTCCGGCAAGTGATCTGCGTAGCATTGCAGGTTATATTGAAGCAGAACGTCGTCCGGGTGCAAAACGTCCGACCTTTGCCAGCGTTCATGATCGTAGTAATAAACCGATTACCGAAGTTCTGGCCAGTGATCTGACCCTGGATAAATTCATTGATGCAAAAACTCTGGCAGCCGCAAGCACCCTGCCGAGCCCGAGCGCAGAAGTTCGTACCGTTCTGCTGACGGGTGCAACCGGTTTTCTGGGTCGTTATCTGGCACTGGAATGGCTGGAACGTATGAATCTGGTTGGTGGTAAACTGATTTGTCTGGTTCGTGCCAAAGATGATGTTGCAGCACGTGACCGTCTGGATAAAACCTTTGATAGCGGTGATCCTGAGCTGCTGCGTCATTATCGTGAGCTGGCAGCAGGTCATCTGGAAGTTATTGCCGGTGATAAAGGTGAAGAAAATCTGGGTCTGGATCAGCAGACATGGCAGCGTCTGGCCGATATTGTTGATCTGATTGTGGATCCGGCAGCCCTGGTTAATCATGTTCTGCCGTATAGCGAACTGTTTGGTCCGAATGCACTGGGTACAGCAGAACTGATTCGTATTGCACTGACCACCAAACAGAAACCGTATACCTATGTTAGTACCATTGGTGTGGGTGATCAGATTGAACCGAGCAGCTTTACCGAAGATGCAGATGTTCGTGTTATGAGCCCGACCCGTGCCATTAATGAAGGCTATGCAAATGGTTATGGCAACAGCAAATGGGCTGGTGAAGTGCTGCTGCGCGAAGCCAATGATCTGTGTGGTCTGCCGGTTGCAGTTTTTCGTTGTGATATGATTCTGGCGGATACCGCATATGCAGGTCAGCTGAATGTTCCTGATATGTTTACCCGTCTGCTGCTGAGCGTTGTTGCCACCGGTATTGGTCCGGGTAGCTTTTATGAACTGGATGCCGATGGTAATCGTCAGCGTGCCCATTTTGATGGCCTGCCGGTGGGTTTTATTGCAGATGCAATTAGCACCCTGGGTGCACAGGTTGTTGATGGTTATGAAACCTATCATGTGATGAACCCGTATGATGATGGTATTGGCCTGGATGAATATGTTGATTGGCTGACCGATGCCGGTTATCCGATTCAGCGTATTAGCGGTTATGATGCATGGCTGCAGCGTTTTGATACCGCACTGCGTGCCCTGCCGGATAAACAGCGTCAGGCCAGCCTGCTGCCGCTGCTGCATAACTATCAGCGTCCGGAAAAACCGATTCGTGGTAGCATGGCACCGACCGATCGTTTTCGTGCGGCAGTTCAGGATGCCAAAATCGGTCCGGATAAAGATATTCCGCATGTTACAGCACCGATGATCGTGAAATATATCACCGATCTGCAGCTGCTGGGTCTGCTGTAA

Rw_ACAR

ATGAGCACCGATATTCGTGAAGAACGTCTGGCACGTCGTATTGCAGATCTGTATGCAAATGATCGTCAGTTTGTTGCAGCACGTCCGAGCGAAGCACTGACCGCAGCAATTGAACAGCCTGGTCTGCGTCTGCCGCAGCTGGTTCGTACCGTTATGGAAGGTTATGCAGATCGTCCGGCACTGGGTCAGCGTGCAGTTCAGTTTATCAAAGATCCGGCAACCGGTCGTACCTTTCCGGAACTGCTGCCTCGTTTTGATACCATTACCTATCATGAACTGTGGGATCGTGTTGGTGCAGTTGCAAGCGCACTGGCAGGCGGTCGTAGCCCGAGTGTTCGTCCGGGTGATCGTGTTTGTATTCTGGGTTTTACCAGCGTTGATTATACCACCATTGATATGGCACTGGTTCAGATGGGTGCAGTTTGTGTTCCGCTGCAGAGCAGCGCACCGTTTACCCAGCTGCGTCCGATTGTTGCAGAAACCGAACCGCGTATGATTGCAAGCAGCATTGATTATCTGGCAGATGCAGTTGAACTGGTTCTGACAGGTCATGCACATGCACGTCTGGTTGTTTTTGATTATCATCCGGAAGTGGATGATCAGATCGAAGCATATGATGCAGCCCGTGCCCGTCTGACCGAAGCAGGTAGTCCGGCAGTTCTGGAACGCCTGACCGATGTGCTGGAACGTGGTCAGACCCTGCCTGCAGCACCGGTTTTTATCCCGGATGATCCGCTGACCCTGCTGATTTATACCAGCGGTAGCACAGGTGCACCGAAAGGTGCAATGTATCCTGAACGCCTGGTTGCAAATTTTTGGCGTCGTAGCACCCGTGCAAGCTGGGGACAGCAGGGTGCAGAACCGAGCATTACCCTGAGCTTTCTGCCGATGAGCCATGCAATGGGTCGTGGTATTCTGTATGGCACCCTGGGTAATGGTGGCACCGCATATTTTGCAGCAAAAAGCGATCTGAGCACCTTTCTGGATGATCTGGCCCTGGTTCGTCCGACCCAGCTGACCTTTGTTCCGCGTATTTGGGATATGCTGCTGCAAGAATTTCGTAGCGAAGTTGATCGTCGTAGCTCAGATGGCACCGATCGTGGTGCACTGGAAGCAGAAGTTATGGCAGAACAGCGTCAGACACTGCTGGGTGGTCGTTTTGTTAGCGCACTGAGTAGCAGTGCACCGATTAGCGCAGAAACCAAAGCATTTGTTGAATATTGTCTGGATCTGCGCCTGGTGGAAGGCTATGGTAGCACCGAAGCCGGTTCAGTTTTTGTTGATGGTGTTGTTCGTCGTCCGCCTGTGATTGATTATAAACTGGCAGATACACCGGGTCTGGGTTATTTTCATACCGATCAGCCGCATCCGCGTGGTGAACTGCTGGTGCGTAGTGATGATGTTTTTCCGGGTTATTACAAACGTCCGGAAGTTACAGCCGAAGTTTTTGATGTGGATGGTTATTATCGTACCGGTGATATTGTTGCCGAAATTGCACCGGATCAGCTGGTTTATCTGGATCGTCGCAATAATGTTCTGAAACTGAGCCAGGGTGAATTTGTTGCCGTTAGCAAACTGGAAGCCGTTTTTAGCAGCAGTCCGCTGGTTCGCCAGATTTATGTTTATGGTAATAGCGCACGTGCATATCTGCTGGCAGTTGTTGTTCCGACCGAAAGTGCACTGAGCCGTGGTAGCGGTGATCTGGAAAGCCTGCAGCCGCTGATTAGCGATAGCCTGCAGGATGTTGCACGTACCGCAGGTCTGCAGAGCTATGAAATTCCGCGTGGCTTTATTGTTGAAGCAAGCCCGTTTACACTGGAAAATGGTCTGCTGACCGGTATTCGCAAACTGGCTCGTCCGAGCCTGAAAGAACGTTATGGTCAGCGTCTGGAACAAATGTATACCGCACTGGCCGAAGGTCAGACCGATGAACTGCGTGCACTGCGTCGTAGTGGTGCCGATCGTCCTGTTCTGGAAACCGTTACCCGTGCAGCGGGTGCCCTGCTGAGCGCAGCAGCAGTTGATCTGCAGCCGGATGCACATTTTACCGATCTGGGTGGTGATAGCCTGTCTGCACTGACATTTGCCAATCTGCTGCGTGAAATTTTTGATATTGATGTTCCGGTTGGCGTTATTGTTAGCCCTGCAACCGATCTGCGTGTTATTGCCGATTATATTGAAGCAAAACGTACCAGCAGCACAAAACGTCCGACCTTTGCAACCGTTCATGGTCGTGATGCCACCGAGCTGCGTGCAAGTGATCTGACACTGGATAAATTTCTGGACGGCACCACCCGTGCGGCAGCACCGACACTGCCTGGTCCGAGTGCAGAAATTCGCACCGTTCTGCTGACAGGTGCAACCGGTTTTCTGGGTCGTTATCTGGCGCTGGAATGGCTGCAGCGCATGGAAGGTGTTGGTGGTACACTGATTTGTCTGGTTCGTGCAAAAGATGATGCCGCAGCACGTAGCCGTCTGGATACCATTTTTGATGGTGGTGATCCTGACCTGCTGCGTCATTATCGTGAACTGGCAGCCGATCATCTGGAAGTTATTGTTGGTGATATGAGCGAAACCAATCTGGGTCTGGATAAACAGACCTGGCAGCGCCTGGCCGATACCGTTGATCTGATTGTTGATCCGGCAGCACTGGTTAATCATGTTCTGCCGTATTGTCAGCTGTTTGGTCCGAATGTTCTGGGCACCGCAGAACTGATTCGTATTGCCCTGACCACCAAACGTAAACCGTTTGCATATGTTAGCACCATTGGTGTGGGTGCACAGATTGAACCGGCAACCTTTGCCGAAGATGCAGATATTCGCGTTGTGAGCCCGACCCGTACCGTGGATGATAGCTATGCCAATGGTTATAGCAATAGCAAATGGGCAGGCGAAGTTCTGCTGCGCGAAGCACATGATCTGTGTGGTCTGCCGGTTGCGGTTTTTCGTTGTGATATGATTCTGGTGGATACCGAATATGCCGGTCAGCTGAATCTGCCGGATATGTTTACCCGTATGATGCTGAGCCTGGTGGCAACAGGTATCGCACCGGGTAGCTTTTATGAACTGGATGCAAATGGTAATCGCCAGCGTGCACATTATGATGGCCTGCCGGTGGATTTTATTGCAGAAGCAATGGCAACCCTGGCAGAAAAAGTTACCGATGGTTTTGAAACGTATCATGTGATGAATCCGTATGACGATGGTATTGGCCTGGATGATTATGTTGATTGGCTGATTGATGCTGGCTATAGCATTCAGCGTATTCCGGATTATGGTGCATGGCTGCAACGCTTTGAAACCGCAATGCGTGCCCTGCCGGAACAGCAGCGTCAGCATAGTCTGCTGCCACTGCTGCATAACTATCAGCGTCCTGAAAAACCTGTTTGTGGTAGTATTGCACCGACCGAACGTTTTCGTGCAGCCGTGCATGATGCAAAAATTGGTCCGGATAAAGATATTCCGCATGTTGTTGCTCCGATCATCGTGAAATACATTACCAATCTGCAGCTGCTGGGTCTGCTGTAA

Ss1_ACAR

ATGGCAGAACTGCGTGCACATGATCCGCAGGTTCGTGATGCAATGCCGCTGCCTGCAGTTGATGCAGCAATTGGTCGTCCGGGTCTGACCCTGAAAAAAATCATTGCAACCGCAATGGAAGGTTATGCAGATCGTCCGGCACTGGGTGAACGTGCCCGTGAACTGATTCGTGATCCGGCAACCGGTCGTGCAGAATATCGTCTGCTGCCGCATTTTGATACCATTACCTATGCCCAGCTGTGGTCACGTGTTGAAGCACTGGCAGCTGATCTGCATCATGATCCTCGTCAGCCGCTGCGTGCCGATGAATTTATTGCAGTTCTGGGTTTTACCAGCACCGATTTTGTGACCATTGATCTGACCTGTGCACGTCTGGGTGCAGTTTGTGTTCCGCTGCAGAGCAGCGCAAGCGCAGCCCGTCTGGGTCCGGTTATTGCAGAAACCGGTCCGCGTATTCTGGCAGCCGGTGTTGAATTTCTGGATACCGCAGTTGATTGTGCACTGGATAGCGGTAGCGTTGGTCGTCTGATTGTTTTTGATCATCGTCCGGAAGCAGATGATGAACGTGAACGTTTTGAAGCAGCACGTCAGCGTCTGACCGATGCAGGTAGCACCGTTGTTCTGGAAAGCCTGGCAGATGTTCTGGATCGTGGTCGTGGTCTGCCTCCGGCACCGGAACTGGAAGATGGCACCGATACCGGTCGTCTGGCACTGCTGCTGTATACCAGCGGTAGTACCGGCACCCCGAAAGGTGCAATGTATACCGAACGTCTGGTTGGTCGTATGTGGCATGGTTTTTGGCCTGGTAAAAGCAGCCTGCCGCTGATTATGCTGAGCTATATGCCGATGAGCCATCTGGCAGGTCGTGCAACCCTGTATACCGTTCTGGGTAGCGGTGGCACCGTTTGTTTTACCGCACGTAGCGATCTGAGCACCCTGTTTGAAGATCTGGGTCTGGTTCGTCCGACCGATCTGCTGCTGGTTCCGCGTGTTTGTGATATGCTGCTGCAACATTATCGTGGTGAACTGGATCGTCGTACCGCAGCGGGTGGCGATCCGGCAGTGCTGGAAGCCGAAGTTAAACGTGATCTGGGCGAACGTAGCCTGGGTGGTCGCCTGCTGTGGATTGGTAGTGGTGGTGCACCGCTGAGTGATGAAATGACAGATTTTGTTGCAAGCTGTGTTGATGTGCCGCTGCATGATGGTTATGGTTCAACCGAAGCCGGTGGTATGCTGGCAGATCACCGTCCGATCCGTCCGGCAGTTCGTGATTATCGTCTGGTGGATGTTCCTGAACTGGGTTATTTTCGTAGTGATCATCCGCATCCGCGTGGCGAACTGCTGATTCGTACCGATGCACTGATTCCGGGTTATTACAAACGTCCGGATGTTATGGCAGGTCTGGTTGATGAAGATGGTTATTATCGTACCGGTGATATTTTTGCAGAACGTGGTCCGGATGAACTGTTTTATGTTGATCGTCGCAATAACGTTCTGAAACTGAGCCAGGGTGAATTTGTTGCCGTTAGCCGTCTGGAAGCAGTTTTTGCAGGTAGTCCGCTGGTGCGTCAGATTTTTGTTTATGGTAGCAGCGAACGTGCATATCTGCTGGCAGTTGTTGTTCCGGTTCCTGAAGCTGCCGAACGTGCCGGTGGTGATCCTGCAGAACTGAAAGCACGTATTGCAGCAAGCCTGCGTGGCACCGCAAAAGAAGCAGGTCTGAATAGCTATGAAATTCCGCGTGATTTTCTGATTGAAACCGAACCGTTTAGCACCGAAAATGGTCTGCTGAGCGATATTCGTAAACTGCTGCGTCCTCGCCTGACCGAACGTTATGGTGAACGCCTGGAACGTCTGTATGCCGATCTGGCAGCACGTGAAAATGATGAGCTGCGTGCGCTGCGTCGTAGCGGTCGTGATCGTCCTGTTGCAGATACCGTTGTGCGTGCAGCACAGGCGGTGCTGGGTACACCGGCAGATCCTGCCGCACGTTATACGGATCTGGGTGGTGATAGCCTGAGCGCAGTTAGCTTTAGCCAGCTGCTGGGTGAAATTTTTGGTGTTGAAGTTCCGGTTGGTGTTGTTATTAGTCCTGCCCATGATCTGCGTCGCCTGGCGGAACATGTTGAACGTGCACTGAGCAGCGGTGATCGTCGTCCGAGTCCGGCTACCGTTCATGGTGCCGGTGCAACCGAAGTTCGTGCACGCGATCTGGCCCTGGATGCATTTGTTGATGCCGAAACCCTGGCAGCAGCACCGAGTCTGCCGCATGTTGCAGGTCCGGCACGTACCGTGCTGCTGACCGGTGCAAATGGTTATCTGGGTCGTTTTCTGTGTCTGGAATGGCTGGAACGGCTGGCACGCACCGGTGGTACACTGGTTTGTGTTGTTCGTGGTTCAGATGCAGCAGTTGCACGTCGTCGCCTGTATGAAGCATTTGATTCAGGTGATCCGGAACTGCTGGCTCGTTTTGGTGAGCTGGCAGAAGGTCGCCTGGAAGTTCTGGCAGGCGATATTGGCGAACCGGACCTGGGCCTGGATGGTCCGACCTGGAATCGCCTGGCCGATACCGTGGATCTGATTGTACATCCGGCAGCACTGGTTAATCATGTTCTGCCGTATGAGCAGCTGTTTGGTCCGAATGTTGTTGGTACAGCCGAACTGATCCGTCTGGCGCTGACCCGTCGTGTTAAACGTTTTACCTATCTGAGTACCGTTGGTGTTATTGCCGCACAGGCAGCAACCGCAGATGAAAGCGCAGATATTCGTGTTGCATCACCGGTTCGTCGTCTGGATGATAGCTATGCAAGCGGTTATGCAACCAGCAAATGGGCAGGCGAAGTTCTGCTGCGTGAAGCGCATGATCTGTGCGGTCTGCCGGTTGCCACCTTTCGTAGCGATATGATTCTGGCACATAGCCGCTATGGTGGTCAGCTGAATGTTCCGGATGTGTTTACACGCCTGCTGCTGAGTCTGGCTGCAACCGGTATTGCACCGGGTAGCTTTTATCGTGCCGGTCCGGGTGGTGTGCGTCGTCCTGCACATTATGAAGGCCTGCCGGTGGATTTTACAGCAGAAGCAGTTACCGCACTGGGCGAGCGTGCGACCGAAGGTCATCGTACCTATAATGTTCTGAATCCGCATGATGATGGTGTTAGCCTGGATGTTTTTGTAGATTGGCTGGTAGATGCAGGTCATCCGGTGCGTCGTATTGATGATTATGATGAATGGCTGGCACGTTTCGGTACAGCAATGCGTGCACTGCCGCAAGAACAGCGTCAGCATAGCCTGCTGCCTCTGCTGCATGCATTTGCCGAACCAGCCGAACCGGTTGCGGGTAGTGCCGTTCCGGCAGATGCATTTCGTGCAGCGGTTCTGGAAGCGGGTGTGGGTCCTGATGCCGATATTCCGCATCTGAGCGCAAGCCTGATTGGTAAATATGCAGCCGATCTGCGTGCCCTGAAACTGATCTAA

Mm_ACAR

ATGAGCCCGATTACCCGTGAAGAACGTCTGGAACGTCGTATTCAGGATCTGTATGCAAATGATCCGCAGTTTGCAGCAGCAAAACCGGCAACCGCAATTACCGCAGCAATTGAACGTCCGGGTCTGCCGCTGCCGCAGATTATTGAAACCGTTATGACCGGTTATGCAGATCGTCCGGCACTGGCACAGCGTAGCGTTGAATTTGTTACCGATGCAGGTACAGGTCATACCACCCTGCGTCTGCTGCCGCATTTTGAAACAATTAGCTATGGTGAACTGTGGGATCGTATTAGCGCACTGGCAGATGTTCTGAGCACCGAACAGACCGTTAAACCGGGTGATCGTGTTTGTCTGCTGGGTTTTAATAGCGTTGATTATGCCACCATTGATATGACCCTGGCACGTCTGGGTGCAGTTGCAGTTCCGCTGCAGACCAGCGCAGCCATTACCCAGCTGCAGCCGATTGTTGCAGAAACCCAGCCGACCATGATTGCAGCAAGCGTTGATGCACTGGCCGATGCAACCGAACTGGCACTGAGCGGTCAGACCGCAACCCGTGTTCTGGTTTTTGATCATCATCGTCAGGTTGATGCCCATCGTGCAGCAGTTGAAAGCGCACGTGAACGCCTGGCAGGTAGCGCAGTTGTTGAAACCCTGGCCGAAGCAATTGCACGTGGTGATGTTCCGCGTGGTGCAAGCGCAGGTAGTGCACCGGGTACAGATGTTAGTGATGATAGCCTGGCACTGCTGATTTATACCAGCGGTAGCACAGGTGCACCGAAAGGTGCAATGTATCCGCGTCGTAATGTTGCAACCTTTTGGCGTAAACGTACCTGGTTTGAAGGTGGTTATGAACCGAGCATTACCCTGAACTTTATGCCGATGAGCCATGTTATGGGTCGTCAGATTCTGTATGGCACCCTGTGTAATGGTGGTACAGCATATTTTGTTGCAAAAAGCGATCTGAGCACGCTGTTTGAAGATCTGGCACTGGTTCGTCCGACCGAACTGACCTTTGTTCCTCGTGTTTGGGATATGGTGTTTGATGAATTTCAGAGCGAAGTTGATCGTCGTCTGGTGGATGGTGCCGATCGTGTTGCACTGGAAGCACAGGTTAAAGCAGAAATTCGTAATGATGTTCTGGGTGGTCGTTATACCTCAGCACTGACCGGTAGCGCACCGATTTCAGATGAAATGAAAGCATGGGTTGAAGAACTGCTGGATATGCATCTGGTTGAAGGTTATGGTAGCACCGAAGCAGGTATGATTCTGATTGATGGTGCAATTCGCCGTCCGGCAGTTCTGGATTATAAACTGGTTGATGTGCCGGATCTGGGTTATTTTCTGACCGATCGTCCGCATCCGCGTGGCGAGCTGCTGGTTAAAACCGATAGCCTGTTTCCGGGTTATTATCAGCGTGCCGAAGTTACCGCAGATGTTTTTGATGCAGATGGTTTTTATCGCACCGGTGATATTATGGCAGAAGTTGGTCCGGAACAGTTTGTTTATCTGGATCGTCGCAATAATGTTCTGAAACTGAGCCAGGGTGAATTTGTGACAGTTAGCAAACTGGAAGCCGTTTTTGGTGATAGTCCGCTGGTTCGCCAGATTTATATCTATGGTAATTCAGCACGTGCATATCTGCTGGCAGTTATTGTTCCGACCCAAGAGGCACTGGATGCCGTTCCGGTGGAAGAACTGAAAGCACGCCTGGGTGATTCACTGCAAGAAGTTGCAAAAGCAGCAGGTCTGCAGAGCTATGAAATTCCGCGTGATTTTATCATTGAAACCACCCCGTGGACCCTGGAAAATGGTCTGCTGACCGGCATTCGTAAACTGGCTCGTCCGCAGCTGAAAAAACATTATGGTGAGCTGCTGGAACAAATCTATACCGATCTGGCCCATGGTCAGGCAGATGAACTGCGTAGCCTGCGTCAGAGCGGTGCAGATGCACCGGTTCTGGTTACCGTTTGTCGTGCAGCCGCAGCACTGCTGGGTGGTAGCGCAAGTGATGTTCAGCCGGATGCACATTTTACAGATCTGGGTGGCGATAGTCTGAGCGCACTGAGCTTTACCAATCTGCTGCATGAAATCTTTGATATTGAAGTTCCGGTTGGCGTTATTGTGAGTCCGGCTAATGATCTGCAGGCCCTGGCAGATTATGTTGAAGCAGCACGCAAACCGGGTAGCAGCCGTCCGACCTTTGCAAGCGTTCATGGTGCCAGCAATGGTCAGGTTACCGAAGTTCATGCCGGTGATCTGAGCCTGGATAAATTCATTGATGCAGCAACACTGGCGGAAGCACCGCGTCTGCCTGCAGCAAATACCCAGGTTCGTACCGTTCTGCTGACAGGTGCAACCGGTTTTCTGGGTCGTTATCTGGCCCTGGAATGGCTGGAACGTATGGATCTGGTAGATGGTAAACTGATTTGTCTGGTTCGTGCCAAAAGCGATACCGAAGCACGTGCCCGTCTGGATAAAACCTTTGATAGCGGTGATCCGGAACTGCTGGCACATTATCGTGCACTGGCAGGCGATCATCTGGAAGTTCTGGCTGGTGATAAAGGTGAAGCCGATCTGGGTCTGGATCGCCAGACCTGGCAGCGCCTGGCCGATACCGTTGATCTGATTGTTGATCCGGCAGCACTGGTTAATCATGTTCTGCCGTATAGCCAGCTGTTTGGTCCGAATGCACTGGGCACTGCCGAACTGCTGCGTCTGGCGCTGACCAGCAAAATCAAACCGTATTCATATACCAGCACCATTGGTGTTGCAGATCAGATTCCGCCTAGCGCATTTACCGAAGATGCAGATATTCGTGTTATTAGCGCCACCCGTGCAGTGGATGATAGCTATGCCAATGGTTATAGCAATAGCAAATGGGCAGGCGAAGTGCTGCTGCGTGAAGCACATGATCTGTGTGGTCTGCCGGTTGCAGTTTTTCGTTGTGACATGATTCTGGCAGATACCACCTGGGCAGGTCAGCTGAATGTTCCGGATATGTTTACCCGTATGATCCTGAGTCTGGCAGCAACCGGTATTGCTCCGGGTAGCTTTTATGAACTGGCAGCCGATGGTGCACGTCAGCGTGCACATTATGATGGCCTGCCGGTGGAATTTATTGCAGAAGCCATTAGTACCCTGGGTGCACAGAGCCAGGATGGTTTTCATACCTATCATGTTATGAATCCGTATGATGATGGTATTGGCCTGGATGAATTTGTCGATTGGCTGAATGAAAGCGGTTGTCCGATTCAGCGTATTGCCGATTACGGTGATTGGCTGCAGCGCTTTGAAACCGCACTGCGTGCACTGCCTGATCGTCAGCGTCATAGCAGCCTGCTGCCACTGCTGCATAATTATCGTCAGCCGGAACGTCCTGTTCGTGGTAGCATTGCACCGACCGATCGCTTTCGTGCCGCAGTTCAAGAAGCAAAAATCGGTCCGGATAAAGATATTCCGCATGTTGGTGCTCCGATCATTGTTAAATATGTTAGCGATCTGCGCCTGCTGGGTCTGCTGTAA

Fs_ACAR

ATGGCACCGCTGAGTGGTGGTCCGGGTCTGGGTAGTCCGGGTCCGAGTGGTCCGGAACCGTCAGGTCCTGGTCCGTCATCACCGGGTCCGGGTTCAGCAGGTCCGGGTGATCCTGGTCCTGGTGCACCTGGTCTGGATGCCCGTAGCCGTCGTCGTGTTGCAGAACTGTATGCAACCGATCAGCAGGTTCGTGATGCACGTCCGCTGGCAGCAGTTACCGAAGCACTGCGTGCCCCAGGTCTGAGCCTGGGTCGTATTCTGGCCATTATTATGGCAGGTTATGCAGATCGTCCGGCACTGGGTGAACGTGCCCGTGAACTGGTTGTTGATAGCGAAACCGGTGTTCGTACCCTGCGTCTGCTGCCTCGTTTTGATACCATTAGCTATCGTGAACTGTGGTCACGTGCCGGTGCAATTGCCGCAGAATGGCTGCATCATCCGGGTGCACCGCTGGGTGTGGGTGATCTGGTTGGTGTTCTGGGTTTTACCGGTGCAGATTATACCACCATTGAACTGGCATGTGTTCGTGGTGGTGCAGTTAGCGTTCCGCTGCAGAGCGGTGCAGCAGCAAGCCAGCTGGTTAGCATTCTGGCAGAAACCCAGCCTCGTATCCTGGCCGTTACCGTTGATCTGCTGGCAACCGCAGTTGAAGGTGCACTGGCAAGCCCGAGCGTTCGTCGTCTGGTTGTTTTTGATGATCATGCCGAAGCAGATGCACATCGTCAGGCATGTGATGCAGCACGTCGTCGCCTGGCAGATGCAGCCCGTCCGACCGAAAGCGGTCATCCGGCAGTTCTGGATAGCCTGGCAACCCTGGTTGCACGTGGTGCACGTCTGCCTGCACCGCCTGTTCCGGAAGCAGCACCTGATGATGCCCGTCTGGCCACCGTGATTTATACCAGCGGTAGTACCGGCACCCCGAAAGGTGCAATGTATCCGAATGGTCTGGTTAAACTGGCCTGGCGTGCAGGTTTTTGGCCTCGTTCAGATGATCTGCCGGTTATTAGCTATAATTGTCTGCCGATGAGCCATGTTAGCGCACGTGCCACCCTGGCAGGTACACTGGCAGCCGGTGGCACCAGCCATTTTGCAGCAGCCAGCGATCTGAGCACCCTGTTTGAAGATATTGCACTGGTTCGTCCGACAGCGCTGCGTCTGGTTCCGCGTCTGTGCGATCTGCTGTTTCAGCGTCATCGTGGTGAACTGGATCGTCGTTCAGCCGGTGCTGGTGATCAGGCAGCCGTTGATGCAGAAGTTAAAAAAGAACTGCGTGAAGATCTGCTGGGTGGTCGTCTGGCATGGATTGGTAGCGGTTCAGCACCGCTGTCAGCAGAAATGGCAGCATTTATTGAAAGCTGTACCCAGCTGCCTGCCCATGATGGTTATGGTAGCACCGAAACAGGTCGTGTTCTGGTTGATGGTCGTGTTAGCCGTCCGCCAGTTCGTGATTATCGTCTGGATGATGTGCCGGAACTGGGTTATAGCCGTGCCGATAGCCCGTATCCGCGTGGCGAACTGCTGGTGCGTACCGCAGCACTGATTCCGGGTTATTATCGTCGTCCGGAACTGACCGCACGTCTGGTGGATGCAGATGGCTATTATCGTACCGGTGATATTATGGCCGAAATTGGTCCGGATGAACTGGCCTTTGTTGAACGTCGTGCACATGTTCTGAAACTGGCACAGGGTGAATTTGTTGCAGTTGCGCGTCTGGAAGCACTGTTTGCAACCAGTCCGCTGGTTCATCAGGTATTTGTTCATGGTGATTCAGAACGTGCATACCTGCTGGCAGTTGCAGTTCCGACCGCAGCAGCCCTGGATCGTGCGGGTGAAGATGGTCCTGCACTGCGTAGCCTGATTCTGGAAAGTCTGCGTCGTACCGCACGTGATGCCGGTCTGAGTCCGCATGAAATTCCGCGTGATGTTCTGATTGAAACCGAACCGTTTAGCGTTGAAAATGGTCTGCTGAGCGGTGTGCGTAAACCGCTGCGTCCGAGCCTGGAACGTCGTTATGGTGAAGGTCTGGAACGCCTGTATGCCGAACTGACAGAACGTGAAGCAGACGAACTGGGTGCCCTGCGTCGTGCAGGTCGTGATCAACCGGTTCTGGATGCAGTTACCCGTGCAGCACAGGCACTGCTGGGTCGTGTGAATGGTGAAGCACATCCGGAAGCCCATTTTCTGGATCTGGGTGGTGATAGCCTGAGCGCACTGAGCTTTGCAACCCTGCTGGCGGAAATTTTTGCGGTTGATGTTCCGGTTGGTGTGATTACCTCACCGGCACATGATCTGCGTAAAGTTGCAAGCCATCTGGAAACCGCACTGCGTTCAGGTCCTAGCGGTCCGACCTTTGCCACCGTGCATGGTGCAGATGCCACCCGTGTTCGTGCCACCGATCTGGCGCTGGATCGTTTCCTGGATGATCGCACCCTGGGAGCCCCTGCACGTAGCTCAGGTCCGGCACGCGTTGTGCTGCTGACCGGTGCAACCGGTTATCTGGGTCGCTTTCTGTGTCTGGAATGGCTGGAACGGCTGGCACCGACCGGTGGCCTGCTGATTTGTGTTGTTCGTGCAGCAGATCCTGGTGCCGCACGCGCTCGGCTGGCAGGCGCATTTGATAGCGGTGATCCAGACCTGGTTGCCCGTTTTCAACGTCTGGCAGCGCGTCATCTGGAAGTTGTTGCCGGTGATGTTAGCGAACCGGGACTGGGCCTGGATGAAGCAACCTGGCGTCGGCTGGCCGAAACCGTGGATCTGATTGTTCATAGTGCAGCACTGGTTAATCATCTGCTGCCGTATGAGCAGCTGTTTGGTCCGAATGTTGTTGCCACCGCAGGTCTGATTCGTCTGGCCCTGACAGCACGTACCAAACGTTTTACCTATGTTAGCACCATTGGTGTTGTTGCAGCCCAGAGCGCAGCACGTGCAGAAGATGCAGATATTCGCCTGGCCTCACCGGTTCGTGCACTGGATGGTCGCTATGCAAGCGGTTATACCGCAAGCAAATGGGCTGGTGAAGTGCTGCTGCGTGCAGCCCATGAACGCTATGGTCTGCCGGTGGCCGTTCTGCGTCCGGATCTGATCCTGGCACATAGCCGTTATGCCGGTCAGCTGAATCTGAGTGATGTTGTTACACGCCTGCTGTTCAGCCTGGCAGCTACCGGTCTGGCACCTCGTACCTTTGAAGCAGCCGATGCGGGTCGTGCGCGTGCACGTGTTGGTGGTCTGCCTGTTGATGTTACCGCAGCCGCAGTTGCGACCCTGGGTGCATGGCCAGATCAGGGTTATCAGACCTTTAATGTTCTGCAGCCGAGTGGTGATGGTCCTAGCCTGGATACACTGGTTGACGGTCTGGTTGAAGCAGGTTGTCGTCTGACCCGTATTGAAGATTATGCACAGTGGCTGGCACGTTTTGCGACCGCAGTTCGTGCCCTGCCGGAACGTCGCCGTCGTTATAGCCTGCTGCCGCTGCTGACGGCATTTGGTCAGCCTGGTGATCCGGTGGGTCGCCTGGGTTTTCCGACCGATCATTTTGCAACAGGTCCGGTTCCTGCCGATCGTTTTCTGGCTGCCGCAAGCCGTGCAGGCCTGGGTGCTGCTGTTGCCACACCGGGTATTAGCGCAGCCCTGCTGCGTAAATATGTGAGCGATCTGCGTGGCCTGGACCTGCTGTAA

Aa_ACAR

ATGACCACCGCAACAGGTCCGCGTCCGGATATTACCGCAGCAATTACCCGTCCGGGTGTTCGTGTTCAAGAAATTCTGCGTACCGTTCTGGATGGTTATAGCGATCGTCCGGCACTGGGTGAACGTGAAACCGTTCTGCGCACCGATCCGGTTACCGGTCGTCGTACCCGTGAACTGCTGCCTGGTTTTCGTACCATTAGCTATGGTGAACTGCGTGCACGTATTGAAGCAGTTGCAGCCGATCTGCGTCTGCAGAGCGGTGATGTTGTTGGTCTGCTGGGTTTTACCAGCCTGGATCATACCACCGTTGATCTGGCATGTGTTCATCTGGGTGCAGTTTGTGTTCCGCTGCAGAGCAGCGCAAGCGCAGCACAGCTGAAACCGGTTATTGCAGAAATTGAACCGCGTCTGCTGGCAACCAGCCTGCAGCTGCTGGATACCGCAGTTGAAGTTGCACTGAGCAGCACCAGCGTTCAGTGGATTGTTGTTTTTGATTATCACGAGGATGATGATGACGAACGCGAAATTTTTCTGGCAGCACAAGAACGTTTTGCAGTTGAAAGCCTGACCGATGTTCTGGAACGTGGTGCAGCACTGCCTGCACCGCCTGCACATGTTCCGCGTCCTGATGAAGATCCGCTGAGCCTGCTGGTGTATACCAGCGGTAGCACCGGCACCCCGAAAGGTGCAATGTATACCGAACGTCTGCTGCGTCGTCTGTGGCTGGGTTATCTGCCTCCGATTCCGGATCTGAGCGTTATTCGTGTGCATTATATGCCGATGAGCCATCTGACCGGTCGTGCAGCAGTTATTGAAACCCTGGTTGCCGGTGGTATTGGTTATTTTACCGCACGTAGCGATCTGAGCACCCTGTTTGAAGATATTGCACTGGCACGTCCGACCGAACTGTTTCTGATTCCGCGTCTGAGTGATATGCTGTTTCAGCGTTATCAGCGTGAACTGGATCGTGGTGTTCCGGAAGCAGAAGCAATGGCAGTTATTCGCGAAGCAGTTCTGGGTGGTCGTGTTGAACGTAGCGCAACCGGTAGCGCACCGCTGAGCGCAGAACTGGCAGCATTTGTTCAGAATTGTCTGGGTGTTCGCCTGCATGATGGTTATGGTAGTACCGAAACCGGTCGCGTTTTTGTTGATGGTAAAGTGACCCGTCCGCCTGTGATTGATTATAAAGTTGTTGATGTGCCGGAACTGGGTTATTTTCGCACCGATGCACCGCATCCGCGTGGCGAACTGCTGGTTCGTACCGAAGCAATGATTAGCGGTTATTACAAACGTCCTGATCTGAATGCCGAAGTGTTTGATGAGGATGGTTTTTATCGTACCGGTGATATTGTGGCAGAACCGGGTCCGGATCAGCTGGTTTATCTGGATCGTCGTAAAAATGTTCTGAAACTGAGCCAGGGTGAATTTGTTGCAGTTAGCCGTGTTGAAGCAGCCCTGGGTGCAAGTCCGCTGGTGCGTCAGATTTTTGTGTATGGTAATAGCGAACGTGCATATCTGCTGGCCGTTGTTGTTCCGACCGGTGCACATCGTAAAGAACGTATTAGCGAAAGCTTCCATCATATTGCACGTGAAGCAGGTCTGAGCAGCTATGAAATTCCTCGTGAATTTATTGTTGAAACCGAACCGTTTAGCACCGCAAATGGCCTGCTGTCGGATCTGAATAAACCGCTGCGTCCTGCCCTGCGTGAACGTTATGGTCCTCGTCTGGAACAGCTGTATGTTGAACTGGCCGAACGTGAAGCCGACGAGCTGCGTGCTCTGCGTGCAGCGGGTGCAGATCAGCCGGTGCTGCCTGCAATTCGTAGCGCAGCGCGTGCACTGCTGGGTAGCGCAGATGCAGATGCACGTTTTACCGATCTGGGTGGCGATAGCCTGAGCGCACTGAGTATGGCAAATCTGCTGCGCGATATTTTTGATGTGGATGTTCCGGTTAGCCTGATTATTAGTCCGGCAACAACCCTGCGTGGTCTGGCAGATCATATTGAACGCGCACCGCGTGCCGCACGTGGTCCGAAAACCTCAGTTCGTGCCGCAGATCTGACCCTGGATAAATTTGTTCGTCCGACCCCGAATACCATTGGTACAGTTCTGCTGACCGGTGCCAATGGTTATCTGGGTCGTTTTGTTTGTCTGGAATGGCTGCAGCGTCTGGCAGCGACCGGTGGTAAACTGATTTGTCTGGTTCGTGGTGGTAGCACCCGTCTGGAAGAAGCATTTGGCGATTCAGCACTGCTGCGTCGCTTTCGTGAACTGAGCGGTCATGTGGAAGTTGTTAGCGGTGATCTGAGTGAACCGGATCTGGGTCTGCCACGTCGTACCTGGGATCGTCTGGCCGAAACCGTGGATCTGATTGTTCATCCGGCAGCACTGGTTAATCATGTTCTGCCGTATGAAGAACTGTTTGGTCCGAATGTTGTGGGTGTTGCCGAACTGATTAAACTGGCAATTACCAAACGTCGCAAAGGCTTTACCTTTCTGAGCAGTATTGCAGTTGCCGAAGGTGAAGATACCGATGTGCGTCGTGCATCACCGGTTCGTGCACTGGATGGTAGCTATGCAAATGGTTATGCAACCAGCAAATGGGCAGGCGAAGTGCTGCTGCGTGAAGCACATGAACGCTATCGTCTGCCGGTTGTTGTGCTGCGTAGCGATATGATTCTGGCACATAGCACCTGTACGGGTCAGCTGAATGTTCCGGATATGTTTACCCGTCTGCTGTTTAGTCTGATTGCCACCGGTATTGCACCGCGTAGCTTTTATCGTTCTGATGCAGAACATTATGATGGCCTGCCGGTGGATTTTGTTGCACGTGTTGTTGCAGATCTGGGAGCACCGAGCGAAGGTTATCGCACCTTTAATGTTGTTAATCCGCATGAAGATGGCATTAGCCTGGATACCTTTGTTGATTGGCTGATTGAAGCCGGTCATCGTATCCATCGTATTGATGATCATGCAGAATGGTCACGTCGTTTTGAAATGGCACTGCGTGCCCTGCCGGAAAAACAGCGTAAACATAGCATGCTGCCGCTGCTGCATGCCGTTGCCGCACCGGTTGAACCGGGACCGCTGGTTCCGAGCGAACGTTTTCAGGCAGCCGTTGGTGAAATTCCGCAGGTTAGTCGCGAACTGATCGTTAAATATGCAGATGATCTGCGTAGCCTGGGACTGCTGTAA

Ks_ACAR

ATGAGCCGTAGCCAGCTGAGCGCAGATGAACTGGATGCACGTACCGATCGTCGTAGCGCACGTCTGTTTGCAACCGATGCACAGTTTCGTGCAAGCGCACCGCTGGATGCGGTTACCGAAGCAATTCGTCGTCCGGGTCTGCCGCTGGCAGATCTGGTTGCCACCGTTCTGGAAGCATATGCAGATCGTCCGGCACTGGGTGAACGTGCAACCGAACCGGTGACCGATCCGGAAACCGGTCGTACCACCCTGCGTCGTAGTCAGCGTTTTGATACCCTGACCTATGGTGAACTGCGTGATCGTGTTGATGCAGTTGCAAGCGATTGGCGTCATCATCGTGAACATCCGCTGGGCACCGGTGATTTTGTTGCACTGCTGGGTCCGACCAGCGCAGAATATACCGTTGCCGATCTGGCATGTGTTCGTAGCGGTGCAGTTAGCGTTCCGCTGCAGAGTGGTGCACCGGCAGCACATCTGGCACCGGTTGTTGAACAGGTTGGTCCGCGTCTGCTGGTTGTTGATGTTGATCAGCTGGATGTTGCCCTGGAAATTGCAGCAAATGCACCGAGCCTGGGTCGTATTGTTGTTATTGGTCATCGTCCGGAAGTTACCGTTCATCAAGAAGAATTTGAAAGCGCACGTGCCCGTCTGGCAGCACAGGGTCGTGGTGCAAGCCTGGATACCCTGGCAACCACCGTTGAACGTGGTCGTGCACTGCCGCTGCTGCCTCGTGTTCCGGATGGTAGCGCACCGGATACACTGAGCACCCTGATTCATACCAGCGGTAGCACCGGTAGCCCGAAAGGTGCAGTTTATACCGAACGTCTGGTTCGTCAGTTTTGGGTGGATTTTGTTCCGGGTCGTCCGGTGCGTCCGGCAATTGTTCTGAATTATCTGCCTCTGAGCCATATGATGGGTCGCGGTGTTCTGTATGGCACCCTGGCCAATGGTGGTCTGGCCTGTTTTACCGCAAGCGGTGATCTGAGTACCCTGCTGGAAGATCTGAGCCTGGTGCGTCCTACCGAATTTGTTATGGTTCCGCGTATTTGTGATATGCTGCTGCAGTATTATCGTGCAGAACTGAGCCGTCGTACCGGCACAGCCGAAGATGCGGAAGATCGTGAAAGCACCGAAGATACCGCAGATGCCGATGAACCGGCAGCCGCACAGCAGCTGGCAGAAGAACTGCGCGAGGAACTGCGGGAACAGGTTATGGGTGGTCGTCTGCTGTGGGCAGTGAGCGCCAGCGCACCTCCGAGCGCAGGCACCACCGCATTTGTTGAAGATTGTTTTCAGGTTCGTCTGATTGATGGTTATGGTAGTACCGAAGGTGGTGTTGTTCTGCTGGATGGTCGTGTTCAGCGTCCGCCTGTTACCGATCATAAACTGGTTGATGTGCCGGAACTGGGTTATTTTGCCACCGATAGCCCGTTTCCGCGTGGCGAACTGCTGATTCGTAGCGATCGCCTGGTTCCTGGTTATTTTCGCCGTCCGGATGCAACCCGTGAAATTTTTGATGAAGATGGCTTTTATCGCACGGGTGATATTATGGCACGTGTTGGTCCGGATGAACTGCGCTATGTTGAACGTCGTAGCAATGTGCTGAAACTGAGCCAGGGTGAATTTGTGGCAGTTAGCCGTCTGGAAGCCCTGTTTGGTGGTGGTCCGGCAGTGCGTCAGATTTTCCTGTATGGTAGTGGTGCCCGTGCACATCTGCTGGCAGTTGTTGTTCCGACCCAAGAAGCACTGGATCGCGTTGGTGGTGATGCACGTCGTCTGGGTCCGGTTCTGCGTGAAAGCCTGCGTCGCACCGCAGTTGGTGCCGAACTGAATCCGTATGAAATTCCGCGTGATCTGCTGGTAGAAACAGAACCGTTTACCGAAGAAAATGGTCTGCTGAGCGGTGTTCGTAAACTGCTGCGTCCTGCACTGACCAAACGTTATGGCGAACGCCTGGAAGCACTGTATACGGAACTGAGCGATCGCGAAACCGAAGCCGTTCGTACACTGCGTCGTTTTCGTGCCGATCGTCCTGTTCCGGAAACAGTTGTTCGTGCAGCCCAGGCGCTGCTGGGCCTGGAAGATGGTGCAGTTGAACCGGGTACACGTTTTCTGGAACTGGGAGGTGATAGCCTGAGCGCACTGAGCTTTAGTCGTCTGATGACCGAAACCTTTGATGTGGATGTTCCGGTGGATGTTGTGCTGAGTCCGGTTAGCAGCCTGCAGGGTGTTGCAGATCATATTGAACGTGCCCTGGCCACCGAACCTCTGGCAGCCGAACATCCTCGTCCGACAGCGGAAGGTGTTCATGGTCCTGATCGTACCCGTCTGGATGCAGCACGTCTGCGCCTGGATGCATTTCTGGACGCTCGTACCGTGGAACGTGCAGGTCGTCTGAAACCGGATGGTCCGCTGCCGGAAGCCCGTACCGTTCTGCTGACAGGTGCAAATGGTTATCTGGGTCGTTTTCTGTGTCTGGAATGGCTGGAACGCGCAGCCCGTCGTGGTGGTACACTGGTTTGTGTTGTGCGTGGTAGCAGCAGCGAAGCAGCGCGTGCACGTCTGGACGCAGCCTTTGATAGCGGTGATGCGGAGCTGCTGGAACGTTATCGTGCCAGCGCAGCGCGTCATCTGCAGGTTGTAGCCGGTGATGTGGGTCAGCCTCGCCTGGGTCTGGATGAAGAAACCTGGCAACATCTGGCCGATACCGTTGATCTGATTGTTCATCCTGCAGCACTGGTTAATCATGTTCTGAGCTATGATCAACTGTTTGGTCCGAATGTTCTGGGTACAGCGGAACTGATTCGTCTGGCCGTTACCAGCCGTATTAAACAGTTTAACTATCTGAGCACCGTTACCGTTGTGCTGGGTAATGCAGCCGCAGCAGATGAAACCGCAGATATTCGTACCACAAGCCCGACCCGTGATCTGGATGGTGATCGTTATGCAGATGGCTATGCAGCAAGCAAATGGGCAGGCGAAGTTCTGCTGCGTGAAGCACATGAAACATTTGGTCTGCCGGTTGCAGTTTTTCGTAGTAATCTGATTCTGGCACATCCGCATTATCGTGGTCAGCTGAATATTCCGGATGTTTTTACACGTCTGCTGCTGAGCCTGCTGGCAACAGGTCTGGCTCCGGCAACCTTTTATGCACGTGGTACAGGTGATGGCCGTGGTCATTTTGATGCCCTGCCTGTTGATTTTACAGCACGTGCAGTTACAGCCCTGGGTGATGATGCCCGTGAAAGTCATCGTACCTATAATGTTGTTAACCCGCACGAAGATGGTATTAGTCTGGATACCGTGGTTGATTGGCTGGTTGCAGCAGGTCATCCTCTGGCACGCGTTCAAGAACATACCGAATGGACCGAACGTTTTGGCACCGCACTGCGTGCCCTGCCGGATGGCCTGAAACAGCATAGCCTGCTGCCACTGCTGCATGCATTTGCAGAACCGCTGGAACTGCTGGCTGGTTCTGCACTGCCTGCAGATCGCTTTCGTACCGCAGTTCGCGCAGCAGCCCTGGATCCTGAAAATGATATTCCGTGTCTGTCACCGGATCTGATTACCAAATATGCAGCCGATCTGCGTGCGCATCGTCTGATCTAA

Ss2_ACAR

ATGAGCGTTCCGGGTTTTGCACAGGATGTTCAGGATCGTGTTCGTGCACTGATTAAAAGCGATAGCCATTTTCGTGCAGCACTGCCGAGTCCGGATATTTCAGCAATGACCCGTCGTGATAGCCTGCGTCAGATTGAACTGCTGACCGCAGTTATTGAAGGTTATCGTGATCGTCCGGCACTGGGTCGTCGTGCAACCGAACTGGCAGCAGATCCGGTGACCGGTAAAACCGCACGTCGTCTGCTGGATCGTTTTGAAACCATTAGCTATCATGATCTGCTGAGCCGTGTGCGTGCCCTGGCAGCATTTTGGTATCATCATATTGCACGTCCGCTGCGTCCGGATGATATGCTGTGTGTTATTGCAGCAAGCGGTTTTGATTTTACCACCATTGATATTGCCAGCATTCATCTGGGTGCAGTTCTGGTTCCGGTTCAGGCAAAAGCACCGGCAGCACAGCTGCTGAGCATTCTGGATGAAGTTCAGCCTCGTTGGCTGGCAGCCAGCGTGGATAGCCTGGATAGCGTTGTTGAACTGGTTCTGGCAGGCGTTCGTCCGGTTGGTATTCTGCTGTTTGATTATCATCCTGAAGCAGATGATGAACGTGAAGCACTGGAACGTGCAAAAACCATTCTGGCAGAAAGCGGTCATGATGGTCTGATTGTTACCCTGAGTGATGCAATTGAACAGGGTGGTCGTCTGGCTCCGGCACCGGCATATCTGGATGGTGATATGGAACGTCGTGTTAGCACCATTATCTATACCAGCGGTAGCACCGGTCAGCCGAAAGGTGCAATGTGGCCTGAAAAAACCATGACCGGTAGTCTGCGTGCATTTAGCAGCCTGCCGTCAATTGTTCTGCATTATGCACCGATGAATCATAGCATTGGTCGTCCGGGTGTTTTTGCAACCCTGTGTGCGGGTGGCACCTGTTATTTTACCGCACGTAGCGATCTGAGCGAAGTTCTGAATGATATTAGCCTGGTTCGTCCGACCCAGATGTTTTTTGTTCCGCGTGTTTGCGAACTGCTGTTTCAGCAGTATCAGATCGAACTGGATCGTCTGAAAGCCGGTGCGGATGATGTTGGTGCAGTTGAACGTAGCCTGATTCTGGATATGCGTGAACGTGTTCTGGGTGGTCGCCTGCTGAGCGCAGCATTTGGCACCGCACCGCTGTCACCGGCACTGAAAACCTTTATGGATCAGTGTCTGGGTTTTCCGCTGGTTGATGGTTATGGTAGTACCGAATGTGGTCGTGTTCTGCTGAATAGCCAGGTTCTGCGTCCGAGCATTATTGATTATCGTCTGGATGATGTGCCGGAACTGGGCTATTTTAACACCGATAAACCGCATCCGCGTGGTGAACTGTGGGTTAAAAGCAGCACCATGTTTCTGGGTTATTTCAAACGTCCTGATGTTACCGCAAGCGTTATGACCGCAGATGGTTTCTATAAAACCGGTGATATTATGGCAGAAATTGCTCCGGATCAGCTGGTTTATCTGGATCGCCGTAATAATGTTCAGAAACTGGCACAGGGTGAATTTGTTGCAATTGCCCAGCTGGAAACCCTGTTTACCAATGGTGATGCACGTATTCGTCAGGTTTTTCTGTATGGCACCAGCGATCGTAGCTTTCTGCTGGGTGTTGTTGTTCCGAGCGAAGATGCCGTTCTGCAGAGCGGTATTGATCCGGAAGGTGAAACACGCCTGAAAGCGGAACTGCAGGCAGCCATTAAAAACGTTGCACTGAAAGAAGGACTGAAAGCATACGAAATGCCTCGCGATTTTATTGTGGAACTGAGCCCGTTTACCGTTGGTAATGGTCTGCTGGCAGATGTTGGTAAATATCAGCGTCCGAATCTGCGTAAACATTATGGTCCGCGTCTGGAACGTCTGTATGAGCAGATTGCAGCAGGTCAGGCAGATGAACTGATGCTGCTGCGTCGTAATGCACGTGATATGCCGCTGCTGGATACCGTTATTCGTGCAGTTCGTGCCACCCTGGGCCTGGATGACGTTGATCTGAGTGGTAGCACCAAATTTAGCCGTCTGGGTGGCGATAGCCTGAGCGCACTGAGCTGTAGCCTGCTGCTGGAAGAAATTTATGATGTTGATATTCCGGCAGCCGTTATTAATGGTCCGGGTGGTACACTGCAGCATATTGTTGATCATATCGCACGCGCACTGTCACAGGATCGCGGTCGTCCGACATTTGCAAGCGTTCATGGTCGCGGTGCCACCGAAATTCGTGCCGCAGATCTGAAACTGGAAAAATTTCTGGATGCACCGAGCCTGGATGCAGGTCATGCGGTTCTGCCTGCACGTCAGGATGTGCGTACCGTGCTGGTTACCGGTGCAACCGGTTTTCTGGGTCGTTTTCTGTGTCTGGAATGGCTGCAGCGTATGGCAGCAGTTGGTGGTCGCGTTATTTGTATTACCCGTGGTCAGGATGCAGCAGATGCATATCGTCGTATTGCAGATATGTTTGAAAGCGGTGATGCCGATCTGCGTCGTCATTTTAGCACCCTGGCCGATGGTGCACTGACCGTTCTGCGTGGCGATCTGTCAGAACCTGATCTGGGTCTGAGTCGTGCAGATTGGCTGGATCTGGCCGAACGTGTGGATCTGATTGTGCATCCGGCAGCGCTGGTTAATCATCTGCTGCCGTATAGCCAGCTGTTTGGTCCGAATGTTGTTGGTACAGCAGAACTGATTCGTCTGGCAATTACCCACCGTCTGAAACCGTTTATTAACCTGAGCACCGCAACCGTTCCGATGCTGCCGAGCCTGAGTCCGGTTGATGAAGATGCAGATGTTCGCATTGCCATGCCGGTTCATCAGCCGGATAATGCCCGTTATGCCGATGGTTATACCCTGAGCAAATGGGCTGGTGAAGTACTGCTGCGTGAAGCCCATGATCGTTATCGCCTGCCGGTTAGCGTTTTTCGTAGCAGTATGATTCTGGCACATGAAGCATATGCGGGTCAGATTAATGTTCCGGATGTTTTTACACGCCTGCTGTTTAGTGTTATTGTGACCGGTCTGGCACCACGTAGCTTTTATGCGGGTGATGGTGCAGCAGCACATTATGATGGACTGCCGGTGAATTTTACAGCAGCCGCAATTAGCGCACTGGGTGCCGGTGTTCTGAGCGGTTATCGTAGTTATCATGTTGTTAATCCGCATGATGATGGTGTTAGTCTGGATACCATTGTTGGTTGGATTGCAGCCAGTGGCCGTCCGATTGAATTCCTGGATGATTATGCACAGTGGATTACTCGCCTGGAACCTGCACTGCGTAGCCTGCCGGAACGTCAGCGTAATCAGAGTATTCTGCCGATTATTGATCATTTTGGTCAGCCTGCTCCGGCAGTTCCGGGTATGCCTCTGGCCACCGAACGTTTTCGTGCCGACCTGGATCGCTTTGCACCGGAAGGCGCACGTACCATTCCGCATCTGACACGTGCCTTTGCCCTGAAATACCTGTCTGATCTGCGTGCGCTGGGTATGGTTTAA

Ge_ACAR

ATGAGCGATCAGCCGAATGCACTGCCGAGCGCAATTGAACCGCTGAATCCGGATCCGCAGGCAACCGAGCAGATTAGCCATTGTGCAACCATTGCAGAACTGGTTCGTGTTCTGGCAGAAAGCTATGGTGATCGTCCGGCACTGGGTTGGCGTAATAATAGCGATCCGAGCAGCTGGCATAGCATGACCTATCGTGATCTGGCCGAACGTGCAGATAGCATGGCACGTCTGCTGCATAGCACCCTGGGTGTTGCAGAAAATGATCGTGTTGCAACCGTTGGTTTTACCAGCGCAGAATATACCATTGCAAGCCTGGCAGTTGGTACACTGGGTGCAATGGAAGTTCCGCTGCAGAATGCAGGTAGCGTTGATGTTTGGGCAGCAATTCTGACCGAAACCGATTGTGTTAGCGCAGTTGTTGCAGCAGATCAGCTGCCGAGCATTGCCCGTCTGGCGGAAAGCGGCACCTATACCGGTCTGCGTCATGTTCTGGTTTTTGATATTGGTAGCCGTGATGGCACCACCCTGGATGATGCAGCACGTCGTCTGGTTGCCGCAGGCACCCAGGTTCATCTGCGTCAGCCTGGTGCAGAACCGACCACCCCTCCGGCACCGCTGCCGCAGATTACCGCAAACCCGGATCGTGTGGCACTGCTGATTTATACCAGCGGTAGCACAGGTGCACCGAAAGGTGCAATGTATACCGAAACAGCAGTTACCCGTCTGTTTCAGAGCGGTCTGAGTGGTCTGGGTCGTGCAACCGATGGTCATGGTTGGATTACCCTGAACTTTATGCCGATGAGCCATGTTATGGGTCGTAGTACCCTGTGGCAGACCCTGGGTAATGGTGGCACCGCATATTTTACACCGCGTGCAGATCTGGCTGAACTGCTGACCGATCTGGCAGCCGTTCAGCCGACGGATCTGCAGTTTGTTCCGCGTATTTGGGATATGCTGTATCAAGAATATGTTCGTCTGACAGATCAGGATGTTAGCGAACAGGATGCACTGACCCGTATGCGTGAACATTATTTCGGCACCCGTACCGCAACCGCAATTACCGGTAGCGCACCGATTAGTGATGAAGTTCGTCGTTTTGTTGAAGCAATGCTGCCGGTTCCGCTGATTGAAGGTTATGGTAGCACCGAAGCAGCCGGTGTTAGCATTGATGGTCGTATTCAGCGTCCGCCTGTTGTTGATTATAAACTGCTGGATGTGCCGGAACTGGGTTATCTGAGCACCGATCGTCCGCATCCGCGTGGTGAGCTGCTGGTTAAAACCGATCATATTTTTGCCGGTTATTACAATCGTCCGGATCTGACCAGCAGCGTTTTTGATGATCAGGGTTATTATCGTACCGGTGATATTGTTGCCGAAACCGGTCCGGATCAGATTGAATATGTTGATCGTCGTAACAACGTGATGAAACTGAGCCAGGGTGAATTTGTTGCAATTGCCCATATTGAAGCAGTTCTGACCACCCCACCGATTCAGCAGCTGTATGTTTATGGTAATAGCGCACGTCCGTATCTGCTGGCCGTTGTTGTTCCGACACCGGAACTGCGTGAACGTCATGCAGATGATAATGAACTGCGTCGTGAAGTTCTGACAGCACTGCGTAGCCATGGTGAACGTAATGGTCTGGCAGCAGTTGAAATTCCGCGTGATGTTATTGTTGAACGTACCCCGTTTAGCCTGGAAAATGGTCTGCTGACAGGTATTCGTAAACTGGCACGTCCGCAGCTGAAAGAACGTTATGGTGCACGTCTGGAAGCACTGTATGCCGAACTGGCCGATAGCCGTATTACACGTCTGCGTGATGTGAAAGCAGTTGCAGCCCAGCGTAGCACCGTTACCACCGTTATTGATGTTGTTACCGCAATTCTGGATCTGGCGGATGGTGAAGTTACCGCAGCAGCACATTTTACAGATCTGGGTGGTGATAGCCTGACCGCAGTTACCGTTGGTAACGAACTGCGCGATATTTTTGATGCCGAAGTTCCGGTTGGTGTGCTGACCAGCCCGAGCAGTACCCTGGCAGATATTGCGGAACATATTGATGGCCGTCATAGCGAAGCACGTCCGACCGCAGAAAGCGTTCATGGCACCGGTACAACCCTGCGTGCAGCCGATCTGACCCTGGATAAATTTCTGGATGAAGAAACACTGCGTGCCGCAAGTGATGTTACCAGTGCAGCCACCGATGTTCGTACCGTGTTTATTACCGGTGCAACCGGTTTTCTGGGTCGCTACCTGACACTGGATTGGCTGCGTCGTATGGCAAAAGTTGGTGGTACAGTTATTTGTCTGGTGCGTGGTGCCGATGATGACGCAGCCCGTGCGCGTCTGGATGCAGCATTTGATAGCAGCGATCTGTGGTCTGAATATCAGCGTCTGGCAAAAGATCATCTGCGCGTGCTGGCAGGCGATAAAGATAGCGATCATCTGGCACTGACACCGGATGTGTGGGATGAACTGGCAAAAAGCGTTGATCTGATTATTGATCCGGCAGCACTGGTTAATCATGTACTGCCGTATCGCGAACTGTTTGGTCCGAATGTTAGCGGCACCGCAGAACTGATCCGTCTGGCAGTTACCACCACCCGTAAACCGTATGTGTATATTTCAACCGTGGGTGTTGGTGATCAGGTTGCTCCGGGTAGCTTTACCGAAGATCCTGATATTCGTGAAATGAGCAGCGTGCGTGAAATCAATGATACCTATGCAAATGGTTACGGCAATAGCAAATGGGCAGGCGAAGTTCTGCTGGCACAGGCACATGAACGTTTTGAACTGCCGGTTAGCGTTTTTCGTTGTGATATGATTGTTGCGGATGATCATACCATTGGTCAGCTGAATCTGCCGGATATGTTTACTCGCCTGCTGATGAGCGTTCTGGCAACAGGTCTGGCACCGCGTAGCTTTTATCAGCTGGCGACCGATGGTAGTGCACAAGAGGCACATTTTGATGCGCTGCCGGTGGATTTCCTGGCCGAAGCAATTAATACACTGTGGGTTAAAGATGGTGCCCGTACCTTTAATGCAATGAATCCGCATGCCGATGGTATTGGTTTTGATCAGTATATTCGTTGGCTGATTGATAGCGGTGAACAAATTAGCCTGGTGGATAATTATGATGATTGGTATCGTCGCTTTGGTGCCGCACTGGCGGATCTGCCTGAAAAACAGCGTCGTGGTAGCCTGATTCCGCTGCTGCACAATTATGTTCATCCGATGACACCGCATAATCGTGGTATGGCAAGCGCAGATCGTTTTCATGATGCAGTTCGTACAGCAGGCGTTGGTCAGAGCAGCGATATTCCGCATATTACCCCTCAGATTATTGAAAATTATGCACGTAGCCTGCGTGGCCTGGGTGTGATTTAA

Nm_ACAR

ATGGATGCAGAAAGCGGTGTTATGCCGAGCGATCCGGATGTTGTTGCATTTGTTCGTCGTCCGGATACCTGTCTGAGCGCAATGATTGAAAAAGCACTGGATAGCTATGCAGGTCGTGATGCACTGGCATGGCGTCCGACCCGTAGCGGTGTTCTGAGCGATACCTTTGAAGTTATGACCTATAAAGATCTGGCACGTCGTGTTCGTAGCGTTGCAACCGCACTGGCAAAAGATCCTGATCTGGGTCTGAAAGCCGGTGATCCGATTGGTATTATGGCATTTGCCAATGTTGATTTCGTTACCCTGAATCTGGCACTGGGTCTGTGTGGTGGTGTTATTGCACCGCTGCAGACCAGCGCAAGCATGGAAGCACTGACCGGTCTGGTTCGTGAACTGGCAGCACCGTGTCTGGCAGCAAGCCTGGAACATCTGGAAGCAATTACCACCCTGGCAATTGCAAGCGAAAAAACCCGTGCAATTCTGATCTTTGATCATGATGGTCTGGATGAAGATGCACGTGCCGCAATTGCAGCAGCACAGCAGCGTCTGGATGCCGAAAAACCGGGTTGTCTGATTCTGCCGTTTAGCGAAGTTATTGCCCGTGGTGAAAAACTGCCTCCGCTGGATCCGTTTGTTCCGGCACCGGGTGAAGATCCGCTGGCACTGATCTATTATACCAGCGGTAGCACCGGCACCCCGAAAGGTGTTATGTATACCCAGAAACTGGTTAAACTGGGTTATAGCATTGCACGTGATCATGCACCGATTGTTCTGCATTATCAGCCGCTGAATCATAGCTTTGGTATGAGCTTTATTGCAATGGCACTGGCGAGCGGTGGCACCAGCTATTTTACCGCAAAAAGCGATCTGAGCACCCTGCTGAGTGATATGAAAATGGTTCGTCCGACCACCATGGCCCTGGTTCCGCGTATTAGCGAAATGCTGTTTCAGCGTTTTCATGCAGATTATGCAGATGAAATTGCGCGTGATGAGGATGCAGCCATGGCACGTTTTCGTGAAGATGTTCTGGGTGGTCGTATGACCGATATTGTTACCGGTGCAGCACCGACCAGTCCGGAACTGCGTGATTTTATTGAAAAAATGACAGGTCTGACCCTGATGGAAGGTTATGGTTGTACCGAAGCCGGTGGTAGCATTACCTTTAATGATCGTGTTATGCGTCCGCCTGTTCTGGAATATCGTCTGATTGATGTTCCTGAACTGGGATATTTTACCACCGATACACCGCATCCGCGTGGTGAACTGATTCTGAAAAGTGATGCAATGTTTGCCGGTTATTTTGCACGTCCGGATCTGACCGCAAAAGCATTTGATGATGAAGGCTTTTATCACACCGGTGATATTATGGCCGAAATTGAACCGGATCATCTGGTTTATCTGGATCGTACCAATAACGTGATGAAACTGAGCCAGGGTGAATTTGTGCCGGTTGCACTGCTGGAAAGCCTGTATGCCGGTGGTGATCCGGTGATTCGTCAGATTTATCTGTATGGTAATAGCACCCGTGCCTTTCTGCTGGGTGTTGCAGTTCCGAATATGGATGCACTGCCGGAAGGTATTGGTGATGAAGAAATTAAAGCACGTATGCTGCAGGCACTGGAACGTATTGCACGCGCAAATGAACGTCATAGTTATGAAGTTCCGCGTGATCTGATTATTGAACACGAACCGTTTAGTCCGGAAAATGGTCTGCTGGCAGGCGTTGGCAAATATATGCGTCCTGCATTTAAAGCCCGTTATGGTGAGCAGCTGGAAGCCCTGTACGAAGAAATTGCACGTAGCCAGGATCGCGAACTGCAAGAACTGCGTCGTACCGGTCGTACCTTTCCGGTTCTGGAAACCGTTTGTCGTGCAGCCGGTGCAGTGCTGGGTGGCAAAACCGTTGCACTGAGCGAAACCGGTAGCTTTGCAAGCAGTGGTGGTGATAGCCTGAGCGCACTGAGCCTGAGCCTGCTGCTGGAAGATATTTATGAACTGCCGGTTGAAGTTAGCGCAATCCTGCATCCGAGCGGCACCTACGGCCTGCTGGCTGCAGAAATTGAAGAAAAACTGGGTGGTGGTGCAAAACGCCGTGATGCAGTTGCCGTTCATGGTGCAGATCTGACAGTTCTGAAAGCAGCCGATCTGACACTGGATAAATTCATTGATGCGGAAATTCTGGGTGCAGCAACCGGTCTGCCTGCCCCTCCTGATGCCGAACCGGAAGTTGTTCTGCTGACCGGTGTTACAGGTTTTCTGGGTCGTTTTATGTGTCTGGAATGGCTGCGTCGTCTGGAACGCAATGGTAAAGGTAAAGTTGTTTGTGTTGCACGTGGTGCCGATGATGATGACGCACGTCGTCGCGTTCTGGCAGGTTTTGAAGGTGGTGATGGTGCCCTGGCAGCCGAAGTTGCACGTCTGGGTGAAGGTCGTCTGGCCGTTTTTGCGGGTGATCTGGCAGCCCCTCGTCTGGGCCTGACCGCCACATGTTGGCAGGCCCTGTGTGATCAGGTGGATCTGATTGCACATCCGGGTGCCTTTGTTAATCATAAACTGCCGTATCGTCAGCTGTTTGGTGCAAATACCGCAGGCACCGCAGAACTGATTGCCCTGGCCCTGACCACCCGTAAAAAACGCTTTGCACATGTTAGCACCATTGCCACCACCTATAATGATGGTCATCGTGCGGATGAAAACGGCTATATTGATAGCGCCATCCCGGAATGGCATGTTAGTGATGCATATGCCGATGGTTATGGTAGCTCAAAATGGGCAGCGGAAGTGCTGCTGGCACGTGCGAATGAACAGTATGGTCTGCCGGTGAGCGTTTATCGTAGCAATATGATTATGGCACCTGGTGAATTTAGCGGTCAGCTGAATGTTCCGGATATCTTTACCCGTCTGCTGCTGAGCCTGGCGCTGACCCGTCTGGCACCGGCAAGCTTTTATAGCGGTGATAGCGCACGTGCACATTATGAAGGCCTGCCGGTGGATTTTCTGGCTCGTGCCATTGTGACCATTGCCGAAGATAATCGTGCAGGTTTTCATACCTTTCACACCATTAATCCGCATGATGATGGTATTAGCACAGATACATTTGTGGATTGGATTGGTGAAGCAGGTATTCCGATTGAACGCATTGCGGATTATGATGAATGGGTTACCCGTTTTGGCACCGCACTGCGTGCCCTGCCGGAAAAACAGCGTCAGGCAAGCATTCTGCCGCTGATGGATGCATATAAACATCCGAGTCCGGCAATTCCGGATTTTCGCGATCAGGCACCGAATTTTCGTCAGGCAGTTGCGGAAAGCCAGGTTAATGGTGATGGCGCAATTCCGCATCTGACACCGGCACTGATTGCGCGTTATCTGGAAGATCTGAAAGCGACAGGCCTGCTGACCAGCTAA

An_ACAR

ATGAAACGCTGCATCAAAGACGCAAAAAGCAAACGTATTGTTTGCCAGCTGACCGCAAAATATCAGACCATTACCTATCAGCAGCTGTGGTCACGTGCAGAAAGCATTGCAAATGAATGGTATCAGCATGATCAGTATCCGCTGAAAGCAAGCGATAAAGTTGCAATTCTGAGCTTTATCCACAGCGATTATATTGCCATTAATCTGGCCTGCGTTCAGATTAACGCAATTATTGTTCCGCTGCAGACCAACCTGAGCATTAAAGAACTGACCCTGATTCTGCAAGAAATTGAACCGCGTATTATTGCAGCCAGCATTGAATATCTGCCGATTGCAGTTGAACTGGCCAAAAACAATAATAGCATCAAACGCATCATCGTGTTCGATTATGATCCGAGCCATGATAATGCAGAAAAACTGGAACAGCTGCAGAACCAGCTGATTATCAAAATTGAAGAACTGCCGAACATTATTCGTCTGGGTAGCCAGCTGCCGAAAGTTCCGTATCCGGAAAATAGTGATGATACCAGCAGCATCTCCATGATTATCTATACCAGCGGTAGCACAGGTGCACCGAAAGGTGCAATTTATACCGAAAAATTTGTGAGCAATATGTGGGATGCAAGCCTGTTTGCAAATAACACCAATAAAGAAAATCGCACCGTTCTGTATCTGCCTATTTGTCATGGTCTGGCAAGCCAGCAACTGTATAACCAGCTGGCCAAAGGTGTTACCTGTTATCTGGTTGCCAAAAGCAATCTGAGCACCCTGTTTGAAGATATTACCCTGGTTAAACCGACCGAACTGCTGCTGATTCCGCGTGTTGCAGAAATGATCCTGCAACTGTACCAGAGCGAACTGGAAGGTCGTAAAAAAACCATTCATGATCCGCTGCTGGATAGCAAACTGAAAAAAGAAATTCGCATCAACATTTTTGGTGGTCGTGTTACCCAGATCTTTTATAGCAGCGCACCGCTGACCAATAAACTGACCGATTTCATTGAAAGCCTGTTCGAAGTTAAACTGCTGAATATGTATGGTAGCACCGAAACCCTGGCCATTTGCATTAATAACAAAATTCTGAAACCGCCTGTGGAAGATTACAAACTGATTGATGTTCCGGTGCTGGGTTATTATAGCACCGATAAACCGTATCCGCGTGGTGAGCTGCTGCTGAAAACCGCAACCATTATTCCGGGTTATTACAAACATCCGGAACTGTATAGTCAGCTGTTTGATGAACAGGGCTATTACATGACCGGTGATATTGTTAAAGAAACCGCCAAAGATCATCTGGTGGTGATTGAACGCAAAAAAAACGTGATTAAACTGAGCCAGGGTGAATTCATTACCACCACCATGCTGGAAACCCTGCTGAAAGATAGTCCGCTGATTAAAGACATCTTCATCTATGGTAATAGCGAATGGTCATATCTGCTGGCAGTTATTATTCCGATCCCGGAACTGCTGTATCGTTACAATAAACAGCAGCGTGAAATTAAACGCCTGATTCATCAGAGCCTGGAAAAAATTGCAAAAGACAGTGGCCTGAAACCGTATGAAGTTCCGCGTGATTTTCTGATTGATACCGAACCGTTTAGCCAGAAAAATGGTCTGCTGAGTGAACTGGGTAAACCGCTGAAACAGAAAATCGAAGCCCATTATATTGATGATCTGAACAAACTGTATCAAGAGATCTGCAACTACGATTTTAGCAAATTCTCCCAGCAGATCAACAAAGAGAACATTCTGGAAACCGTTATCAAACTGACCCAGTATCTGGTGGGTAGTCCGGGTATGGTGATTAATAGCGCAGCAACCTTTTGCCAGTTTGGTGGCGATAGCCTGAGTACCCTGCAGTTTAGCCTGGAACTGGAAAAAATCTGGGGTGTTATGATTCCGGTTGATATGATTGCAAATCCGACCTGTACCCTGGATGATATTGCAGATTATATCAAAAGCAACCAGCACATTATTAACAGCTGTCCGACCTTTGCAACGATTCATGGCATCGACAAAAAAAAAATTTATGCCTCACAGCTGGCCCTGGATAAATTCATTGATCCGGAAATTTTCCAGCAGATTAAAAACAGCAGTCGTAGCCTGAGCAGCTTTCATAATGTTCTGCTGACCGGTGCAAATGGTTATCTGGGCAAATTTCTGTGTCTGGCACTGCTGGAAGAACTGAATAAAACCGATGGTAAACTGATTTGCGTGATCCGCGAAAAAGATAATGAAAGCGCAAAACAGCGCCTGATGAATACCTTTAGTCCGCATAATGATCAGCTGGCGCTGAAATTCAAACAACTGGCAGATAAACATCTGACCGTTTATGCCGGTGATCTGACAAAACCGAAACTGGGTCTGGATGAAAAAACCTGGCATTATCTGAGCCAGAACATCGATCATATTTTTCATGCCGGTGCACTGGTGAATCATATTCTGCCGTATCAGCACCTGTTCGAGACAAATGTTCTGGGCACCGCAGAACTGATCAAACTGGCTCTGGTTAATCATCTGAAACCTTTTATCTTTATTAGCAGCATTATTGTGGCCATCCCGAGCGATAATACCAAACCTCTGAATGAAGATGCCAATATTTGTGAAGCCATCCCGTATCAAGAAATCAATAATCAGTATGCCAACGGCTATGCCATTAGCAAATGGGCAAGCGAAATTCTGCTGTATGAAGCCTATAACCGTTTTAAACTGCCGGTTACAATTTTTCGTCCGAGCATGATTCTGGCACATCGTCTGTATGATACCGAGTTTAACATTACCGATGTTTTTACCCGTCTGCTGCTGTCAATTATCAACACCAAAATTGCTCCGAAAAGCTTCTATCAGAGCAATAGCAATCTGTCACCGCATTATAACGGTCTGGCAGTTGATTTTGTTGTGAGCAGCATCATCAAACTGTCGAAAAATAACCATAATCAGCGTCTGACCTTCAATATGGTTAATCCGCAGAATGATAAAGTGAGCCTGGATACCATTATCGATTGGCTGATTAACAGCGGGATCAACATCAAAAAAAGCATGATTATGAAAAACGGCATCAACAACCTGAATTGGCCTCATGGTAAAATCACCTAA

Sc_ACAR

ATGGATCCGAGCAGCGAACCGCGTGATCCGCAAGAAAGCGAAATTCCGCAGAATGATGATAGCAATGAACTGCTGCTGACCCGTAGCCTGGAACGTTGTAGCCGTCTGGTTCAGACCGATGAAGAACTGCGTCGTGCACTGCCGAGTCCGGTTGCACTGGAAAAAATTCGTAGCTGTCATACCACCATTGAATGTGTTGCAACCGCATTTGAACTGTATGCAGATCGTCCGTGTATTGGTCATCGTCCGCTGGATGCAGCAGCAACCGCAGCAGATGGTGGTAGCGCACCGCGTTATCTGCCTGAATTTCGTGCAGTTAGCTATGCAGATATGTGGTCACGTGTTGAAGCATTTGCAAGCGGTCTGCAGCATGAAAAACTGGCAAATACCGGTAATTTTGTGGGTATTAGCGGTTTTGGTAGCACCGATTGGGTTGTTGCAGGTCTGGCATGTATGTATCTGAGCGCAGTTAGCGTTCCGCTGCAGACAGATCTGAGTCCGGCAGATCTGGAACTGATTGTTGCCGAAGCAGAACTGGCATGTGTTGTTTGTAGCGTTGGTCAGCTGGCACGTATTGAAGATATTCTGCCTCGTTGTCCGAGCGTTCGTAGCGTTGTTGTTATGGATCTGCTGGAAGGTGATCGTTGTGGTCATAGCGAACTGGAACGTGCCCGTCGTGCCCTGCGTCCTCTGGAAGCACGTGGTCGTCGTCTGAGCGTGCGTCCGATGCATGAAGTTGAACGTCTGGGTCGTCAGCAGGGTATTCTGCCGAAAGTTCTGCCTGCACAGCGTGGTGAACCGGATCCGCTGATGACCCTGATGTATACCAGCGGTAGTACCGGTAGCCCGAAAGGTGCAATGGTTCCGGAAAGCCTGTGTCGTCGTTATTGGCAGCTGGCCTTTACCCGTAGTCAGGATCCGCGTCTGGACCTGCTGCCGCATGTTGGTCTGAATTATAGCCCGATGAATCATTTTATTGGTCGTAGCCAGGTTGGTCGTTCACTGATGCGTGGTGGTATTACCCATTTTGTTCTGAAAAGCGATATGAGCACCCTGTTTGAGGATATTCGTCTGGCACGTCCGACCACACTGTTTCTGGTTCCGCGTATTGCAGAACTGATTCATCAGCAGTTTCAGGCGGAAGTTCTGCGTCGCGCACGTGCACTGGGTGCCGGTGGTGATGATGCAGCACGTCGTCGTATTGAACGTGAAATTATGGCAGAAATGCGTGGTAGCCTGCTGGGTGATCGTCTGCTGCATGCAACCATTGGTAGTGCACCGACCCCTCCGGAAGTGCTGAGCTTTCTGAAACGTTGTTTTGATGTTCCGGTGTTTGAAGGTTATGGTTCAACCGAAGCAAGCAGCCTGACCACCGATGGTCGTCTGGATCGTGAACTGGTTACCGAATTTAAACTGGTTGATGTGCCGGAACTGGGTTATAGCGCAACCGATCAGCCGTGTCCGCGTGGCGAACTGCATATTCGTAGCAGTCTGATGGTTCCTGGCTATTACAAAAATGAAAAAGCAACCCGTGCGCTGTTTGATGAAGAGGGTCTGATGAATACCGGTGATATTGTTGAACAGCGTGGACCGGATACCGTTGTTTGGATTGATCGTGCACGTAATGTGCTGAAACTGAGCCAGGGTGAATTTGTTGCGACCTCACGTCTGGAAGTACTGTATAGCGCAGGTAGCCCGTTTCTGCAGCAGATTTTTCTGTATGGTAATAGCACCCGTAGCTATCTGCTGGCAGTTGTTGTTCCTGAACTGCGTGAAATCAGCGCACATCTGCGTCAGCGTGATGTTAAACCGGATGGCGAACCGGTGCGTCAGCTGCTGCGTGCAGAAATTGATCGTATTGCCCGTGAACATCAGCTGCGTGGTTATGAAATTCCTCGTGATTTTCTGATTGAACCGGCACCGTTTACCCGTGCATCAGGTCTGCTGACCGAAACCCAGAAACCGGCACGCGCACGTCTGAAAGCACGTTATGGTGCACGCCTGGAAGAACTGTACGCAACCATTGAACGTACCCAGCTGGAAGAACTGCGTGGTCTGCGTGAAGGTGGTGGTGCAACACCGGCAAGCGCAGCACTGGCAGTTAAAAAAGCACTGGAAGCAACCCTGGGCATTACCGGTGTTGAACCGCGTAGCGCACGTAGCTTTGCACAGCTGGGTGGTGATAGCCTGAGTGCAGTGCGTCTGAGTCGCCTGATTGAAGAAATTAGCGGTGTTGCCGTTCCGGTTGGTCTGGTTCTGAATCCGACCAGCAGCGTTCGTGCAATTGCCGATCATCTGGAACATGCCCTGGCAGGCGAAGCACCGCGTCGTGCCGCACGTTTTGATGAGGTTCATGGTGCGGGTGCCGAAGTTGTTCGTGCAGCCGATCTGCGCCTGGATCGTTTTCTGGGTCCGGATGAACTGGCAGCAGCCCGTCGTAGCACACCGGCAGCAGCTCTGCCAGCACAGGCACGTGTTGCACTGCTGACAGGTGCAAATGGCTTTCTGGGACGCTTTCTGGCACTGGAACTGCTGGAACGTCTGCCGGAAGAGGGACGTCTGTATTGTGTTGTTCGTTCACCGGATGATGCACTGGCATTTGATCGCCTGCGTGCAACCTATGAAAGCGATCCTGCCCTGCTGGAACGCTTTGATGCACTGAGCGCACATGGTCGCCTGGTTGTTCTGGCTGGTGATCTGGTGGAACCGCGTTTTGGTCTGGCAGATGATCTGTATGCACATCTGTGTGTTGAAGTTGATTGCGTTGTTCATAATGGTGCCCTGGTTAATCATGCCCTGAGCTATCCGCAGCTGTTTGAACCGAATGTTCTGGGCACCGTTGAAGCAATTCGTCTGTCACTGGCACATCGTGTTAAAAGCATGAATTATGTTAGCACCATTGCAGCCGTTGGTGGACTGGATCGTAGCGGTCCGATTCGTGAAGATGAAGATATCCGTGAACTGTGGCCTGAACGTGCGCTGGGAGCAGGTTATGCAGTTGGTTATGCAACCAGCAAATGGGCAAGCGAAGTACTGCTGCAGGATGCACATGATGCCCTGGGTCTGCCGGTTAATGTTTATCGTCCGAGCGGTATTATGGCACATAGCCTGTATCGTAGTCAGATTAATGTTCCGGATTTTTTCACCCGTCTGCTGTGTGGTATTGTTTATACCGGTCTGGCACCGCGTAGTTTTTATGAAGGTGGCCGTCCGCATCGTGCAGGTCATTATGATGGCCTGCCGGTTGATGCCGTTGCCCGTAGCATTGCCGCAGTTGCAGTTGATCGTCGTCCGCCAGCCGGTGATGAAGGTGAACGCGCACGCCGTGCCACCTATCATGTTGTTAATCCGCATTGGGATGATGGTATTAGCCTGGATGTTATTGTTAGCTGGGTTCGTAGTGCAGGTTATCCGGTTGAACGTGTTGATGATTATGCAGCATGGTATGCAGCCTTTCGCGATCGCCTGATGCAGCTGAGTGAACCGCTGCGTCGTCATTCACCGCTGCCGATTCTGAATGCATGGGAACGTCCGGCACGTGCGGATGGTGAAGTTTTTGATGCAGAGCGCCTGCTGGCACGCCTGCGCCAGCTGGCTGCACATGGCGGAGCTGGCGATCTGGCAACCCTGCCTCATGTTACCGAACCGCTGATTCATAAATATCTGGATGATATGGTTGCGCTGGGTCTGATTGGTCCGGCAGCCGTGCGTGCAGCAAGCTAA

Cs2_ACAR

ATGACCGAAAATGGTGGTCTGCCGCAGGTTAATGGTGGTGGTCCGCAGTATTGGGAAATGAGCAATGGTCAGAATCAGACCAATAACGTGATTCAGAAACGTCTGGCACGTGCGCGTGAAGTTATTAAACAGGATCCGCAGCTGCAGGCAGCAAAATTTGATCGTAAAAGCCTGCAGCGTATTACCGATGCAGGTAATACCAGCATTGAAATTATTGCAGCCATGTTTAAAGAATACGCAAGCCGTGACCTGTTTGGTGCATGTACACCGGGTGAAAGCACCTTTCATACCGTTACCTATGGTGCAGTTTGGGAACGTATTCAGGCACTGGTTGCAGGTTGGACCGCACTGGGTTTTGTTGCTCCGGGTGATTTTGTTGGTATTAGCGGTTTTGCAAGCGTTGATTGGGTTGTTAGCGATCTGGCAACCCTGCATGCCGGTGGTGTTATGGTTCCGCTGCCGACCAATATTCTGGCCGAAGATGTTCGTGCAATTATTGATGAAGCCGAAGTTCGTTGTCTGATGGTTAGCGCAGAAGAACTGGCAGCAATTGCACCGGTTATTGGTGGTTGTGCCAGCGTTAAAGCAGTTATTGTTATGGATAGCAGCACCGATGCCGTTACCAGCAGCGGTGCATATGCCGAAATGCAGGCCAATCTGCCTGCCGGTGCAAAACTGACCACCATCGATGAAGTTCTGGCAGCAGGTCGTGCAACCGGTAAACAGCCTGCCCTGGTTATTCCGGGTCGTGATGGTCGTCCGGCAGATCCGCTGGTTAATCTGATGTATACCAGCGGTAGCAGCGGTCGTCCGAAAGGTGCAGAATATCCGGAACATCTGATTTTTGATTTTCTGAAAAATAGCATGCCGACCGATGCACCGGAACTGCCGACGATTATTATGGGTTTTCTGCCGCTGAATCATCTGATGGGTCGTTTTACCCTGCTGAAATGTCTGCTGACCGGTGGCCAGAATTGGTTTGTTCGTAGCACCGATATGAGCACCTTTTTTGATGATCTGGCGACCATTCGTCCGACCGAAGCAATGTTTCCGCCTCGTATTATGAATATGCTGCATGATCGTTTTGTGGAACAGCTGGATCGTCTGCCTCCGGCACCGAGCGAAGCAGAACGTGCACAGCAGCGTCAGGATCTGATTAAACGTTTTCGTGAAGTTGATCTGGGTGGTCGTCTGTTTACAGGTAGCTTTGGTAGCGCACCGGCAAGTCCGGATGTTATTCAGTGGCTGGAAGAAGTGCTGGGTTTTCCGCCTGTTAATGGTTATGGTAGTACCGAAGGTGGTATGATTATGCTGGACAACAAAATTCAGCACAGCTATGTTCCAGCCTATAAACTGGTTGATGTGCCGGAACTGGGTTATACCACCAAAGATAAACCGTTTCCGCGTGGTGAACTGCGTATTAAAACCCGTCGTATGATTCCGGGTTATTACAAACATCCTGAAGCAACCGCAGACCTGTTCGATGAAGAAGGTTTCCTGAAAACCGGTGATGTTGTTGAACAGCGTGATGCAGATACCTTTATTTGGCTGGATCGCGTGAAAAACATTATCAAACTGAGCCAGGGTGAATATGTTAGCGTTAGCCGTCTGGAAGAAATTTATGTGGGTAACAGCAAACTGATTCACCAGATGTATATCTATGGTAATAGCCTGCGTGCATATCTGGTTGCCGTTGTTGTTCCGCATATTGAAAATGGCGCATGTGCAGATGCCGGTAAACTGCGTGCAGCACTGCGTACCGAACTGGATGATGTTGCACGTCGTAAAGCACTGCAGGGTTATGAAATTCCTCGTGAATTTATTGTTGAGATGCGTCCGTTTAGCAAAGATAATCATCTGCTGACAGATAGCGCAAAACCGGCACGTGGTCAGCTGAAAAAACGTTATCAGGCAGAACTGGAAGGTCTGTATACGGCACTGGAAGAACGTCTGCGTGAACGGCTGCGTGCAATGAAAGAAGGTAAAGACGCAAGCGTTCAGGATCGTATCAAACAGGCCCTGGAAGTTACCCTGGGTCTGGCGGAAGAGGATATGGCAGATGTTGCAAGCCGTAGCTTTGCACAGCTGGGTGGTGATAGCCTGGCAGCCATTCAGTTTGCACGTTATGTTGGCGAACTGTGTGGTGTTAATCTGCCGGTTAGCTTTGTTCTGGATCATAGCCATAGTCTGCAGGCAATTGCAGATCGTGTGCATGAACTGGTTAGCGGTGATGCCTCAGCAGGCATTACCTTTGAAAGCATTCATGGTAGTGATGGCGTTAACATTAAAGCAGCAGATCTGAAACTGGATCGTTTTCTGAGCGAAGCCGATACCGCAGCAGCAGCCGCTGCAGCACCGGCATCAGAACTGCCTGCACGTCCGACCCATGTTCTGCTGACTGGCGCAAATGGTTTTCTGGGTCGCTTTCTGCTGCTGGATCTGCTGCAGCGTGGTAGCGATAAAAATGGCGGTCGTGTTGTTGCAATTGTTCGTGGTAGCAGTGATGAAAAAGCAGCCGAACGCCTGCGTGCGGGTTTTGATAGTGGTGATGCCACCCTGCTGCAACGTTATGATACCCTGAGCAAACATCTGACCGTTTATGCCGGTGATCTGGCCAAACCGCAGCTGGGTCTGAGTCAGGGTGTTTATGAAAGCCTGTGTGCAGAGCTGGATACCATTGTTCATAATGGTGCACTGGTGAACCATGCCTATAGCTATGAGCAGCTGTTTGAACCGAATGTTCTGGGTAGCGTTGAAGTTATGCGTATGGCACTGGCAAAACGCCGTAAAGCCCTGACCTTTATTAGCAGCGTTGGTGTTGTGGGTGGCCTGGATCATCCGCAGCCGGTTACCGAAGCGGAAGATGGTCCGACCCTGTGTGATGTTCATCCTGGTGATGGTGGTTATGCAATTGGTTATGGCTGTAGCAAATGGGCAGTTGAAGTGCTGCTGAAAGAACTGCATCAGCGTTGGGGTGTTCCGGTTAAAGTTTTTCGTTGCGGTATGATTCTGTCCCATACCAGCTATCTGGGTCAGATTAATCCGACAGATTTTTTCACCCGTCTGCTGTGTGGTATTGCATATACAGGTATTGCACCGCAGAGCTTTTATACCCTGCCGCATGGTCCGGAAGAACATTTTGATGGTATGCCGATTGATTTTGTGAGCGGTGTTATTAGCGCAACCACAGCCGCAGAACGTAGCGGCTTTGATACCTATCATGTTGTTAATCCGCATTGGAGTGATGGTGTGAGCCTGGATCGTATTGTAGATTGGGCAGAAAGTGCAGGTTATCCGGTTAATCGTATCGCACCGTACGAACAGTGGTATGCACAGTTTAAAGCAGCCCTGGAAGCACTGGATCATACCCGTCAGCAGCAGTCACCGCTGCCGATTATCTATCAGTGGGAACGTCCGGCAAGCGGTACAAGCGGCACCAAATATGATGCAACACAGCTGCGTAAACGTGCAGCAGCCTATACCCAGTGGAAAGATGTGCCGCATCTGGATGAAGCATTTATTCATCAGAATATGCGTCATCTGACCACCCTGCGTCTGATTACCCCTCCGGGTAAAGCATAA

Lm_ACAR

ATGGAAATTAGCCTGAGCTGCATTGAACTGATTGAACAGTATTGCCTGAAATATGGTGATAAACCGGCACTGGGTTATCGCAAAACCGAAATTACCAATCAGAATCAGAACCAGACCATTCATTATCTGCCGGAATTTGAAACCCTGACCTTTCGTCAGGTTTGGGATCGTATTAGCTATATTGCCAAAGGCTTCAATTATAGCAATCGTGTTAGCCCGAATGATTTTGTTGGTATTTGTGCATTTGGCAGCCCTGAATATATCCTGGCAGAATTTTCATGTCTGTATCTGGGTGCAGTTAGCGTTCCGCTGCAGTTTAACATTAAAGAACCGGAACTGATCAAAATCGTGACCGATACCAAAATGCGTTGTCTGATTACCGGTCTGCAGCAGCTGAAACTGGCATGTAATGCAGTTCCGCATTGTCCGAGCATTCAGAGCATTATTGTTCTGGATTGTCACGAAGAGGATAAAGATCATAGTATTCAGATTCGTACCCTGCGTGCAGATTTTGCGAATCAGAATATTCAGTGTGAACTGCTGACCCTGGCAGATCTGGAACGTGGTGGTCAGCATACCGTTATTGTTAAACCGTTTATTCCGCAGAATAACGCAAATCATCTGGCAACCCTGGTTTATACCAGCGGTAGCACAGGTCTGCCGAAAGGTGCAATGATTACCGAACAGAGCTGGCGTGAACTGTGGACCAAAACCGGCTTCTATAAACATACACCGGATATTCCGCTGATCGTGTTTAATTTCATGCCGCTGAGCCATATGTTTGGTCGTATGATTGTGATTAATAGCCTGATGCAGGGTGGCACCACCTATTTTGCAAGCAAAAGCGATATGAGCACCTTCTTTGAAGAAATTCGTCTGGTTCGTCCGACCGTTCTGTACCTGGTTCCGCGTGTTAGCGAACTGATTTATCAGAACATCCAGATTGCCGTGAATAATCGTCTGCAGGCAAGCAATCCGAGTCCGCGTACCGAAATTGAAAATGAAATTCTGAGCGAAATGAAAAACACCATTCTGGGTGATCGTCTGAGCATTGCAATTACCGCAAGCGCACCGACCCCTCCGGAAATTATGTATTTTCTGAAACGTCTGTTTGACGTGCCGGTGATTGATGTTTATGGTAGCACCGAACTGGGTATTATTCTGATCAATAATCAGACCTCACCGCAGAATGTGATTGCCTATAAACTGGTTAGCCGTCCGGAACTGGATTTTTTCACCACCGATAAACCGTATCCGCGTGGTGAACTGTATATGAAAACCCATCGTGCAGTGATTGGCTATTATCAGAATCCGGATGCAACAGCCGACCTGTTTGATGCAGAAGGTTATCTGAAAACCGGTGATGTTGTTGAAGAACGTGCACCGAATAACCTGTTTCTGATTGATCGCATCAATAACATCATCAAACTGGGCAATGGTGAATTTGTTAGCCTGCTGCGTCTGGAACAAATTTTTCTGGGTGGTAGCCGTCTGATCAAACAACTGTTTCTGTATGGTAGCTCACTGCGTAGCTATCTGCTGGCAGTTCTGGTTCCGGATATTAGTCTGCTGAAAGAAAAACTGATCGAACTGGGCCTGCCGGAAAATCCGGTTCAGATGAAAAAAATCCTGCTGCAAGAAATTCATGCGATTGCCAAACTGGAACAAATCCGTAGCTATGAAGTTCCGCTGGATTTTATCATGGAACTGGAACCGTTTAGCCAAGAAAACCAGCTGATTACGGAAAGCAATAAACTGGCACGCAACAACCTGAAAAAAAAATATGGTCATAGCCTGGAAGAACTGTATGAGCACATTGAGAAAAATCAACTGGCCGATCTGATTAATCTGGGTAATAGCGATAGCAGCATTGAAAATCTGGTTTGTGTTGTTCTGGGCAGCCAGAATATTGAATGGGGTAATAGTAGCTTTATTGATCTGGGTGGCGATAGTCTGGATGCAGTTCGTCTGATGAATACCATCAAAGATATCTATAGCGTTAGCGTGCCGGTTAGCCTGATCCTGAATCCGGCATATAGCATTAAAAACCTGATCAACAACGTGAACGAAAAACGCCATGGTAAAACCGAACGTTGTCGTACCCATGCAATTATCTTTAGCGATATTCATCCGATCAATGCCTCCAAAAGCGAAAAAATGAAACTGTTTGCAAGCGATCTGCATCTGAGCGCATTTTTTAGCAGCCATGAAATGGAACAGGCAAAAAAACTGCCGAAACGTGATAAAAGCCTGCCGGTGAGCAATGTTCTGCTGACCGGTGCAAATGGTTATCTGGGTCGTTTTCTGGCACTGGCACTGCTGGAAAAACTGTCACTGAATCAGGGTAAACTGATTTGTTTTGTTCGTGCGCATAATAATACGCATGCACGTAAACGCATGTTTGAAAGTTTTCAGCATAGCGAATTTGCACTGCGTGAACGTTTTATTCAGCTGGCACAGAACCATCTGGTTGTTTATGCAGCAGATCTGACCGCACCGAATTTTGGTCTGAGTGATGATGTGTATGATACCCTGGCAACCGAAGTTGATCTGGTACTGCATAATGGTGCACTGGTTAATCATGCACTGAGCTATGAGCAGCTGTTTGAACCGAATGTTCTGGGTACAGTTGAAGTTATTCGTTTTGCCCTGCAGAATAAACTGAAACCGATTCATTTTGTGAGCAGCGTTGCAGTTACCAAAGGTCTGTATCATCGTAGCATTATTCTGGAAACCGAAGATATTCGTAAACAGTGGCCGATGCGTTATAACAAAACCGGTTATGCCGAAGGCTATGCAACCAGCAAATGGGCAGGCGAAGTGCTGCTGAAAAATTTCTCAGAAGCATTTGATCTGCCGGTGCATGTGTTTCGTTGTGGTATGATTCTGGCACATAGCACCTATCTGGGCGAAATTAATGCAGATGACTTTTTTACCCGTCTGATGGCAGGTATCATTAATACCGGCATTGCACCGAAAACATTTTATGAAGTTACCCACGAAACCGACTTTAAACCGTGTTTTGATGGTCTGCCGGTTGATTTTATTGCAGATACCATTGCAGATGATGCCCTGGTTTTTGGCACCGATTATGCCACCTATTGTGTTATTAATCCGACCGGCAATCGCTCAATTAACCTGGATGTTATTATCAACTGGGTGATTGAGTTCGGCTATAAAATCGACCAGCTGGAATTCTATGAAGAATGGTATCGTGAATTTCTGACACGCCTGAAACAGCTGAGCGGTATTCAGAAACGTCATAGTCCGCTGCCGATTATCCATAAATGGGAACATCCGGCAGAAATTGGTCATGAAAGCAAACTGGATAACAGCCAGTTCTATAAAATGATCAAAAAACACGTGGATAGCAACGTGCCGATCATTAATCAGGCCTTTATCTACAAATGCCTGATCGATATGGCAATGCTGAAAATTATCGGCCTGCCGCAGGCAATTCAGAGCTAA

Nc_ACAR

ATGAGCCAGCAGCAGAATCCGCCTTATGGTCGTCGTCTGATTCTGGATATTATCAAAGAACGTGCACTGAATGAACCGAATCGTGAATGGGTTAGCGTTCCGCGTAGCAGCGATCCGAAAGATGGTTGGAAAATTCTGACCTATCTGGATGCCTATAATGGCATTAATCGTGTTGCACATAAACTGACCCAGGTTTGTGGTGCAGCAGCACCGGGTAGCTTTCCGACCGTTGCATATATTGGTCCGAATGATGTTCGTTATCTGGTTTTTGCACTGGGTGCAGTTAAAGCAGGTTATAAAGCACTGTTTATTAGCACCCGTAATAGCGCAGAAGCACAGGTAAACCTGTTTGAACTGACCAATTGTAATGTGCTGGTGTTCGATCAGAGCTATAAAGCAACCGTTCAGCCGTGGCTGCATGAACGTGAAATGACCGCAATTCTGGCACTGCCTGCAGATGAATGGTTTCCGGCAGATCAAGAAGATTTCCCGTATAACAAAACCTTTGAAGAGGCAGAATGGGATCCGCTGATGGTTCTGCATACCAGCGGTAGCACCGGTTTTCCGAAACCGATTGTTGCACGTCAGGGTATGCTGGCAGTTGCAGATCAGTTTCATAATCTGCCTCCGCGTGAAGATGGTAAACTGATGTGGATTGTTGAAATGAGCAAACGTGCAAAACGTCTGATGCATCCGATGCCGCTGTTTCATGCCGCAGGTATGTATATTAGCATGCTGATGATTCACTATTGGGATACACCGGGTGCCCTGGGTATTGGTGAACGTCCGCTGAGCAGTGATCTGGTTCTGGATTATATTGAATATGCCGATGTGGAAGGTATGATTCTGCCACCGGCTATTCTGGAAGAACTGAGCCGTGATGAAAAAGCAATTCAGAGCCTGCAGAAACTGAACTTTGTTAGCTTTGGTGGTGGTAATCTGGCACCGGAAGCCGGTGATCGTCTGGTTGAAAATAATGTTACCCTGTGCAATCTGATTAGCGCAACCGAATTTACCCCGTTTCCGTTTTATTGGCAGTATGATCAGAAACTGTGGCGCTATTTTAACTTTGATACCGACCTGTTTGGTATCGATTGGCGTCTGCATGATGGTGAAAGCACCTATGAACAGGTTATTGTGCGCAAAGATAAACATCCGGGTCTGCAGGGTTTTTTCTATACCTTTCCGGATAGCAGCGAATATAGCACCAAAGATCTGTATAAACGTCATCCGACCCATGAAGATTTTTGGATTTATCAGGGTCGTGCCGATAACATTATCGTGTTTAGCAATGGCGAAAAACTGAACCCGATTACCATTGAAGAAACACTGCAGGGTCATCCGAAAGTTATGGGTGCCGTTGTTGTTGGCACCAATCGTTTTCAGCCTGCACTGATTATTGAACCGGTTGAACATCCGGAAACCGAAGAAGGTCGTAAAGCCCTGCTGGATGAAATTTGGCCGACCGTGGTTCGTGTTAATAAAGAAACCGTGGCACATGGTCAGATTGGTCGTCAGTATATGGCACTGTCAACACCGGGTAAACCGTTTCTGCGTGCAGGTAAAGGCACCGTTCTGCGTCCGGGTACGATTAATATGTATAAAGCCGAGATCGATAAAATCTATGAGGATGCCGAAAAAGGTGTTGCAACCGATGAAGTTCCGAAACTGGATCTGAGCAGCAGTGATGCCCTGATTGTTAGCATTGAAAAACTGTTTGAAACCAGCCTGAACGCACCTAAACTGGAAGCAGATACCGATTTTTTCACCGCAGGCGTTGATAGCATGCAGGTTATTACCGCAAGTCGCCTGATTCGTGCAGGTCTGGCAGCAGCCGGTGTTAATATTGAAGCAAGCGCACTGGCAACCCGTGTTATTTATGGTAATCCGACCCCGAAACGTCTGGCCGATTATCTGCTGAGCATTGTGAATAAAGATAGCAATCAGGGAACCCTGGATAATGAACACCATGTTATGGAAGCACTGGTGGAAAAATACACACGCGATCTGCCGACACCGAAACAGAATAAACCGGCACCGGCAGATGAAGGTCAGGTTGTTGTGATTACCGGTACAACCGGTGGTATTGGTAGCTATCTGATTGATATTTGTAGCAGCAGCAGTCGTGTGAGCAAAATTATCTGTCTGAATCGTAGCGAAGATGGCAAAGCACGTCAGACCGCAAGCAGCTCAGGTCGTGGTCTGAGCACCGATTTTAGCAAATGTGAATTTTATCACGCCGATATGAGCCGTGCAGATCTGGGTCTGGGTCCGGAAGTTTATAGCCGTCTGCTGAGTGAAGTTGATCGTGTTATTCATAATCAATGGCCTGTGAACTTTAACATTGCCGTGGAAAGCTTTGAACCGCATATTCGTGGTTGTCGTAATCTGGTTGATTTTAGCTACAAAGCCGATAAAAATGTGCCGATTGTTTTTGTTAGCAGCATTGGCACCGTTGATCGTTGGCATGATGAAGATCGTATTGTTCCGGAAGCAAGCCTGGATGATCTGAGCCTGGCAGCGGGTGGTTATGGTCAGAGCAAACTGGTTAGCAGCCTGATTTTTGATAAAGCAGCCGAAGTTAGCGGTGTTCCGACCGAAGTTGTGCGTGTTGGTCAGGTGGCAGGTCCGAGCAGTGAAAAAGGTTATTGGAATAAACAAGAATGGCTGCCGAGTATTGTTGCAAGCAGCGCATATCTGGGTGTTCTGCCGGATAGTCTGGGTCAGATGACCACCATTGATTGGACCCCGATTGAAGCCATTGCAAAACTGCTGCTGGAAGTTTCAGGTGTGATTGATAATGTTCCGCTGGACAAAATCAACGGCTATTTTCATGGTGTTAATCCGGAACGTACCAGTTGGAGTGCACTGGCTCCGGCTGTTCAAGAATATTATGGTGATCGCATTCAGAAAATTGTGCCTCTGGATGAATGGCTGGAAGCCCTGGAAAAAAGCCAAGAAAAAGCAGAAGATGTTACCCGTAATCCGGGTATTAAACTGATCGATACCTATCGTACCTGGTCAGAAGGTTACAAAAAAGGCACCAAATTTGTCCCGCTGGATATGACCCGTACCAAAGAATATAGTAAAACCATGCGTGAAATGCATGCAGTTACACCGGAACTGATGAAAAATTGGTGTCGTCAGTGGAACTTCTAA

**Construction of plasmid pVS7-Plac-vanK**

PCR was performed using the genomic DNA of the *C. glutamicum* 2256 strain as the template and synthetic DNAs (vanK-F and vanK-R) as the primers to obtain a PCR product containing the ORF and SD sequences of the *vanK* gene. The PCR product was then inserted into the pVS7 vector [5] and treated with BamH I and Pst I using the In-Fusion HD Cloning Kit. pVS7 is a shuttle vector for coryneform bacteria and *E. coli*. By using this DNA, competent cells of *E. coli* JM109 were transformed, and the cells were applied to the LB medium containing 100 µM IPTG, 40 µg/mL of X-Gal, and 50 µg/mL of spectinomycin, and cultured overnight. White colonies were picked and separated into single colonies to obtain transformants. Plasmids were extracted from the obtained transformants, and the plasmid in which the target PCR product was inserted was designated pVS7-Plac-vanK. In pVS7-Plac-vanK, the vanK gene was expressed under the lac promoter derived from the pVS7 vector.

**Introduction of** **pVK9-ACAR-entD and pVS7-Plac-vanK to *C. glutamicum***

pVK9-ACAR-entD and pVS7-Plac-vanK were introduced into *C. glutamicum* FKFC14 using the electric pulse method. The cells were applied to the CM-Dex agar medium containing 25 µg/ml of kanamycin and 50 µg/mL of spectinomycin and cultured at 31.5 °C. The strains were purified on the same agar medium and used to prepare glycerol stocks.

Supplementary Table 1. Synthetic DNA primers used for PCR.

| primer name | sequence | usage |
| --- | --- | --- |
| DyqhD-F | caggcagatcgttctctgccctcatattggcccagcaaagggagcaagtatctagacgctcaagttag | amplification of a fragment for deleting the *yqhD* gene |
| DyqhD-R | cttaagtctggacgaaatgcccgaaaacgaaagtttgaggcgtaaaaagcagatcttgaagcctgcttttttatac | amplification of a fragment for deleting the *yqhD* gene |
| DvanABK-F1 | cggtacccggggatcCTTACTTCCGCGTATCCAAC | amplification of the upstream region of *vanABK* |
| DvanABK-R1 | CTAGGAATCGCGGCCGGTGAACTCCTAAAGAACTATATAAC | amplification of the upstream region of *vanABK* |
| DvanABK-F2 | GGCCGCGATTCCTAGCATGC | amplification of the downstream region of *vanABK* |
| DvanABK-R2 | ccaagcttgcatgccAGTCATCATCAACGGTGCCG | amplification of the downstream region of *vanABK* |
| DNCgl0324-F1 | cggtacccggggatcGGCATAGTGCTTCCAACGCTC | amplification of the upstream region of NCgl0324 |
| DNCgl0324-R1 | TAGCTCCACTCAAGATTCCTCGATATTACCTACAGG | amplification of the upstream region of NCgl0324 |
| DNCgl0324-F2 | TCTTGAGTGGAGCTAGGGCC | amplification of the downstream region of NCgl0324 |
| DNCgl0324-R2 | ccaagcttgcatgccCATATAGAGCCCAGGAGCTCTC | amplification of the downstream region of NCgl0324 |
| DNCgl0313-F1 | cggtacccggggatcGGGCTCGTCCTGAAATTGCAC | amplification of the upstream region of NCgl0313 |
| DNCgl0313-R1 | TCCGTCGTGAGCCATGTTGTGCCCACGAGACTACC | amplification of the upstream region of NCgl0313 |
| DNCgl0313-F2 | ATGGCTCACGACGGATTGCG | amplification of the downstream region of NCgl0313 |
| DNCgl0313-R2 | ccaagcttgcatgccCGGTTGCAGCCTTCATAAACG | amplification of the downstream region of NCgl0313 |
| DNCgl2709-F1 | cggtacccggggatcAACCCCAGCTCAAATAACACC | amplification of the upstream region of NCgl2709 |
| DNCgl2709-R1 | TTTCAACACAATCCGTCCTTCTCGCTTGGATTACTTG | amplification of the upstream region of NCgl2709 |
| DNCgl2709-F2 | CGGATTGTGTTGAAATTGCTCTG | amplification of the downstream region of NCgl2709 |
| DNCgl2709-R2 | ccaagcttgcatgccTCACCACGGGAATCTTCAGG | amplification of the downstream region of NCgl2709 |
| DvanABK-CF | ATCTCCGCAGAAGACGTACTG | confirmation of the deletion of vanABK |
| DvanABK-CR | TCCGATCATGTATGACCTCC | confirmation of the deletion of vanABK |
| DNCgl0324-CF | CGCCGCAAAGTCCAAATAGAAAG | confirmation of the deletion of NCgl0324 |
| DNCgl0324-CR | GGATTCTTCCTGAACTCAGC | confirmation of the deletion of NCgl0324 |
| DNCgl0313-CF | AGACCAATGAGTACCCAACCG | confirmation of the deletion of NCgl0313 |
| DNCgl0313-CR | TCAGCGTCTGGCTCAGCTAC | confirmation of the deletion of NCgl0313 |
| DNCgl2709-CF | CCGGACTGGGGTGTGTTTTG | confirmation of the deletion of NCgl2709 |
| DNCgl2709-CR | CCCGGAAAATACGGTATAGC | confirmation of the deletion of NCgl2709 |
| Ptuf-F | CcaagcttgcatgccAGATCGTTTAGATCCGAAGG | amplification of Ptuf gene fragment |
| Ptuf-R | TGTATGTCCTCCTGGACTTC | amplification of Ptuf gene fragment |
| Nb_ACAR-F | CCAGGAGGACATACAatggcaactgacagcaggag | amplification of *Nb_ACAR* gene fragment |
| Nb_ACAR-R | GTATGTCCTCCTttataacagacctaaatgacg | amplification of *Nb_ACAR* gene fragment |
| entD-F | aaAGGAGGACATACAATGGTCGATatgAAAACTACG | amplification of *entD* gene fragment |
| entD-R | cggtacccggggatcCCGCATATCCGGTTGTCAGG | amplification of *entD* gene fragment |
| vanK-F | ccaagcttgcatgccaggaggattataATGCGCCTGCGTGTCTCGAG | amplification of *vanK* gene fragment |
| vanK-R | cggtacccggggatcCAACTACGCGGCGACGTAC | amplification of *vanK* gene fragment |

**References**

1. Stephen VD, Iwatani S, Usuda Y, Matsui K, Nakai Y, Suzuki T, Moriya M, Tsuji Y, Ueda T. Method for producing L-lysine or L-threonine using *Escherichia coli* having attenuated malic enzyme activity. Patent WO2005/010175
2. Datsenko KA, Wanner BL. One-step inactivation of chromosomal genes in *Escherichia coli* K-12 using PCR products. Proc Natl Acad Sci U S A. 2000;97(12):6640-5. doi: 10.1073/pnas.120163297.
3. Koseki C, Fukui K, Nakamura J, Kojima H. Enhanced manufacture of succinic acid with coryneform bacteria. Patent WO2007/046389
4. Hayashi Y, Harada M, Takaoka S, Fukushima Y, Yokoyama K, Nishio Y, Tajima Y, Mihara Y, and Nakata K. Isoprene synthase and polynucleotide encoding same, and method for producing isoprene monomer. Patent WO2013/179722
5. Nishio Y, Yamamoto Y, Yamada K, Yokota K. Manufacture of 2-ketoglutaric acid and derivatives from xylose by fermentation. Patent WO2013/069634
