# Supplementary figures and images for: Vanillin production by Corynebacterium glutamicum using heterologous aromatic carboxylic acid reductases
Source: Biotechnol Biofuels Bioprod. 2024 May 1;17:58. doi: 10.1186/s13068-024-02507-3 (PMC11064420; doi:10.1186/s13068-024-02507-3)

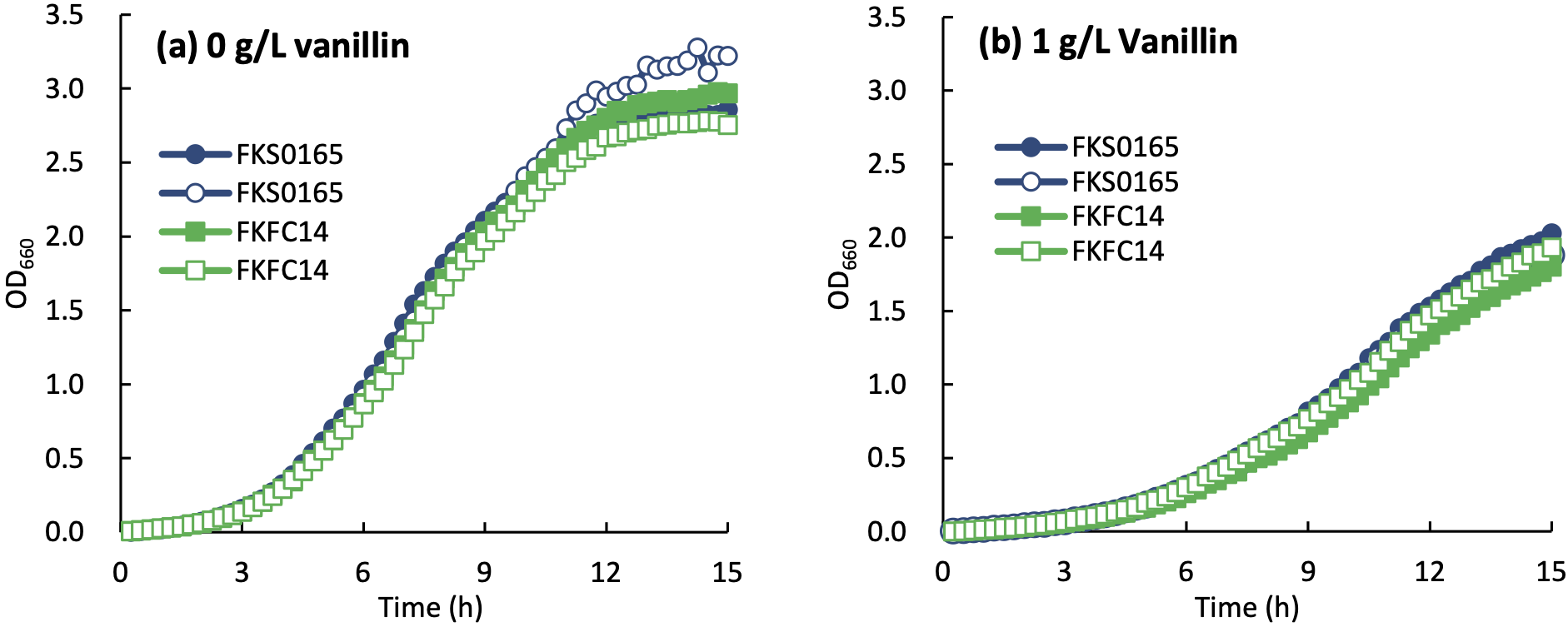

Supplement: Supplementary file 2 — Additional file 2: Figure S1. Influence of aromatic aldehyde reductase (AAR) deletion on cell growth. FKS0165 (open and closed blue circle) and FKFC14 (open and closed green square) were grown in CM-Dex medium containing a 0 g/L vanillin and b 1 g/L vanillin. Experiments were performed in duplicate. [file 13068_2024_2507_MOESM2_ESM.tiff]

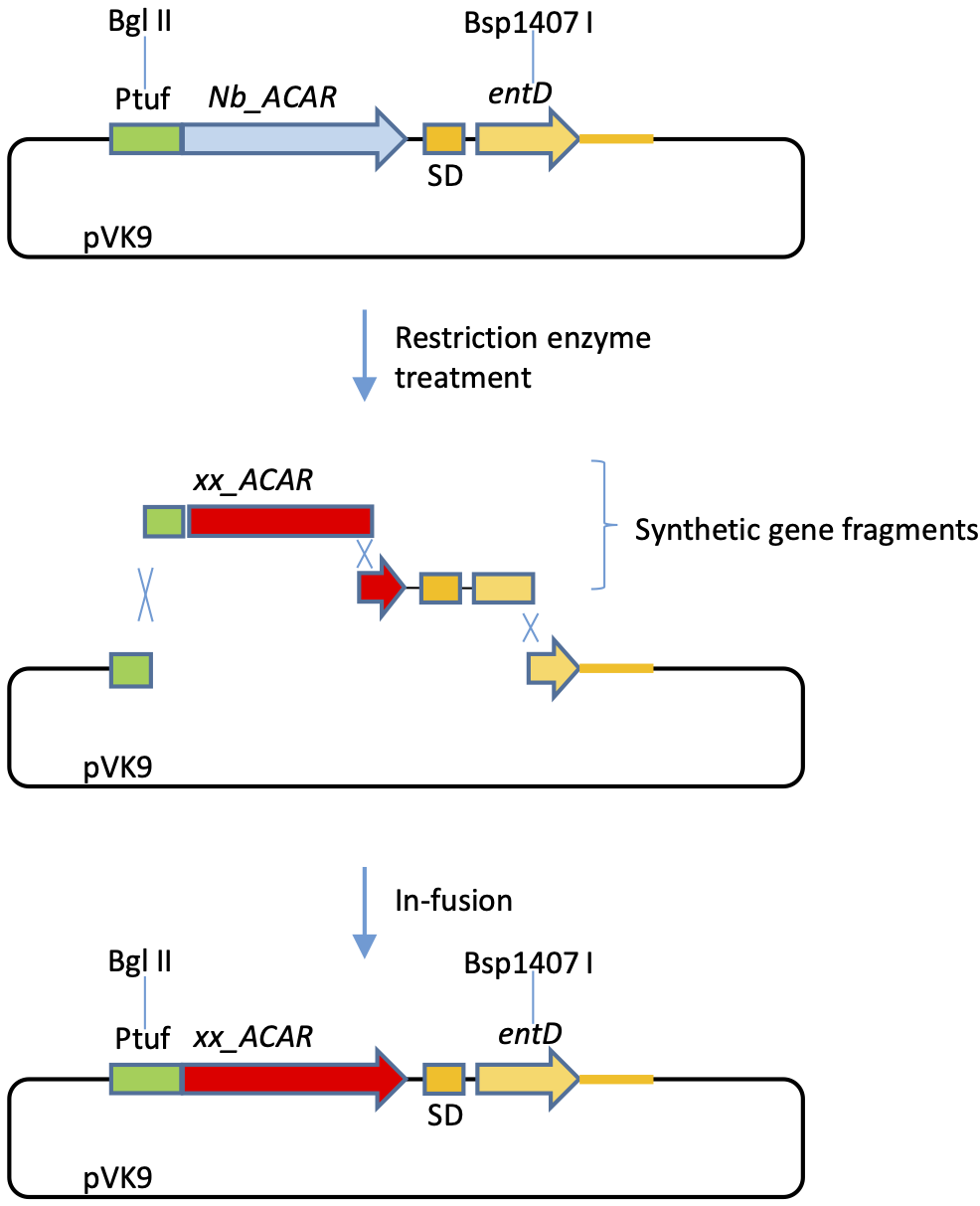

Supplement: Supplementary file 3 — Additional file 3: Figure S2. Construction of ACAR-entD expression plasmids. pVK9-Nb_ACAR-entD was treated with Bgl II and Bps1407 I and was connected to two synthetic DNA fragments with partial sequences of Ptuf at the 3′ end, ACAR genes (codon-optimized to Escherichia coli), and partial sequence of entD at the 5′ end using the In-Fusion HD Cloning Kit. [file 13068_2024_2507_MOESM3_ESM.tiff]
